# The *p*-Terphenyl and Kavalactone Secondary Metabolites from the Fungus *Hydnellum aurantiacum*: Isolation and Evaluation of Their Effects on Platelet Activation

**DOI:** 10.3390/molecules31122175

**Published:** 2026-06-21

**Authors:** Nikita Pronin, Anastasiia O. Whaley, Andrei Whaley, Vladislav Zhuravlev, Sergey Smirnov, Sergey Volobuev, Stepan Gambaryan

**Affiliations:** 1 Sechenov Institute of Evolutionary Physiology and Biochemistry of the Russian Academy of Sciences, Saint Petersburg 194223, Russia; 2 Department of Pharmacognosy, Saint Petersburg State Chemical and Pharmaceutical University, Saint Petersburg 197022, Russia; anastasiya.ponkratova@yandex.ru (A.O.W.); 9968639@gmail.com (A.W.); vladzhuravlev3@gmail.com (V.Z.); 3 The Research Resources Center for Magnetic Resonance, Saint Petersburg State University, Saint Petersburg 198504, Russia; sergey.smirnov@spbu.ru; 4 Komarov Botanical Institute of the Russian Academy of Sciences, Saint Petersburg 197022, Russia; sergvolobuev@mail.ru

**Keywords:** *p*-terphenyls, kavalactones, *Hydnellum aurantiacum*, platelets, platelet activation, isolation, structural elucidation

## Abstract

Phytochemical analysis of the tooth fungus *Hydnellum aurantiacum* resulted in the isolation of twenty-two compounds, including two new kavalactone derivatives—methylkavain (**1**) and aurapyrone (**2**); seven new *p*-terphenyl derivatives—ethylatromentin (**3**)**,** 2-O-benzoylatromentin (**4**), aurantin (**5**), leucohydnelin (**6**)**,** leucoaurantin (**7**), hydroxyleucoaurantiacin (**8**), benzoyltelephantin M (**9**); and thirteen known *p*-terphenyls—atromentin (**10**), aurantiacin (**11**), telephantin K (**12**), leucoatromentin (**13**), telephantin J (**14**), curtisian A (**15**), concrescenin B (**16**), telephantin L (**17**), dihydroaurantiacin dibenzoate (**18**), telephantin M (**19**), sarcodonin α (**20**), sarcodonin δ (**21**) and phellodonin (**22**). The structures were elucidated using spectroscopic methods (UV, NMR, HR-ESI-MS) along with comparison to literature data. All isolated substances, except **8** and **10**, affected thrombin-induced human platelet activation at 90 μM: the seven compounds (**9**, **12**, **16**, **17**, **20**, **21**, **22**) potentiated it, while the remaining ones exhibited inhibitory activity with the strongest antiplatelet effects observed for **4**, **5**, and **7** (10.6 ± 4.0, 4.3 ± 3.2, 9.6 ± 2.9% of positive control, respectively). These and other *p*-terphenyl derivatives with antiplatelet activity identified in this study represent promising structures for further investigation into the mechanism of their action.

## 1. Introduction

Cardiovascular diseases, especially ischemic heart disease (IHD), remain among the major causes of mortality worldwide [1]. A key pathogenetic factor of IHD development is endothelial dysfunction, which is mainly driven by oxidative stress and hyperlipidemia [2]. Under physiological conditions, the intact endothelium releases prostacyclin (PGI_2_) and nitric oxide (NO) to prevent platelet activation and subsequent thrombus formation [3]. Endothelial dysfunction disrupts the balance between platelet inhibitors (PGI_2_, NO) and activators (thrombin, thromboxane A_2_, collagen, ADP), promoting thrombosis and severe complications, such as unstable angina, myocardial infarction, ischemic stroke, and critical limb ischemia [4]. Antiplatelet therapy with aspirin and/or P2Y_12_ receptor inhibitors (clopidogrel, prasugrel, ticagrelor) is the standard of care for the secondary prevention of thrombotic complications in IHD [5]. However, its use is associated with the risks of drug resistance [6] and development of side effects, including peptic ulcers, gastrointestinal bleeding, intracranial haemorrhage, and aspirin hypersensitivity reactions [7,8,9]. Thus, discovering novel compounds with antiplatelet activity remains highly relevant.

Remarkably, natural products and their derivatives constitute more than 50% of modern drugs [10]. Fungi, along with plants, undoubtedly represent valuable sources of biologically active natural compounds. Being widely distributed and ecologically diverse, some fungi also contain secondary metabolite gene clusters (SMGCs) enabling the efficient and precise synthesis of multiple metabolites, as revealed by genomic studies [11,12]. Molecules of fungal origin possess numerous beneficial properties, including anticancer, antioxidant, anti-inflammatory, and antiplatelet [13,14,15,16], and are thus of interest in drug discovery studies [17,18,19]. As a result, they could be promising candidates for the development of new drugs.

It is notable that numerous basidiomycetes in *Bankeraceae* (genus *Phellodon*), *Sarcodonaceae* (genera *Boletopsis*, *Hydnellum*, *Sarcodon*) and *Thelephoraceae* (genus *Thelephora*) families along with actinomycetes and some marine fungi contain a wide range of secondary metabolites, especially those of the *p*-terphenyl class. *p*-Terphenyls are a large group of aromatic compounds whose core structure consists of a benzene ring *para*-substituted with two phenyl groups. Molecules of natural *p*-terphenyls are often modified with various substituents, predominantly hydroxy, methoxy, and ethoxy groups; the hydroxyl functions may be esterified with acids (acetic, phenylacetic, benzoic, etc.) or cyclized to form a furan ring [20]. The central benzene ring is frequently found in either the oxidized quinone form or the reduced hydroquinone form; the latter is the defining feature of leuco derivatives [20]. Such structural diversity of natural *p*-terphenyls underlies the broad spectrum of their biological activities. Some of them, especially compounds with methoxy groups (for example, terphenyllin and its derivatives) or polyhydroxy structures containing multiple ester groups (for example, vialinin A and thelephantin O), possess cytotoxic activity against different cancer cell lines [21,22] and demonstrate anticancer effects by inhibiting tumour growth *in vivo* [23,24]. Secondary metabolites of *Hydnellum concrescens* (Pers.) Banker, represented by esterified polyphenolic *p*-terphenyls including concrescenins A/B, thelephantins L/I/K, dihydroaurantiacin dibenzoate, and curtisian A, show significant α-glucosidase inhibitory effects [25]. Additionally, numerous structurally diverse *p*-terphenyls, including para-quinone, polyhydroxy, benzofuranoid, and nitrogenous derivatives, exhibit moderate-to-strong antioxidant activity [26,27,28,29].

However, data concerning the effects of terphenyls on haemostasis and platelets are very limited and fragmented. The significant *in vivo* and *in vitro* antiaggregant activity in the presence of various platelet agonists has been described for two synthetic *ortho*-terphenyls: 3-[2-([1,1′:2′,1″]-terphenyl-4′-yl)ethyl]phenoxyacetic acid and 3-([1:1′,2′:1″]-3′-terphenyl)propanol [30,31]. Atromentin, a natural *p*-terphenyl isolated from *Hydnellum diabolus* Banker, has demonstrated an anticoagulant effect on dog whole blood, in contrast to its synthetic derivatives: dimethylatromentin, 2,5-diphenylbenzoquinone, and polyporic acid [32]. Finally, among natural *p*-terphenyls, influence on platelet signaling pathways has been shown only for curtisian E, a secondary metabolite of *Pseudomerulius curtisii* (Berk.) Redhead et Ginns, which inhibited rat platelet function through the cAMP-mediated VASP phosphorylation and downregulation of several kinases (JNK, ERK2, p38 MAPK, and Akt) [33]. Thus, there is a clear need to investigate the effects of multiple p-terphenyls on human platelets and to establish their structure–activity relationship.

*Hydnellum aurantiacum* (Batsch) P. Karst. is an inedible species of hydnoid fungus (tooth fungus) from the *Sarcodonaceae* family with characteristic, yellowish-cream to orange-coloured basidiomata, which often envelope blades of grass, small twigs and pine needles during their growth. *Hydnellum aurantiacum* is mainly distributed in temperate regions throughout Europe, Asia, and North America. At the same time, the taxonomical history of *H. aurantiacum* is complicated due to the lack of a type specimen for the species. Furthermore, several forms and morphological variations of *H. aurantiacum* were described based on collections from different environmental conditions and geographical areas that cause confusing species identifications [34]. Like all members of the *Sarcodonaceae* family, it belongs to ectomycorrhizal fungi. *Hydnellum aurantiacum* forms ectomycorrhizal associations with conifers, especially with Scots pine (*Pinus sylvestris)* or European spruce (*Picea abies*); therefore, its typical habitats are predominantly coniferous and mixed forests [35,36,37]. Since *p*-terphenyls have been reported to occur in several *Hydnellum* species (*H. caeruleum* (Hornem.) P. Karst., *H. concrescens* (Pers.) Banker, *H. diabolus, H. geogenium* (Fr.) Banker, *H. suaveolens* (Scop.) P. Karst.) [25,32,38,39,40], *H. aurantiacum*, with its vibrant colour, which is attributed to terphenylquinones [41], is considered a rich source of these compounds. Therefore, *H. aurantiacum*, additionally considering its widespread distribution in the boreal forests of Northern Europe and Russia, can serve as a potential reservoir of natural products for bioactivity screening [20].

In this study, we isolated twenty-two compounds, including nine previously undescribed, from the fruiting bodies of *H. aurantiacum*, elucidated their structures using spectroscopic methods (UV, NMR, HR-ESI-MS) and comparison with literature data, and performed flow cytometry analysis of human platelet αIIbβ3 integrin activation to assess their potential antiplatelet activity.

## 2. Results

**Figure 1:**
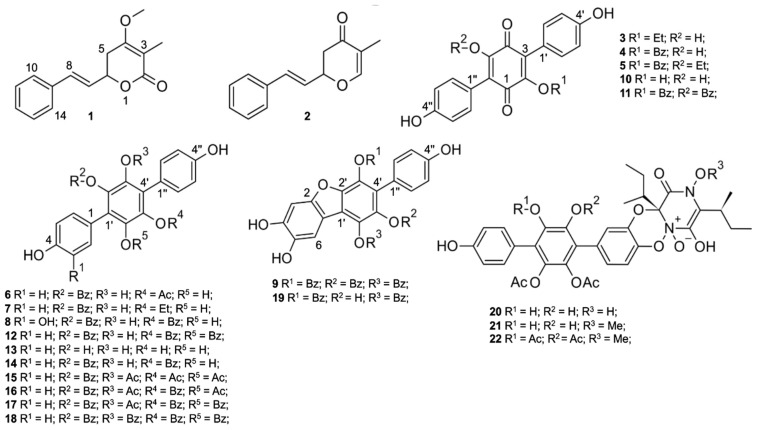
Structure of compounds **1**–**22**.

The new secondary metabolites of *H. aurantiacum* belong to three groups—kavalactones **1**–**2**, atromentin derivatives (terphenylquinones) **3**, **4** [42], **5**, and leuco derivatives **6**–**9**. These new compounds were isolated together with thirteen known *p*-terphenyls: atromentin (**10**) [43], aurantiacin (**11**) [41], telephantin K (**12**) [39], leucoatromentin (**13**) [44], telephantin J (**14**) [39], Curtisian A (**15**) [45], Concrescenin B (**16**) [28], telephantin L (**17**) [39], dihydroaurantiacin dibenzoate (**18**) [46], telephantin M (**19**) [39], sarcodonin α (**20**) [47], sarcodonin δ (**21**) [47], and phellodonin (**22**) [48]. The structures of all compounds are presented in Figure 1.

Kavalactones are styryl pyrone derivatives found in both plants [49] and fungi [50] and since they mostly contain alpha-pyrone cores in their structure, it is of interest that compound **2** is a gamma-pyrone derivative. Isolated atromentin derivatives contain O-ethyl and/or O-benzoyl substituents. The isolated leuco derivatives are presented by O-ethyl, O-acetyl and O-benzoyl derivatives of leucoatromentin and related polyhydroxy 1,1′:4′,1″-terphenyls.

### 2.1. Kavalactones

Compound **1** was obtained as a light-yellow crystalline solid. The HR-ESI-MS analysis at the positive ion mode revealed *m*/*z* 245.1172 [M+H]^+^ (calc. *m*/*z* [M+H]^+^ for C_15_H_17_O_3_^+^ 245.1177) that suggested the molecular formula C_15_H_16_O_3_. ^1^H-^1^H COSY spectra showed that **1** contains two spin systems. The first system consisted of five protons from a mono-substituted benzene ring at *δ*_H_ 7.38 (2H, m, H-10/H-14), 7.50 (2H, m, H-11/H-13) and 7.30 (1H, m, H-12). The second spin system consisted of two styryl *trans*-olefinic protons at *δ*_H_ 6.44 (1H, dd, *J* = 16.0, 6.2 Hz, H-7) and 6.75 (1H, d, *J* = 16.0 Hz, H-8) along with three cyclic aliphatic protons from a substituted dihydro alpha-pyrone at *δ*_H_ 3.01 (1H, dd, *J* = 17.1, 1.8 Hz, H-5a), 2.78 (1H, dd, *J* = 17.1, 11.4 Hz, H-5b) and 5.02 (1H, m, H-6). Additionally, signals from an olefinic methyl group at *δ*_H_ 1.66 (3H, s) and a methoxy group at *δ*_H_ 3.82 (3H, s) were also present.

**Figure 2:**
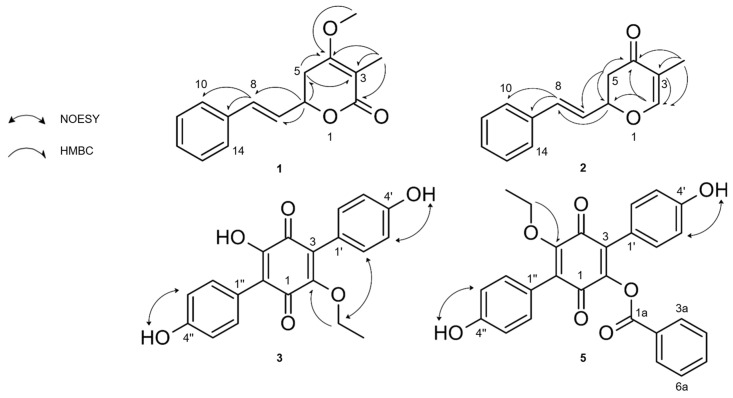
Key NOESY and HMBC correlations for compounds **1**–**3**, **5**.

The ^13^C NMR data (Table 1) revealed the presence of fifteen atoms corresponding to six aromatic, four olefinic, four aliphatic, and one ester. The structure and substitution pattern of the dihydro alpha-pyrone ring was confirmed through HMBC correlations (Figure 2) from 3-Me → C-2, C-3, C-4; 4-OMe → C-4; H-5 → C-3, C-4; H-6 → C-4, C-7, C-8; along with the presence of the styryl group H-8 → C-9, C-10/14. The structure of **1** was confirmed as 3-methyl-4-methoxy-6-[(*E*)-2-phenylethenyl]-5,6-dihydro-2*H*-pyran-2-one and given the name methylkavain.

Compound **2** was obtained as a light-yellow crystalline solid. The HR-ESI-MS analysis at the positive ion mode revealed *m*/*z* 215.1067 [M+H]^+^ (calc. *m*/*z* [M+H]^+^ for C_14_H_15_O_2_^+^ 215.1072) that suggested the molecular formula C_14_H_15_O_2_. ^1^H-^1^H COSY spectra showed that **2** shared the same two spin systems as **1**. The first system consisted of five protons from a mono-substituted benzene ring at *δ*_H_ 7.37 (2H, m, H-10/H-14), 7.49 (2H, m, H-11/H-13) and 7.30 (1H, m, H-12). The second spin system consisted of two styryl *trans*-olefinic protons at *δ*_H_ 6.46 (1H, dd, *J* = 16.0, 6.2 Hz, H-7) and 6.75 (1H, d, *J* = 16.0 Hz, H-8) along with three cyclic aliphatic protons from a substituted dihydro alpha-pyrone at *δ*_H_ 2.70 (1H, dd, *J* = 17.3, 13.4 Hz, H-5a), 2.56 (1H, dd, *J* = 17.3, 3.7 Hz, H-5b) and 5.13 (1H, m, H-6). Additionally, signals from an olefinic methyl group at *δ*_H_ 1.66 (3H, s) and an isolated olefinic proton at *δ*_H_ 7.56 (H, s) were also present.

The ^13^C NMR data (Table 2) revealed the presence of fourteen atoms corresponding to six aromatic, four olefinic, and three aliphatic. The structure and substitution pattern of the dihydro gamma-pyrone ring was confirmed through HMBC correlations (Figure 2) from H-2 → C-3, C-4, C-6; 3-Me → C-2, C-3, C-4; H-5 → C-4, C-6, C-7; H-6 → C-8; along with the presence of the styryl group H-8 → C-9, C-10/14. The structure of **2** was confirmed as 3-methyl-6-[(*E*)-2-phenylethenyl]-5,6-dihydro-4*H*-pyran-4-one and given the name aurapyrone.

### 2.2. Atromentin Derivatives (Terphenylquinones)

Compound **3** was obtained as an orange-brown crystalline solid. The HR-ESI-MS analysis at the positive ion mode revealed *m*/*z* 375.0827 [M+Na]^+^ (calc. *m*/*z* [M+Na]^+^ for C_20_H_16_O_6_Na^+^ 375.0844) that suggested the molecular formula C_20_H_16_O_6_. ^1^H-^1^H COSY spectra showed that **3** was a derivative of atromentin containing three spin systems. Since atromentin has a symmetrical NMR spectrum, an unsymmetrical substitution pattern disrupts this, which is why the closer the proton/C-13 atom is to the unsymmetrical element, the stronger the difference in chemical shifts between previously symmetric atoms/functional groups is. The first two aromatic spin systems contained four protons each and consisted of para-substituted benzene rings at *δ*_H_ 7.17 (2H, d, *J* = 8.5, H-2′/6′), 6.81 (2H, d, *J* = 8.5, H-3′/5′) and 7.20 (2H, d, *J* = 8.5, H-2″/6″), 6.78 (2H, d, *J* = 8.5, H-3″/5″). The third spin system was an O-substituted ethyl group at *δ*_H_ 1.12 (3H, t, *J* = 7.0) and 4.09 (2H, q, *J* = 7.0). Also present were the signals from three phenolic hydroxy groups at *δ*_H_ 9.53 (1H, s, 4′-OH), 9.63 (1H, s, 4″-OH), one of which forms a hydrogen bond with the neighbouring keto group *δ*_H_ 10.55 (H, s, 5-OH).

The ^13^C NMR data (Table 3) revealed the presence of twenty atoms corresponding to twelve aromatic, four olefinic, two aliphatic, and two ketones. Since the compound is an O-ethyl substituted atromentin derivative, there are only two positions where the ethyl group can be located. With the help of NOESY correlations from the methylene group of the ethyl radical to the adjacent H-3″/H-5″ protons and HMBC correlations (Figure 2) from the methylene protons to C-2, the structure of **3** was confirmed as 2-O-ethylatromentin and named ethylatromentin.

Through analysis of NMR data, compound **4** was found to be 2-O-benzoylatromentin, which from what we can tell has previously only been described as a synthetic compound obtained from the benzoylation of atromentin [42]. Therefore, we wish to report this compound as a new natural product from *H. aurantiacum*.

Compound **5** was obtained as an orange-brown crystalline solid. The HR-ESI-MS analysis at the positive ion mode revealed *m*/*z* 457.1284 [M+H]^+^ (calc. *m*/*z* [M+H]^+^ for C_27_H_21_O_7_ 457.1287) that suggested the molecular formula C_27_H_20_O_7_. ^1^H-^1^H COSY spectra showed that **5** was a derivative of atromentin containing four spin systems. The first two aromatic spin systems contained four protons each and consisted of para-substituted benzene rings at *δ*_H_ 7.30 (2H, d, *J* = 8.5, H-2′/6′), 6.81 (2H, d, *J* = 8.5, H-3′/5′) and 7.19 (2H, d, *J* = 8.5, H-2″/6″), 6.83 (2H, d, *J* = 8.5, H-3″/5″). The third spin system was an O-substituted ethyl group at *δ*_H_ 1.17 (3H, t, *J* = 7.0) and 4.17 (2H, q, *J* = 7.0) and the fourth spin system was an O-substituted benzoic acid residue *δ*_H_ 8.01 (2H, m, H-3a/7a), 7.58 (2H, m, H-4a/6a) and 7.75 (1H, m, H-5a). Also present were the signals from two phenolic hydroxy groups at *δ*_H_ 9.85 (1H, s, 4′-OH), 9.71 (1H, s, 4″-OH).

The ^13^C NMR data (Table 4) revealed the presence of twenty-seven atoms corresponding to eighteen aromatic, four olefinic, two aliphatic, two ketones, and one ester. Since the compound is an O-benzoyl-O-ethyl substituted atromentin derivative, it was necessary to find at which phenolic hydroxyl groups were the substituents located. Thanks to the presence of two hydroxyl groups in the spectrum we were able to observe NOESY correlations from 4′-OH and 4″-OH to the adjacent H-3′/H-5′ and H-3″/H-5″ protons respectively, along with HMBC correlations (Figure 2) from the methylene protons of the ethyl group to C-5, which placed the remaining benzoic acid residue in position 2. Therefore, the structure of **5** was confirmed as 2-O-benzoyl-5-O-ethylatromentin and named aurantin.

### 2.3. Leuco Derivatives

Compound **6** was obtained as a white crystalline solid. The HR-ESI-MS analysis at the positive ion mode revealed *m*/*z* 473.1228 [M+H]^+^ (calc. *m*/*z* [M+H]^+^ for C_27_H_21_O_8_ 473.1236) that suggested the molecular formula C_27_H_20_O_8_. ^1^H-^1^H COSY spectra showed that **6** was a leuco-derivative of atromentin containing three spin systems. The first two aromatic spin systems contained four protons each and consisted of para-substituted benzene rings at *δ*_H_ 7.14 (2H, d, *J* = 8.5, H-2/6), 6.70 (2H, d, *J* = 8.5, H-3/5) and 7.10 (2H, d, *J* = 8.5, H-2″/6″), 6.81 (2H, d, *J* = 8.5, H-3″/5″). The third spin system was an O-substituted benzoic acid residue *δ*_H_ 7.84 (2H, m, H-3a/7a), 7.51 (2H, m, H-4a/6a), and 7.67 (1H, m, H-5a). Also present were the signals from one acetate residue *δ*_H_ 1.71 (3H, s) and four phenolic hydroxy groups at *δ*_H_ 9.48 (1H, s, 4-OH), 9.46 (1H, s, 4″-OH) and 8.47 (1H, s, 3-OH/6-OH).

The ^13^C NMR data (Table 5) revealed the presence of twenty-seven atoms corresponding to twenty-four aromatic, two esters and one aliphatic. Since the compound is an O-benzoyl-O-acetyl substituted leucoatromentin derivative, it was necessary to find at which phenolic hydroxyl groups the substituents were located. The identical chemical shift values and narrow peak shape of the 3′-OH/6′-OH phenolic hydroxy groups indicate hydrogen bonding with an ortho-acyl group which along with similar chemical shift values for C-1′ (*δ_C_* 123.15) and C-4′ (*δ_C_* 123.08) found through HMBC correlations from H-2/H-6 → C-1′ and H-2″/H-6″ → C-4′ show a uniform para 2′/5′ acylation pattern of the central ring. Therefore, the structure of **6** was confirmed as 4,3′,6′,4″-tetrahydroxy-2′-benzoyl-oxy-5′-acetyl-oxy-[1,1′:4′,1″-terphenyl] (2-O-benzoyl-5-O-acetylleucoatromentin) and named leucohydnelin.

Compound **7** was obtained as a white crystalline solid. The HR-ESI-MS analysis at the negative ion mode revealed *m*/*z* 457.1275 [M-H]^−^ (calc. *m*/*z* [M-H]^−^ for C_27_H_21_O_7_ 457.1287) that suggested the molecular formula C_27_H_22_O_7_. ^1^H-^1^H COSY spectra showed that **7** was a leuco-derivative of atromentin containing four spin systems. The first two aromatic spin systems contained four protons each and consisted of para-substituted benzene rings at *δ*_H_ 7.17 (2H, d, *J* = 8.5, H-2/6), 6.67 (2H, d, *J* = 8.5, H-3/5) and 7.19 (2H, d, *J* = 8.5, H-2″/6″), 6.80 (2H, d, *J* = 8.5, H-3″/5″). The third spin system was an O-substituted ethyl group at *δ*_H_ 0.93 (3H, t, *J* = 7.0) and 3.46 (2H, q, *J* = 7.0) and the fourth spin system was an O-substituted benzoic acid residue *δ*_H_ 7.88 (2H, m, H-3a/7a), 7.48 (2H, m, H-4a/6a), and 7.62 (1H, m, H-5a). Also present were the signals from four phenolic hydroxy groups at *δ*_H_ 9.33 (2H, s, 4-OH/4″-OH), 8.34 (1H, s, 3-OH) and 8.20 (1H, s, 6-OH).

The ^13^C NMR data (Table 6) revealed the presence of twenty-seven atoms corresponding to twenty-four aromatic, one ester and two aliphatic. In comparison to **6**, **7** is also a disubstituted leucoatromentin derivative with similar chemical shift values and narrow peak shapes of the phenolic hydroxy groups at *δ*_H_ 9.33 (2H, s, 3′-OH) and 9.33 (2H, s, 6′-OH) which also indicates hydrogen bonding with an ortho-oxygen-containing functional group. Similar chemical shift values for C-1′ (*δ_C_* 124.79) and C-4′ (*δ_C_* 124.19) found through HMBC correlations from H-2/H-6 → C-1′ and H-2″/H-6″ → C-4′ along with HMBC correlations (Figure 2) from the methylene protons of the ethyl group to C-5 place the remaining benzoic acid residue in position 2. Therefore, the structure of **7** was confirmed as 4,3′,6′,4″-tetrahydroxy-2′-benzoyl-oxy-5′-ethoxy-[1,1′:4′,1″-terphenyl] (2-O-benzoyl-5-O-ethylleucoatromentin) and named leucoaurantin.

Compound **8** was obtained as a white crystalline solid. The HR-ESI-MS analysis at the positive ion mode revealed *m*/*z* 551.1327 [M+H]^+^ (calc. *m*/*z* [M+H]^+^ for C_32_H_23_O_9_ 551.1342) that suggested the molecular formula C_32_H_22_O_9_. ^1^H-^1^H COSY spectra showed that **8** was a leuco-derivative of atromentin containing four spin systems. The first aromatic AMX spin system consisted of a 1,3,4-trisubstituted benzene ring and contained three protons at *δ*_H_ 6.79 (1H, d, *J* = 1.5, H-2), 6.66 (1H, d, *J* = 8.2, H-5) and 6.61 (1H, dd, *J* = 8.2, 1.5, H-6). The second aromatic spin system consisted of para-substituted benzene ring and contained four protons at *δ*_H_ 7.17 (2H, d, *J* = 8.5, H-2″/H-6″) and 6.70 (2H, d, *J* = 8.5, H-3″/H-5″). The last two spin systems were O-substituted benzoic acid residues *δ*_H_ 7.69 (2H, m, H-3a/7a), 7.33 (2H, m, H-4a/6a), and 7.51 (1H, m, H-5a). Also present were the signals from five phenolic hydroxy groups at *δ*_H_ 8.50 (1H, s, 3-OH′), 8.51 (1H, s, 6-OH′), 8.80 (1H, s, 3-OH) and 8.87 (1H, s, 6-OH).

The ^13^C NMR data (Table 7) revealed the presence of thirty-two atoms corresponding to thirty aromatic and two esters. The substitution pattern in ring A was confirmed through HMBC correlations from H-2 → C-1′, C-4; H-5 → C-1, C-4 (C-3), C-6; H-6 → C-1′, C-2, C-3, (C-4). Furthermore, **8** has similar chemical shift values and narrow peak shapes of the phenolic hydroxy groups at *δ*_H_ 8.50 (1H, s, 3′-OH) and 8.51 (1H, s, 6′-OH) which indicates hydrogen bonding with an ortho ester group. Similar chemical shift values for C-1′ (*δ_C_* 123.06) and C-4′ (*δ_C_* 123.37) found through HMBC correlations from H-2/H-6 → C-1′ and H-2″/H-6″ → C-4′ place benzoic acid residues in positions 2′ and 6′. Therefore, the structure of **8** was confirmed as 3,4,3′,6′,4″-pentahydroxy-2′,5′-dibenzoyl-oxy[1,1′:4′,1″-terphenyl] and named hydroxyleucoaurantiacin.

Compound **9** was obtained as a light yellow crystalline solid. The HR-ESI-MS analysis at the positive ion mode revealed *m*/*z* 675.1266 [M+Na]^+^ (calc. *m*/*z* [M+Na]^+^ for C_39_H_24_O_10_ 675.1267) that suggested the molecular formula C_39_H_24_O_10_. ^1^H-^1^H COSY spectra showed that **9** was a leuco-derivative of cycloleucomelone containing four spin systems. The first aromatic spin system consisted of a para-substituted benzene ring with four protons at *δ*_H_ 7.23 (2H, d, *J* = 8.5, H-2″/H-6″) and 7.01 (1H, d, *J* = 8.5, H-3″/H-5″). The other three spin systems were O-substituted benzoic acid residues *δ*_H_ 7.82 (2H, m, H-3a/7a), 7.42 (2H, m, H-4a/6a), 7.60 (1H, m, H-5a); *δ*_H_ 8.10 (2H, m, H-3b/7b), 7.60 (2H, m, H-4b/6b), 7.77 (1H, m, H-5b); and *δ*_H_ 8.07 (2H, m, H-3c/7c), 7.56 (2H, m, H-4c/6c), 7.73 (1H, m, H-5c). Also present were the signals of two protons from a 1,2,4,5-tetrasubstituted benzene ring at *δ*_H_ 7.13 (1H, s, H-3), 7.01 (1H, s, H-6) and three phenolic hydroxy groups at *δ*_H_ 9.79 (1H, s, 4-OH), 9.33 (1H, s, 5-OH) and 9.49 (1H, s, 4″-OH).

**Figure 3:**
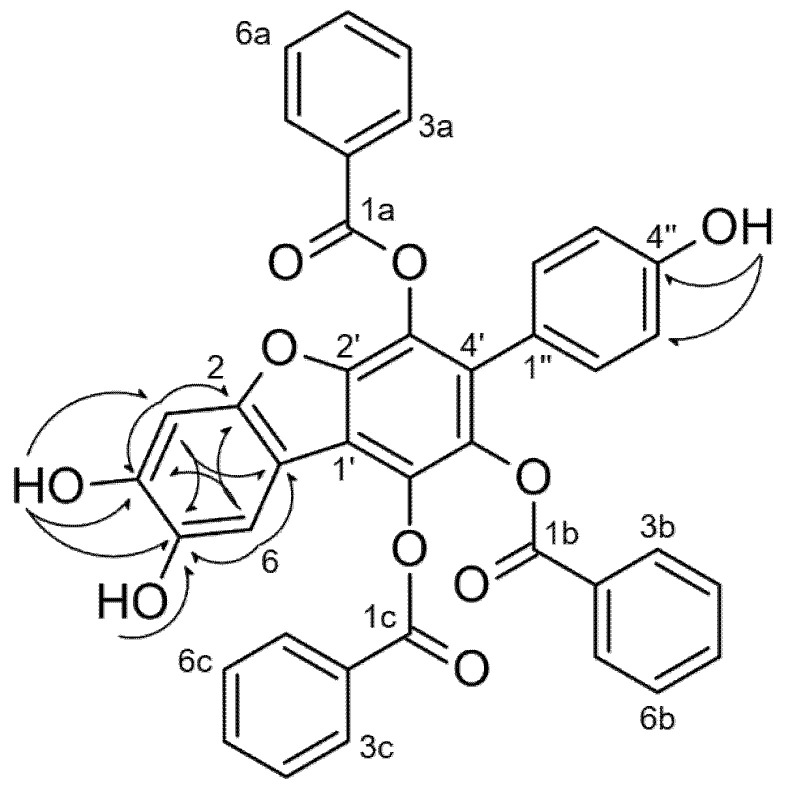
Key HMBC correlations for compound **9**.

The ^13^C NMR data (Table 8) revealed the presence of thirty-nine atoms corresponding to thirty-six aromatic and three esters. The substitution pattern in ring A was confirmed through HMBC correlations (Figure 3) from H-3 → C-1, C-2, C-4, C-5; H-6 → C-2, C-4, C-5, C-1′. The presence of HMBC correlations from phenolic hydroxy protons 4-OH → C-4, C-6; 5-OH → C-3, C-5; and 4″-OH → C-3″, C-4″, C-5″ place the three benzoic acid ester residues in positions 3′, 5′ and 6′ of ring B. Therefore, the structure of **9** was confirmed as 2,2′-epoxy-4,5,4″-trihydroxy-3′,5′,6′-tribenzoyl-oxy[1,1′:4′,1″-terphenyl] and named benzoyltelephantin M.

### 2.4. Effect of Isolated Compounds on Thrombin-Induced Platelet Activation

**Figure 4:**
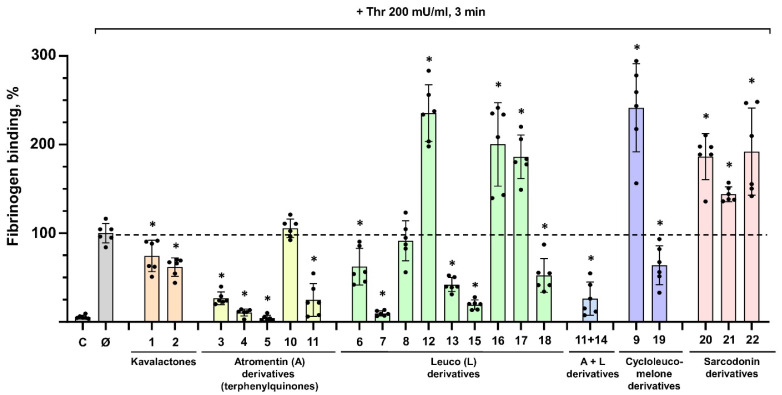
Secondary metabolites of *H. aurantiacum* exhibit various effects on thrombin-induced platelet activation. Washed human platelets were stained with fibrinogen-Alexa-Fluor 647 (15 μg/mL) and then treated with the tested compounds (90 μM) at 37 °C for 30 min. Subsequent platelet activation was induced by the addition of thrombin (200 mU/mL), then the samples were incubated at 37 °C for 3 min. The reaction was stopped by the dilution with phosphate-buffered saline (PBS, 1:10). Data are presented as means ± SD, *n* = 6, Mann–Whitney U-test. * *p* < 0.05 compared to thrombin sample (Ø) designated as 100%.

Fibrinogen binding resulting from inside-out integrin αIIbβ3 conformational change is the endpoint of platelet activation leading to subsequent aggregation and thrombus formation [3]. It has been revealed that secondary metabolites of *H. aurantiacum* possess diverse effects on human platelets (Figure 4). While a minority of them (**8**, **10**) showed no effect, the remaining compounds either inhibited or potentiated thrombin-induced platelet activation. Several leucoatromentin derivatives (**12**, **16**, **17**), a cycloleucomelone *p*-terphenyl (**9**), and all sarcodonin-derived metabolites (**20**–**22**) increased fibrinogen binding by an average of 1.5- to 2-fold compared to the thrombin level (Ø). However, most compounds exhibited inhibitory activity, ranging from moderate to pronounced, with the strongest effects observed for **4**, **5**, and **7** (10.6 ± 4.0, 4.3 ± 3.2, 9.6 ± 2.9% respectively). The kavalactones **1**–**2** showed relatively low inhibitory effects.

## 3. Discussion

A growing body of research indicates that various plant-derived molecules, including phenylpropanoids (flavonoids, coumarins, phenolic acids, curcuminoids, etc.), terpenoids, steroids, and saponins, possess numerous beneficial effects, in particular antithrombotic activity [51,52,53,54]. Nevertheless, this diversity tends to overshadow fungi as another valuable source of active secondary metabolites. Being exceedingly abundant, fungi, including higher ones, produce a vast array of unique and structurally complex compounds. In this study, we isolated and characterised diverse secondary metabolites of *H. aurantiacum*. Since many of them are structurally related derivatives of the sarcodonins, atromentin, leucoatromentin, and kavalactones, we were able to analyse their structure–activity relationships based on the obtained platelet activation assay data.

The sarcodonins are a group of natural products containing a tricyclic benzodioxazine core with a N,N-dioxide ring junction [55]. All isolated sarcodonins—sarcodonin α (**20**), sarcodonin δ (**21**) and phellodonin (**22**)—increased fibrinogen binding to varying degrees. When viewed in relation to sarcodonin α, methylation of one of the N-oxides as seen in sarcodonin δ leads to a reduction in fibrinogen binding, while simultaneous N-oxide methylation and phenolic hydroxyl acetylation observed in phellodonin lead to similar fibrinogen binding as sarcodonin α. Based on these observations, further acetylation of the remaining phenolic hydroxyls in sarcodonin α might increase fibrinogen binding.

With the notable exception of atromentin (**10**), all its derivatives cause a reduction in fibrinogen binding to different degrees depending on the nature of the substituents. Introduction of a benzoyl group in 2-O-benzoylatromentin (**4**) has a stronger effect on decreasing fibrinogen binding when compared to an ethyl group as in 2-O-ethylatromentin (**3**). In comparison, further introduction of a second benzoyl group as in aurantiacin (**11**) has the opposite effect and increases fibrinogen binding. Interestingly, when both a benzoyl and an ethyl group are present in the molecule, as in compound **5**, these further decrease fibrinogen binding in comparison to the monosubstituted 2-O-benzoylatromentin. Thus, a moderate number of hydrophobic substituents might enhance the antiplatelet properties of terphenylquinones.

Out of all the tested compounds, leucoatromentin derivatives have the most complex structure–activity relationships and therefore need to be considered as structurally related sub-groups. Leucoatromentin itself (**13**) decreases fibrinogen binding, while the presence of two acyl substituents in positions 2′ and 5′ as in **6** and **8** causes a relative increase. Interestingly, in similarity to **5**, the presence of both one benzoyl group and one ethyl group in positions 2′ and 5′ as in **7** causes a strong decrease in fibrinogen binding. Starting from curtisian A (**15**), the presence of three acetyl groups and one benzoyl group strongly decreases fibrinogen binding in comparison to leucoatromentin, but as soon as the acetyl group opposite the benzoyl group is substituted for a second benzoyl group as in concrescenin B (**16**) fibrinogen binding drastically increases in comparison to control. The further substitution of one of the remaining acetyl groups for a third benzoyl group as in thelephantin L (**17**) causes a slight relative decrease in fibrinogen binding, but if all acetyl groups are substituted for benzoyl groups this causes a strong decrease in fibrinogen binding in comparison to control. Interestingly, if one of the benzoyl groups is removed as in thelephantin K (**12**), this once again strongly increases fibrinogen binding relative to control. Cycloleucomelone when substituted with three benzoyl groups as in **9** shows the strongest fibrinogen binding, but when one benzoyl group is removed from position 5′ as in telephantin M (**19**), this causes a decrease in fibrinogen binding relative to control.

Since telephantin J (**14**) was isolated as a 1:1 mixture with its oxidized terphenylquinone counterpart aurantiacin (**11**) we thought it would be worthwhile to test the effect this would have on fibrinogen binding in comparison to pure **11**. Notably, the 1:1 mixture of **14** and **11** displayed the same fibrinogen binding as pure **11**, which further highlights the complex structure–activity relationship of leucoatromentin derivatives.

The isolated kavalactones showed a relatively weak decrease in fibrinogen binding in comparison to control. Of note is that the gamma-pyrone **2** showed a stronger decrease in comparison to the alpha-pyrone **1**. It is worth mentioning that the alpha-pyrone kavain, being structurally related to compound **1** and one of the principal components of *Piper methysticum* G.Forst. (kava), was found to inhibit arachidonic acid-induced human platelet aggregation, along with ATP release, thromboxane A_2_ and PGE_2_ formation, as previously reported [56].

Possible mechanisms underlying the effects of *p*-terphenyls on platelets are still unclear. It has been previously demonstrated that curtisian E, isolated from *Pseudomerulius curtisii* (Berk.) Redhead et Ginns, reduced fibrinogen binding and platelet aggregation through the cyclic nucleotide system [33]. Nevertheless, strong dose-dependent cAMP elevation (more than 15-fold at 100 μM of curtisian E) presented by the authors of [33] was not followed by significant VASP phosphorylation at Ser^157^. It is also known that several oxidized *p*-terphenyls possess inhibitory activity against cAMP-specific phosphodiesterases (for example, PDE4B and PDE4D) [57,58,59,60]. However, these data were obtained using isolated enzymes, and it is still unknown whether *p*-terphenyl compounds could affect PDEs in living cells, specifically PDE2A, PDE3A or PDE5A expressed in human platelets, or not. Therefore, the involvement of *p*-terphenyls in cAMP and cGMP-dependent pathways needs further investigation.

## 4. Materials and Methods

### 4.1. Fungal Material

The fruiting bodies of *H. aurantiacum* were collected in the Leningrad region, North-West of the European part of Russia (60°30′49.7″ N, 30°17′22.8″ E), at the end of August 2023. Morphological identification of the fungus was carried out by S.V. Volobuev at the Laboratory of Systematics and Geography of Fungi (Komarov Botanical Institute of the RAS, Saint Petersburg, Russia) based on macro- and micromorphological features using light microscopy technique.

### 4.2. Chemicals and General Experimental Procedures

The chemicals used in this study, along with their respective manufacturers, are listed below: *n*-hexane (JSC LenReactiv, Saint Petersburg, Russia), dichloromethane (JSC LenReactiv, Saint Petersburg, Russia), *n*-butanol (JSC LenReactiv, Saint Petersburg, Russia), Fibrinogen-Alexa-Fluor 647 (Molecular Probes, Gottingen, Germany), thrombin (Roche, Mannheim, Germany).

Analytical high performance liquid chromatography (HPLC) was performed using a Prominence LC-20 with a CTO-20AC column thermostate and SPD-M20A diode-array detector (Shimadzu Corporation, Kyoto, Japan) with a Supelcosil LC18 column (250 × 4.6 mm, I.D. 5 µm). Mobile phase composition was CH_3_CN-H_2_O with 0.1% *v*/*v* TFA using gradient elution from 5:95 to 100:0 with a flow rate of 1 mL/min, column temperature −40 °C and 10 µL injection volume. Preparative HPLC was performed using a Smartline Preparative Pump 1800 with a Smartline UV/VIS Detector 2520 spectrophotometric detector (Knauer, Berlin, Germany) with a Kromasil C18 column (250 × 30.0 mm, I.D. 5 µm). Mobile phase composition was CH_3_CN-H_2_O with 0.1% *v*/*v* TFA using gradient elution from 10:90 to 100:0 with a flow rate of 40 mL/min, column temperature −40 °C and 1 mL injection volume. HPLC grade solvents used for analytical and preparative HPLC analysis were J.T. Baker (Avantor, Radnor, PA, USA) HPLC gradient grade. Open column chromatography (CC) was performed on Sephadex LH-20 (Cytiva, Marlborough, MA, USA) using a mobile phase composed of 95% EtOH (isocratic elution), and on silica gel using a DCM-EtOH mobile phase from 100:0 to 50:50, *v*/*v*, with a step of 10%. HR-ESI-MS data were obtained using a Q-TOF LCMS-9030 (Shimadzu Corporation, Kyoto, Japan) through direct injection of 1 µL sample volume with 4.0 kV ion spray voltage, 180 °C ion source temperature, 3 L/min nebulizer gas and 10 L/min drying gas flow rate. The 1D and 2D NMR spectra were acquired using a Bruker Avance III 400 NMR spectrometer (Bruker, Billerica, MA, USA).

### 4.3. Extraction and Isolation of Compounds

Dried and ground fruiting bodies of *H. aurantiacum* (700 g) were extracted (10×) with 3000 mL 96% EtOH at room temperature using maceration; the isolation flowchart is presented in the Supplementary Materials (Figure S62). The extract was evaporated to 500 mL and subjected to sequential liquid–liquid extraction using equal volumes of *n*-hexane (5×), dichloromethane (DCM) (5×) and *n*-butanol (5×). The resulting dichloromethane fraction was evaporated to 80 mL and further subjected to column chromatography (CC) with Sephadex LH-20 resulting in 6 fractions (Figure S62). Fraction 5 was further purified by preparative HPLC to yield individual compounds—**20** (3.24 mg), **15** (9.86 mg), **16** (60.6 mg), **17** (301.3 mg) and **18** (13.8 mg).

Fraction 6 was further separated using CC with silica gel which resulted in 10 subfractions. The obtained subfractions were further purified by preparative HPLC to yield individual compounds. From subfraction 1, compounds **1** (4.14 mg) and **2** (1.72 mg) were isolated. From subfraction 7, compounds **22** (5.2 mg), **10** (2.1 mg), **13** (9.11 mg), **3** (4.21 mg), **6** (3.6 mg), **8** (4.55 mg), **4** (23.84 mg), **11** (6.15 mg) and **19** (4.44 mg) were isolated. Subfraction 2 was again separated on silica gel using the same conditions into 8 subsubfractions. From subsubfraction 8, compounds **7** (13.23 mg), **14** (17.34 mg), **5** (64.29 mg), **12** (9.51 mg), **9** (5.22 mg), and **21** (4.37 mg) were isolated (Figure S62). ^1^H-NMR, COSY, NOESY, ^13^C-NMR, HSQC, and HMBC spectra in DMSO-*d*_6_ along with HR-ESI-MS data (excluding compound **4**) for the new secondary metabolites of *H. aurantiacum* (**1**–**9**) are presented in the Supplementary Materials (Figures S1–S61).

### 4.4. Human Platelet Preparation

Human platelets were prepared according to a previous description with minor modifications [61]. Blood was obtained from healthy adult volunteers. Our studies with human platelets were approved by the Independent Ethics Committee at Sechenov Institute of Evolutionary Physiology and Biochemistry of the RAS, Russia (protocol # 03–02 from 28 February 2024), in accordance with our institutional guidelines and the Declaration of Helsinki. Informed consent was obtained from all participants. Blood was collected into ACD solution (12 mM citric acid, 15 mM sodium citrate, 25 mM D-glucose, final concentrations) with EGTA (2 mM, final concentration) and was centrifuged at 300× *g* (Centrifuge ELMI CM-6M, ELMI, Riga, Latvia) for 8 min at room temperature (RT) to obtain platelet-rich plasma (PRP). PRP was diluted (1:1) with CGS buffer (120 mM sodium chloride, 12.9 mM trisodium citrate, 10 mM D-glucose, pH 6.5) then centrifuged for 4 min at 460× *g* (Centrifuge Eppendorf 5415C, Eppendorf SE, Hamburg, Germany). The pelleted platelets were resuspended in CGS buffer, centrifuged for 4 min at 460× *g* and the platelet pellet was resuspended in HEPES buffer (150 mM sodium chloride, 5 mM potassium chloride, 1 mM magnesium chloride, 5 mM D-glucose, 10 mM HEPES, pH 7.4). Calcium chloride (1 mM, final concentration) was added before platelet stimulation. Washed platelets (WP) were used for experiments after 15 min rest at 37 °C.

### 4.5. Flow Cytometry Analysis of Platelet αIIbβ3 Integrin Activation

Analysis of platelet αIIbβ3 integrin activation was performed using the CytoFLEX flow cytometer (Beckman Coulter, Inc., Brea, CA, USA). A total of 15,000 events were acquired for each sample. CytExpert Acquisition and Analysis Software Version 2.4 (Beckman Coulter, Inc., Brea, CA, USA) was used for the data analysis.

For the measurement of platelet αIIbβ3 integrin activation fibrinogen-Alexa-Fluor 647 (15 μg/mL, final concentration) was added to WP. Then platelets were incubated with the tested compounds (90 μM) at 37 °C for 30 min. Subsequent platelet activation was induced by the addition of thrombin (200 mU/mL), then the samples were incubated at 37 °C for 3 min. The reaction was stopped by the dilution with phosphate-buffered saline (PBS, 1:10). Since all isolated compounds were dissolved in DMSO, DMSO was added in corresponding concentrations to all negative and positive (with thrombin) control samples.

### 4.6. Data Analysis

Platelet experiments were performed on platelets collected from at least four different donors. Data are presented as mean ± standard deviation (SD). GraphPad Prism 9 (GraphPad Software, San Diego, CA, USA) was used for data analysis. Significance of the differences between groups was evaluated using Mann–Whitney U-test; *p* < 0.05 was regarded statistically significant.

## 5. Conclusions

Limitations of existing antiplatelet drugs highlight the importance of discovering new therapeutic agents. Given the significant contribution of natural products and their derivatives to the tapestry of modern drugs, fungi, along with plants, are recognised as valuable sources of biologically active compounds. In this study, we isolated twenty-two secondary metabolites from the tooth fungus *Hydnellum aurantiacum* (Batsch) P. Karst., including two new kavalactones, seven new *p*-terphenyl derivatives together with thirteen known *p*-terphenyls. Structural elucidation was performed using spectroscopic methods along with comparison to literature data. The compounds were found to possess various effects on thrombin-induced platelet activation. The majority of them exhibited inhibitory activity, ranging from moderate to pronounced, with the strongest effects observed for 2(2′)-O-benzoyl derivatives of atromentin (**4**, **5**) or leucoatromentin (**7**). These findings highlight the potential of *p*-terphenyls for novel antithrombotic agents’ development.

## Figures and Tables

**Table 1:** ^1^H and ^13^C NMR spectroscopic data for **1**.

Position	δH, (*J* in Hz)	δC
2	-	167.75
3	-	101.00
4	-	167.50
5a	3.01 (1H, dd, *J* = 17.1, 1.8 Hz)	29.18
5b	2.78 (1H, dd, *J* = 17.1, 11.4 Hz)	
6	5.02 (1H, m)	74.83
7	6.44 (1H, dd, *J* = 16.0, 6.2 Hz)	127.51
8	6.75 (1H, d, *J* = 16.0 Hz)	132.40
9	-	136.26
10	7.38 (2H, t, *J* = 7.7)	129.20
11	7.50 (2H, m)	127.08
12	7.30 (1H, m)	128.63
3-Me	1.66 (3H, s)	9.31
4-OMe	3.82 (3H, s)	56.16

**Table 2:** ^1^H and ^13^C NMR spectroscopic data for **2**.

Position	δH, (*J* in Hz)	δC
2	7.56 (1H, s)	160.04
3	-	113.38
4	-	192.09
5a	2.70 (1H, dd, *J* = 17.3, 13.4)	41.56
5b	2.56 (1H, dd, *J* = 17.3, 3.7)	
6	5.13 (1H, m)	79.36
7	6.46 (1H, dd, *J* = 16.0, 6.2 Hz)	126.97
8	6.75 (1H, d, *J* = 16.0 Hz)	132.63
9	-	136.23
10,14	7.37 (2H, m)	129.20
11,13	7.49 (2H, m)	127.08
12	7.30 (1H, m)	128.63
3-Me	1.66 (3H, s)	9.31

**Table 3:** ^1^H and ^13^C NMR spectroscopic data for **3**.

Position	δH, (*J* in Hz)	δC
1	-	183.47
2	-	155.07
3	-	126.58
4	-	184.02
5	-	152.01
6	-	118.07
1′	-	132.19
2′,6′	7.17 (2H, d, *J* = 8.5)	132.19
3′,5′	6.81 (2H, d, *J* = 8.5)	114.83
4′	-	157.86
1″	-	132.38
2″,6″	7.20 (2H, d, *J* = 8.5)	132.38
3″,5″	6.78 (2H, d, *J* = 8.5)	115.0
4″	-	157.39
2-OEt	1.12 (3H, t, *J* = 7.0) 4.09 (2H, q, *J* = 7.0)	15.94 69.88
5-OH	10.55 (1H, s)	-
4′-OH	9.53 (1H, s)	-
4″-OH	9.63 (1H, s)	-

**Table 4:** ^1^H and ^13^C NMR spectroscopic data for **5**.

Position	δH, (*J* in Hz)	δC
1	-	148.42
2	-	183.03
3	-	132.23
4	-	154.85
5	-	180.71
6	-	132.35
1′	-	119.43
2′,6′	7.30 (2H, d, *J* = 8.5)	132.22
3′,5′	6.81 (2H, d, *J* = 8.5)	115.40
4′	-	159.08
1″	-	120.69
2″,6″	7.19 (2H, d, *J* = 8.5)	132.36
3″,5″	6.83 (2H, d, *J* = 8.5)	115.11
4″	-	158.20
1a	-	164.24
2a	-	127.94
3a/7a	8.01 (2H, m)	130.42
4a/6a	7.58 (2H, m)	129.64
5a	7.75 (1H, m)	135.10
5-OEt	1.17 (3H, t, *J* = 7.0) 4.17 (2H, q, *J* = 7.0)	15.95 69.95
4′-OH	9.85 (1H, s)	-
4″-OH	9.71 (1H, s)	-

**Table 5:** ^1^H and ^13^C NMR spectroscopic data for **6**.

Position	δH, (*J* in Hz)	δC
1′	-	123.15
2′	-	133.53
3′	-	142.0
4′	-	123.08
5′	-	133.64
6′	-	142.0
1	-	123.54
2,6	7.14 (2H, d, *J* = 8.5)	131.56
3,5	6.70 (2H, d, *J* = 8.5)	115.30
4	-	156.96
1″	-	123.64
2″,6″	7.10 (2H, d, *J* = 8.5)	131.50
3″,5″	6.81 (2H, d, *J* = 8.5)	115.21
4″	-	157.10
1a	-	164.35
2a	-	128.85
3a/7a	7.84 (2H, m)	129.80
4a/6a	7.51 (2H, m)	129.43
5a	7.67 (1H, m)	134.41
5-OAc	1.71 (3H, s)	20.27 168.75
3′-OH/6′-OH	8.47 (2H, s)	-
4-OH	9.48 (1H, brs)	-
4″-OH	9.46 (1H, brs)	-

**Table 6:** ^1^H and ^13^C NMR spectroscopic data for **7**.

Position	δH, (*J* in Hz)	δC
1′	-	124.79
2′	-	133.7
3′	-	143.45
4′	-	124.19
5′	-	137.27
6′	-	146.31
1	-	124.18
2,6	7.17 (2H, d, *J* = 8.5)	131.33
3,5	6.67 (2H, d, *J* = 8.5)	115.06
4	-	156.72
1″	-	124.86
2″,6″	7.19 (2H, d, *J* = 8.5)	132.44
3″,5″	6.80 (2H, d, *J* = 8.5)	114.92
4″	-	156.51
1a	-	165.05
2a	-	129.61
3a/7a	7.88 (2H, m)	130.13
4a/6a	7.48 (2H, m)	133.73
5a	7.62 (1H, m)	128.95
5-OEt	0.93 (3H, t, *J* = 7.0) 3.47 (2H, q, *J* = 7.0)	15.48 68.65
3′-OH	8.34 (1H, s)	-
6′-OH	8.20 (1H, s)	-
4-OH/4″-OH	9.33 (1H, brs)	-

**Table 7:** ^1^H and ^13^C NMR spectroscopic data for **8**.

Position	δH, (*J* in Hz)	δC
1′	-	123.06
2′	-	128.7
3′	-	142.02
4′	-	123.37
5′	-	128.8
6′	-	142.04
1	-	123.83
2	6.79 (1H, d, *J* = 1.5)	118.13
3	-	145.05
4	-	145.05
5	6.66 (1H, d, *J* = 8.2)	115.60
6	6.61 (1H, dd, *J* = 8.2, 1.5)	121.50
1″	-	123.55
2″,6″	7.17 (2H, d, *J* = 8.5)	131.60
3″,5″	6.70 (2H, d, *J* = 8.5)	115.24
4″	-	157.01
1a	-	164.54
2a	-	128.7
3a/7a	7.69 (2H, m)	129.64
4a/6a	7.33 (2H, m)	134.13
5a	7.51 (1H, m)	129.12
3′-OH	8.50 (1H, s)	-
6′-OH	8.51 (1H, s)	-
3-OH	8.80 (1H, s)	-
4-OH	8.87 (1H, s)	-
4″-OH	9.36 (1H, s)	-

**Table 8:** ^1^H and ^13^C NMR spectroscopic data for **9**.

Position	δH, (*J* in Hz)	δC
1′	-	119.56
2′	-	145.9
3′	-	136.2
4′	-	127.06
5′	-	133.9
6′	-	130.8
1	-	112.36
2	-	151.19
3	7.13 (1H, s)	99.32
4	-	148.52
5	-	143.93
6	7.01 (1H, s)	106.56
1″	-	121.73
2″,6″	7.23 (2H, d, *J* = 8.5)	131.30
3″,5″	6.67 (2H, d, *J* = 8.5)	115.61
4″	-	157.65
1a	-	164.04
2a	-	128.06
3a/7a	7.82 (2H, m)	129.93
4a/6a	7.42 (2H, m)	129.40
5a	7.60 (1H, m)	134.70
1b	-	164.04
2b	-	128.06
3b/7b	8.10 (2H, m)	130.46
4b/6b	7.60 (2H, m)	129.76
5b	7.77 (1H, m)	135.08
1c	-	163.71
2c	-	128.06
3c/7c	8.07 (2H, m)	130.35
4c/6c	7.56 (2H, m)	129.66
5c	7.73 (1H, m)	135.08
4-OH	9.79 (1H, s)	-
5-OH	9.33 (1H, s)	-
4″-OH	9.49 (1H, s)	-

## Data Availability

The data underlying this article will be shared at a reasonable request to the corresponding author.

## Supplementary Materials

The following supporting information can be downloaded at: https://www.mdpi.com/article/10.3390/molecules31122175/s1, Supplementary Figures S1–S6: ^1^H NMR, COSY, NOESY, C^13^ NMR, HSQC, HMBC spectra of compound **1** in DMSO-*d*_6_; Supplementary Figures S7–S12: ^1^H NMR, COSY, NOESY, C^13^ NMR, HSQC, HMBC spectra of compound **2** in DMSO-*d*_6_; Supplementary Figures S13–S17: ^1^H NMR, COSY, NOESY, C^13^ NMR, HMBC spectra of compound **3** in DMSO-*d*_6_; Supplementary Figures S18–S23: ^1^H NMR, COSY, NOESY, C^13^ NMR, HSQC, HMBC spectra of compound **4** in DMSO-*d*_6_; Supplementary Figures S24–S29: ^1^H NMR, COSY, NOESY, C^13^ NMR, HSQC, HMBC spectra of compound **5** in DMSO-*d*_6_; Supplementary Figures S30–S35: ^1^H NMR, COSY, NOESY, C^13^ NMR, HSQC, HMBC spectra of compound **6** in DMSO-*d*_6_; Supplementary Figures S36–S41: ^1^H NMR, COSY, NOESY, C^13^ NMR, HSQC, HMBC spectra of compound **7** in DMSO-*d*_6_; Supplementary Figures S42–S47: ^1^H NMR, COSY, NOESY, C^13^ NMR, HSQC, HMBC spectra of compound **8** in DMSO-*d*_6_; Supplementary Figures S48–S53: ^1^H NMR, COSY, NOESY, C^13^ NMR, HSQC, HMBC spectra of compound **9** in DMSO-*d*_6_; Supplementary Figures S54–S61: HR-ESI-MS spectra of compounds **1**, **2**, **3**, **5**, **6**, **7**, **8**, **9**; Supplementary Figure S62: isolation flowchart of compounds **1**–**22** from *H. aurantiacum*.

## Abbreviations

The following abbreviations are used in this manuscript:

ADP	Adenosine diphosphate
ATP	Adenosine triphosphate
cAMP	Cyclic adenosine monophosphate
cGMP	Cyclic guanosine monophosphate
COSY	Correlation spectroscopy
DMSO	Dimethyl sulfoxide
ERK2	Extracellular signal-regulated kinase 2
HMBC	Heteronuclear multiple bond correlation
HR-ESI-MS	High-resolution electrospray ionisation mass spectrometry
JNK	c-Jun N-terminal kinase
NMR	Nuclear magnet resonance
NOESY	Nuclear Overhauser effect spectroscopy
p38 MAPK	p38 mitogen-activated protein kinase
UV/VIS	Ultraviolet-visible spectroscopy
VASP	Vasodilator-stimulated phosphoprotein
