# Supplementary material for: The p-Terphenyl and Kavalactone Secondary Metabolites from the Fungus Hydnellum aurantiacum: Isolation and Evaluation of Their Effects on Platelet Activation
Source: Molecules. 2026 Jun 21;31(12):2175. doi: 10.3390/molecules31122175 (PMC13304895; doi:10.3390/molecules31122175)

The *p*-Terphenyl and Kavalactone Secondary Metabolites from the Fungus *Hydnellum aurantiacum*: Isolation and Evaluation of Their Effects on Platelet Activation

Contents

Figure S1. ^{1}H NMR spectrum of compound **1** in DMSO-d_6.

Figure S2. COSY spectrum of compound **1** in DMSO-d_6.

Figure S3. NOESY spectrum of compound **1** in DMSO-d_6.

Figure S4. C^{13} NMR spectrum of compound **1** in DMSO-d_6.

Figure S5. HSQC spectrum of compound **1** in DMSO-d_6.

Figure S6. HMBC spectrum of compound **1** in DMSO-d_6.

Figure S7. ^{1}H NMR spectrum of compound **2** in DMSO-d_6.

Figure S8. COSY spectrum of compound **2** in DMSO-d_6.

Figure S9. NOESY spectrum of compound **2** in DMSO-d_6.

Figure S10. C^{13} NMR spectrum of compound **2** in DMSO-d_6.

Figure S11. HSQC spectrum of compound **2** in DMSO-d_6.

Figure S12. HMBC spectrum of compound **2** in DMSO-d_6.

Figure S13. ^{1}H NMR spectrum of compound **3** in DMSO-d_6.

Figure S14. COSY spectrum of compound **3** in DMSO-d_6.

Figure S15. NOESY spectrum of compound **3** in DMSO-d_6.

Figure S16. C^{13} NMR spectrum of compound **3** in DMSO-d_6.

Figure S17. HMBC spectrum of compound **3** in DMSO-*d*6.

Figure S18. ^{1}H NMR spectrum of compound **4** in DMSO-*d*6.

Figure S19. COSY spectrum of compound **4** in DMSO-*d*6.

Figure S20. NOESY spectrum of compound **4** in DMSO-*d*6.

Figure S21. C^{13} NMR spectrum of compound **4** in DMSO-*d*6.

Figure S22. HSQC spectrum of compound **4** in DMSO-*d*6.

Figure S23. HMBC spectrum of compound **4** in DMSO-*d*6.

Figure S24. ^{1}H NMR spectrum of compound **5** in DMSO-*d*6.

Figure S25. COSY spectrum of compound **5** in DMSO-*d*6.

Figure S26. NOESY spectrum of compound **5** in DMSO-*d*6.

Figure S27. C^{13} NMR spectrum of compound **5** in DMSO-*d*6.

Figure S28. HSQC spectrum of compound **5** in DMSO-*d*6.

Figure S29. HMBC spectrum of compound **5** in DMSO-*d*6.

Figure S30. ^{1}H NMR spectrum of compound **6** in DMSO-*d*6.

Figure S31. COSY spectrum of compound **6** in DMSO-*d*6.

Figure S32. NOESY spectrum of compound **6** in DMSO-*d*6.

Figure S33. C^{13} NMR spectrum of compound **6** in DMSO-*d*6.

Figure S34. HSQC spectrum of compound **6** in DMSO-*d*6.

Figure S35. HMBC spectrum of compound **6** in DMSO-*d*6.

Figure S36. ^{1}H NMR spectrum of compound **7** in DMSO-*d*6.

Figure S37. COSY spectrum of compound **7** in DMSO-*d*6.

Figure S38. NOESY spectrum of compound **7** in DMSO-*d*6.

Figure S39. C^{13} NMR spectrum of compound **7** in DMSO-*d*6.

Figure S40. HSQC spectrum of compound **7** in DMSO-*d*6.

Figure S41. HMBC spectrum of compound **7** in DMSO-*d*6.

Figure S42. ^{1}H NMR spectrum of compound **8** in DMSO-*d*6.

Figure S43. COSY spectrum of compound **8** in DMSO-*d*6.

Figure S44. NOESY spectrum of compound **8** in DMSO-*d*6.

Figure S45. C^{13} NMR spectrum of compound **8** in DMSO-*d*6.

Figure S46. HSQC spectrum of compound **8** in DMSO-d_6.

Figure S47. HMBC spectrum of compound **8** in DMSO-d_6.

Figure S48. ^{1}H NMR spectrum of compound **9** in DMSO-d_6.

Figure S49. COSY spectrum of compound **9** in DMSO-d_6.

Figure S50. NOESY spectrum of compound **9** in DMSO-d_6.

Figure S51. C^{13} NMR spectrum of compound **9** in DMSO-d_6.

Figure S52. HSQC spectrum of compound **9** in DMSO-d_6.

Figure S53. HMBC spectrum of compound **9** in DMSO-d_6.

Figure S54. HR-ESI-MS spectrum of compound **1**.

Figure S55. HR-ESI-MS spectrum of compound **2**.

Figure S56. HR-ESI-MS spectrum of compound **3**.

Figure S57. HR-ESI-MS spectrum of compound **5**.

Figure S58. HR-ESI-MS spectrum of compound **6**.

Figure S59. HR-ESI-MS spectrum of compound **7**.

Figure S60. HR-ESI-MS spectrum of compound **8**.

Figure S61. HR-ESI-MS spectrum of compound **9**.

Figure S62. Isolation flowchart of compounds **1**-**22** from *H. aurantiacum*.

Figure S1. ^{1}H NMR spectrum of compound **1** in DMSO-d_6.

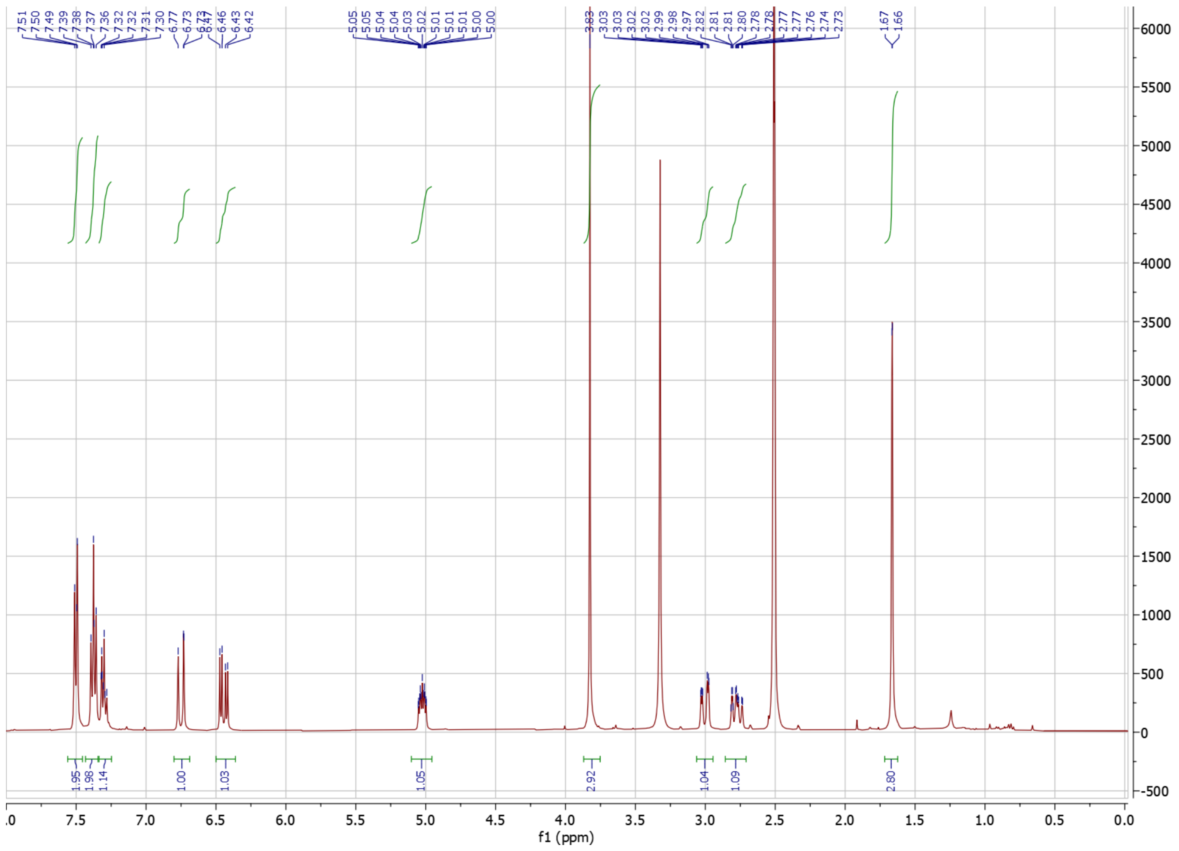

Figure S2. COSY spectrum of compound **1** in DMSO-d_6.

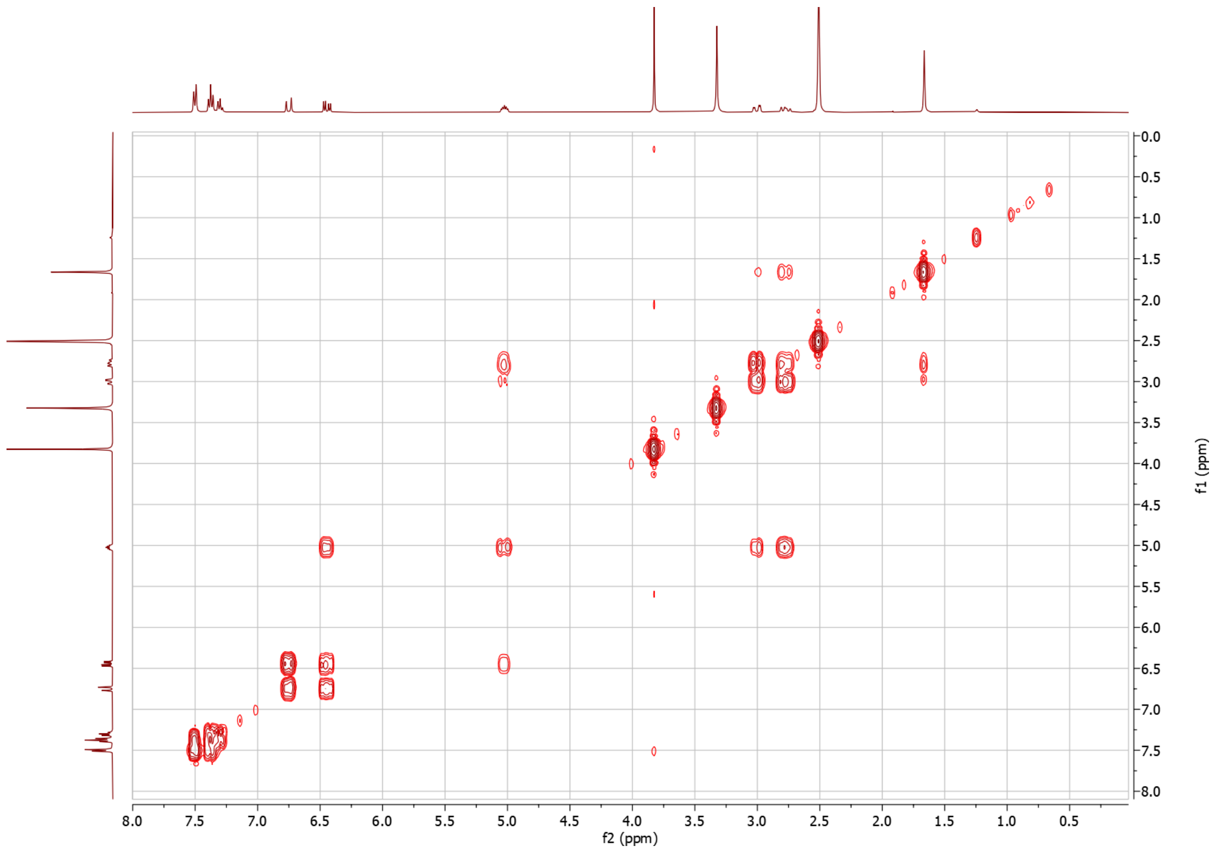

Figure S3. NOESY spectrum of compound **1** in DMSO-d_6.

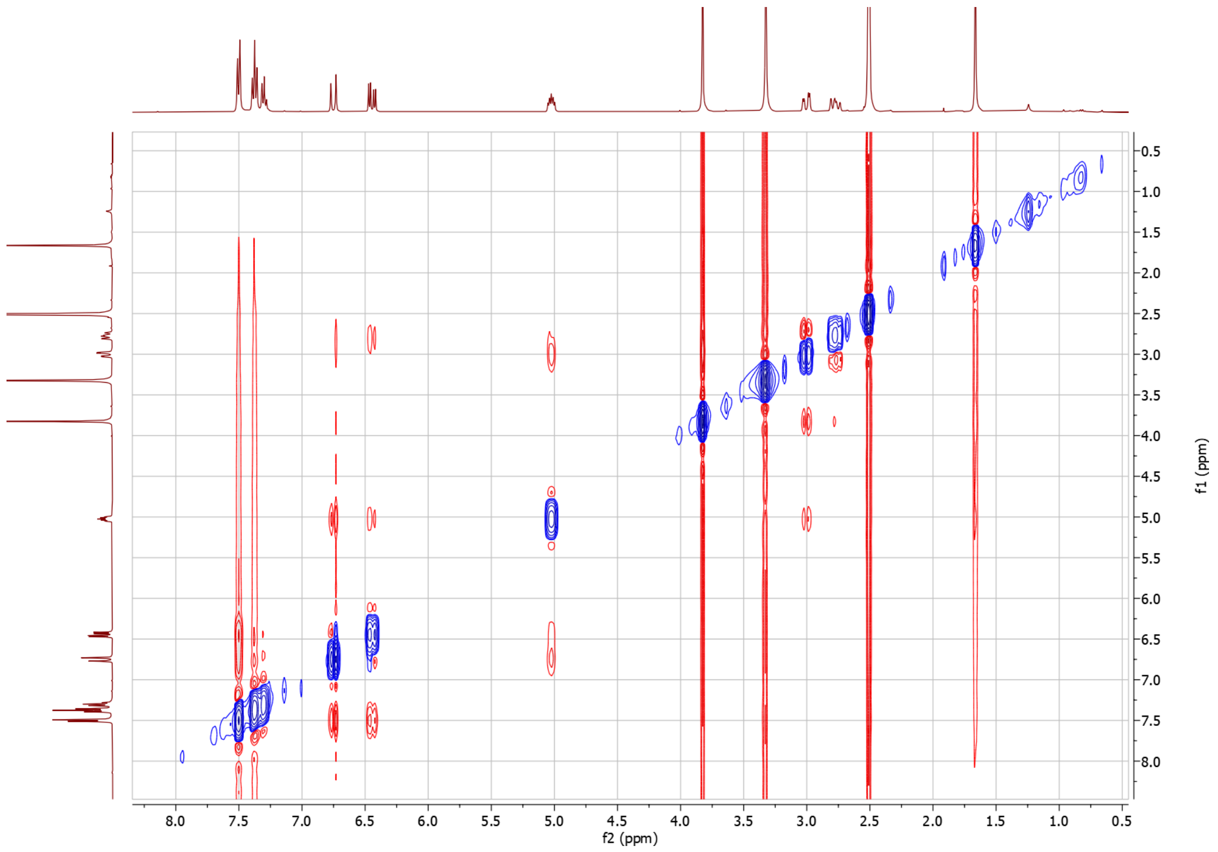

Figure S4. C^{13} NMR spectrum of compound **1** in DMSO-d_6.

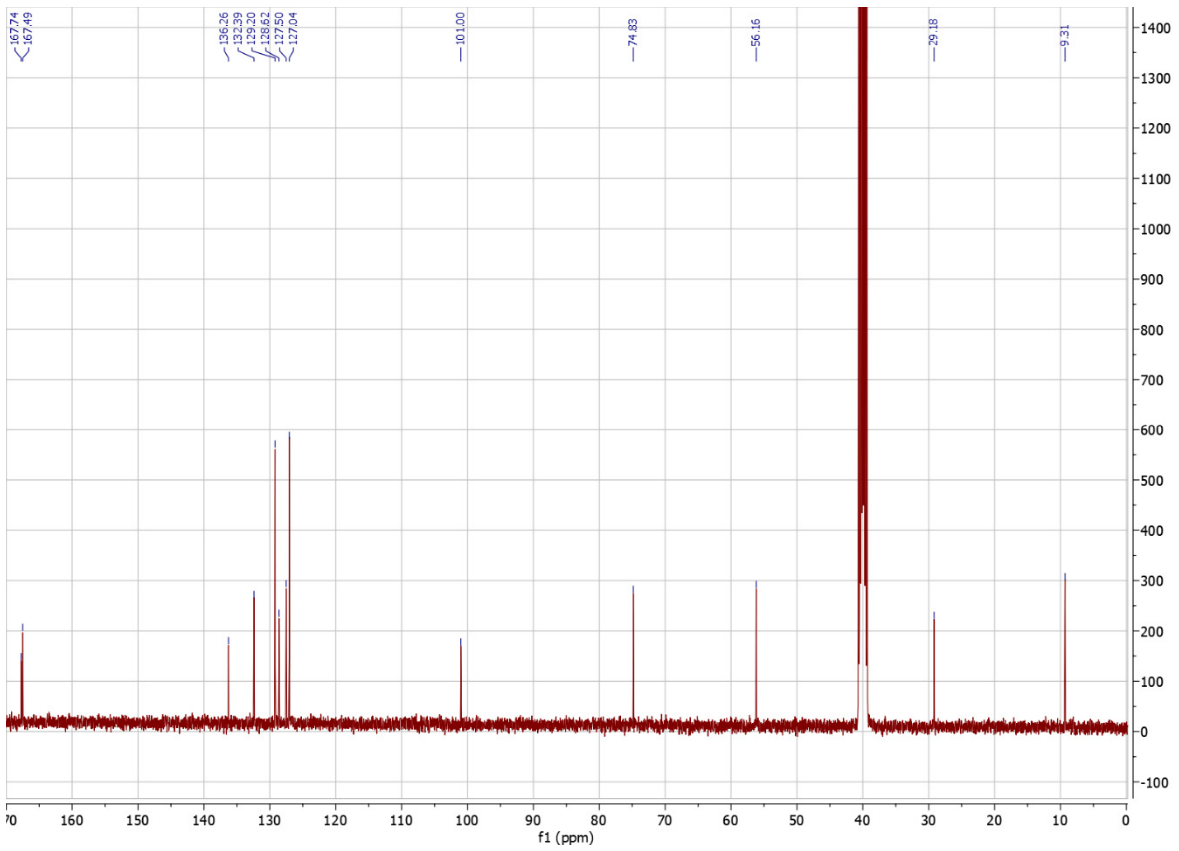

Figure S5. HSQC spectrum of compound **1** in DMSO-d_6.

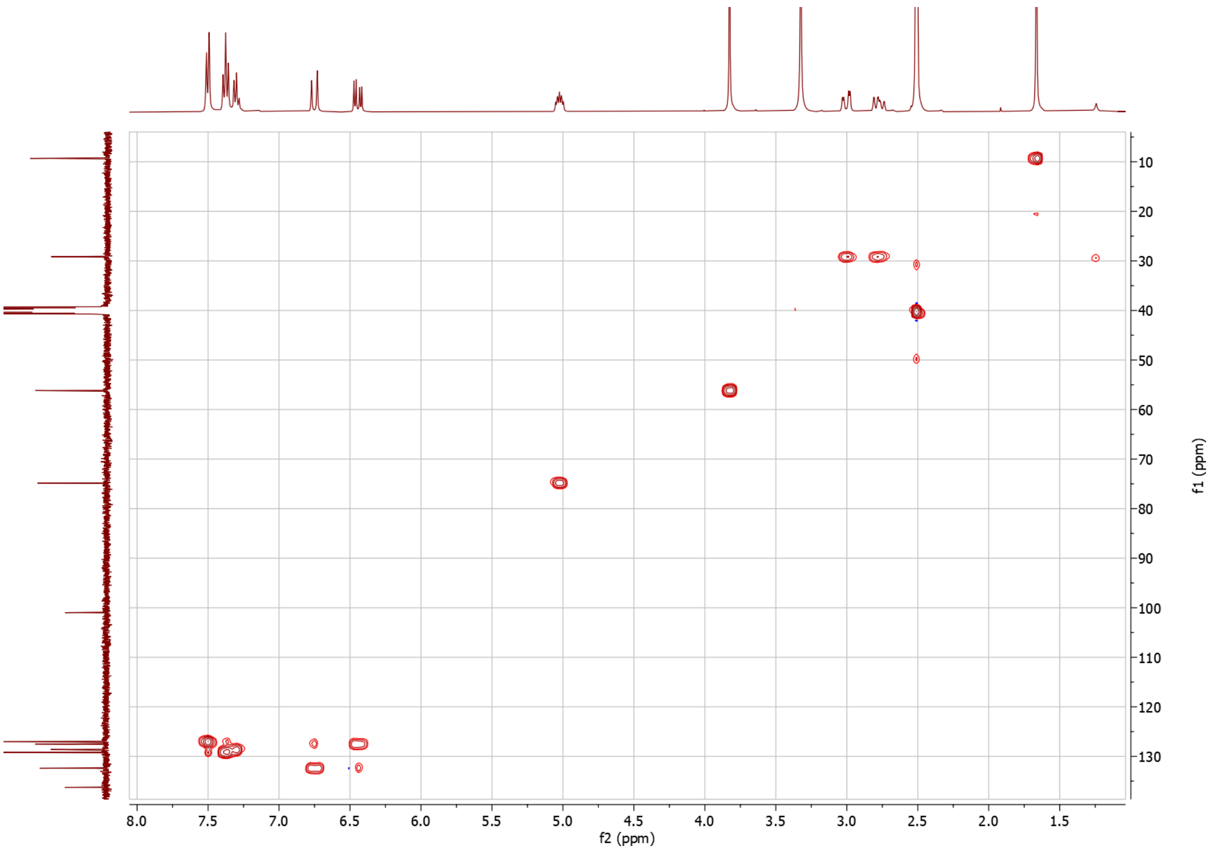

Figure S6. HMBC spectrum of compound **1** in DMSO-d_6.

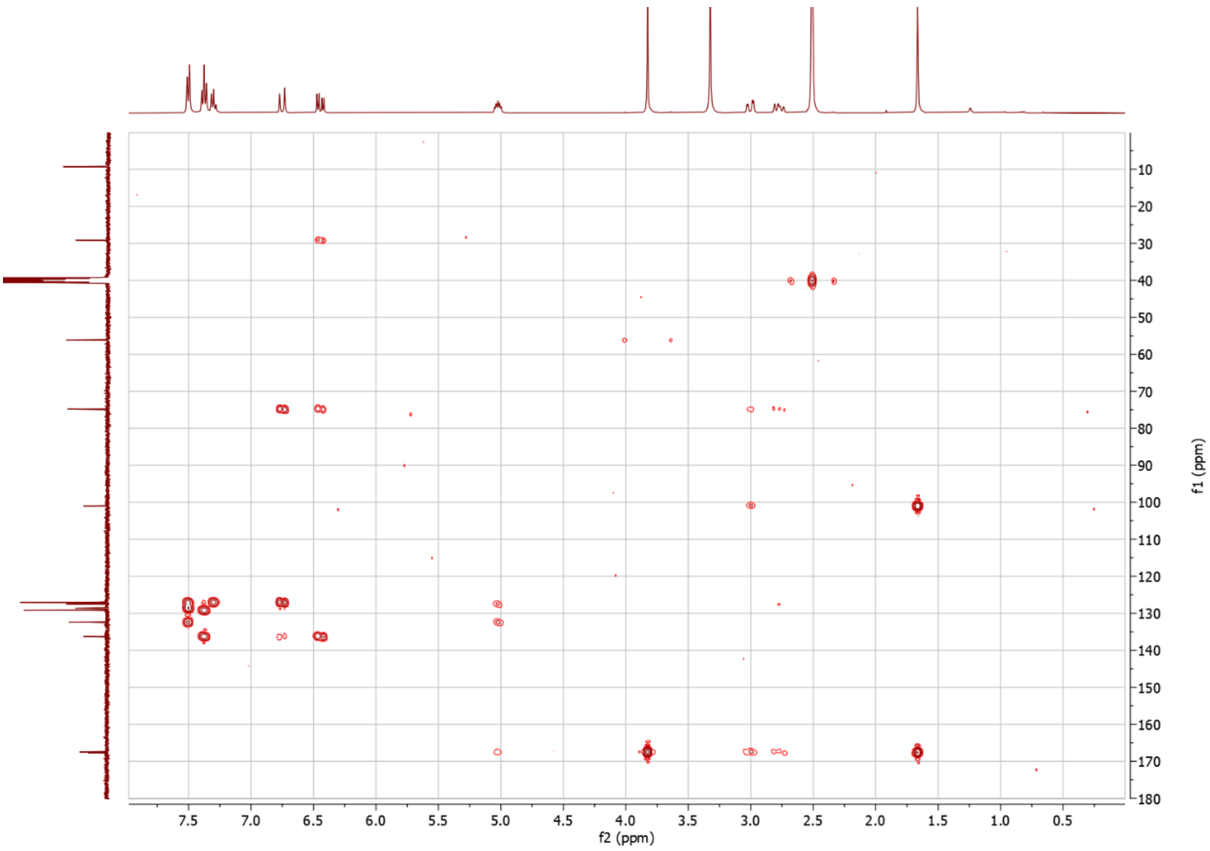

Figure S7. ^{1}H NMR spectrum of compound **2** in DMSO-d_6.

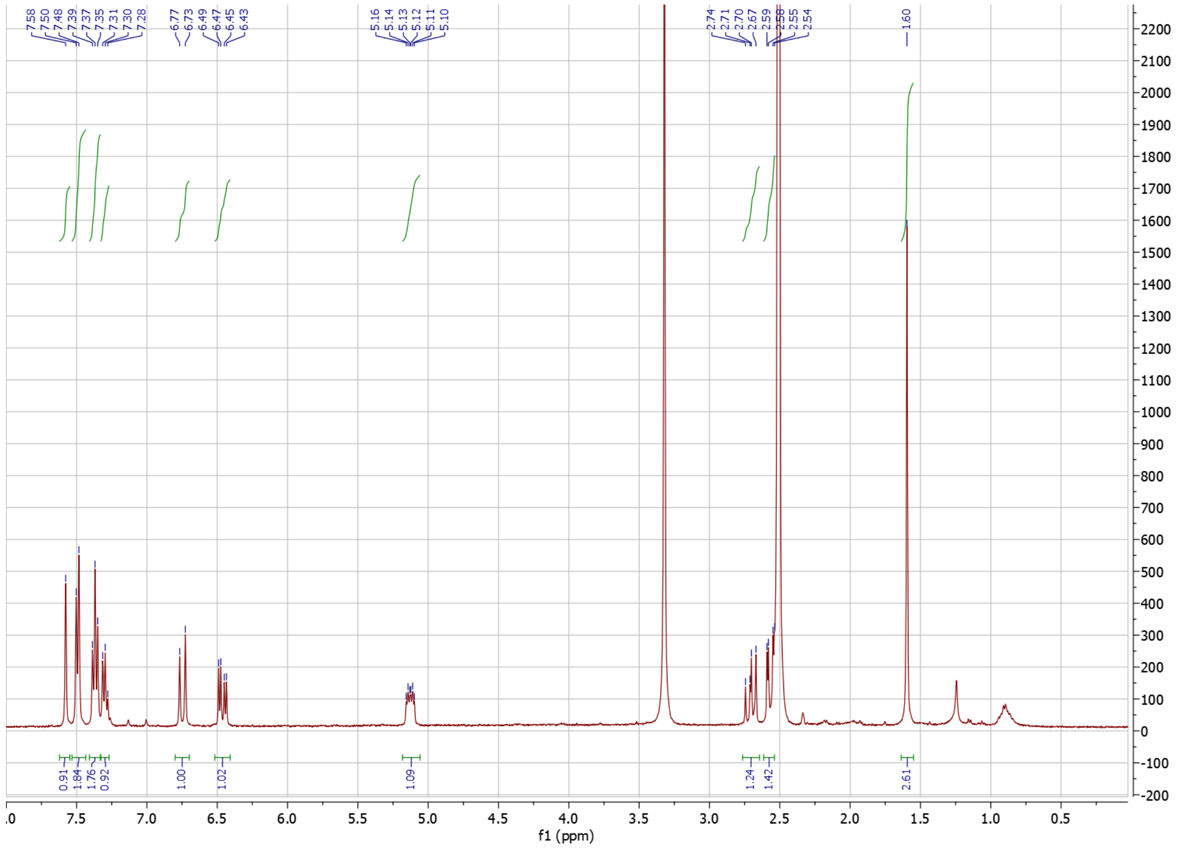

Figure S8. COSY spectrum of compound **2** in DMSO-d_6.

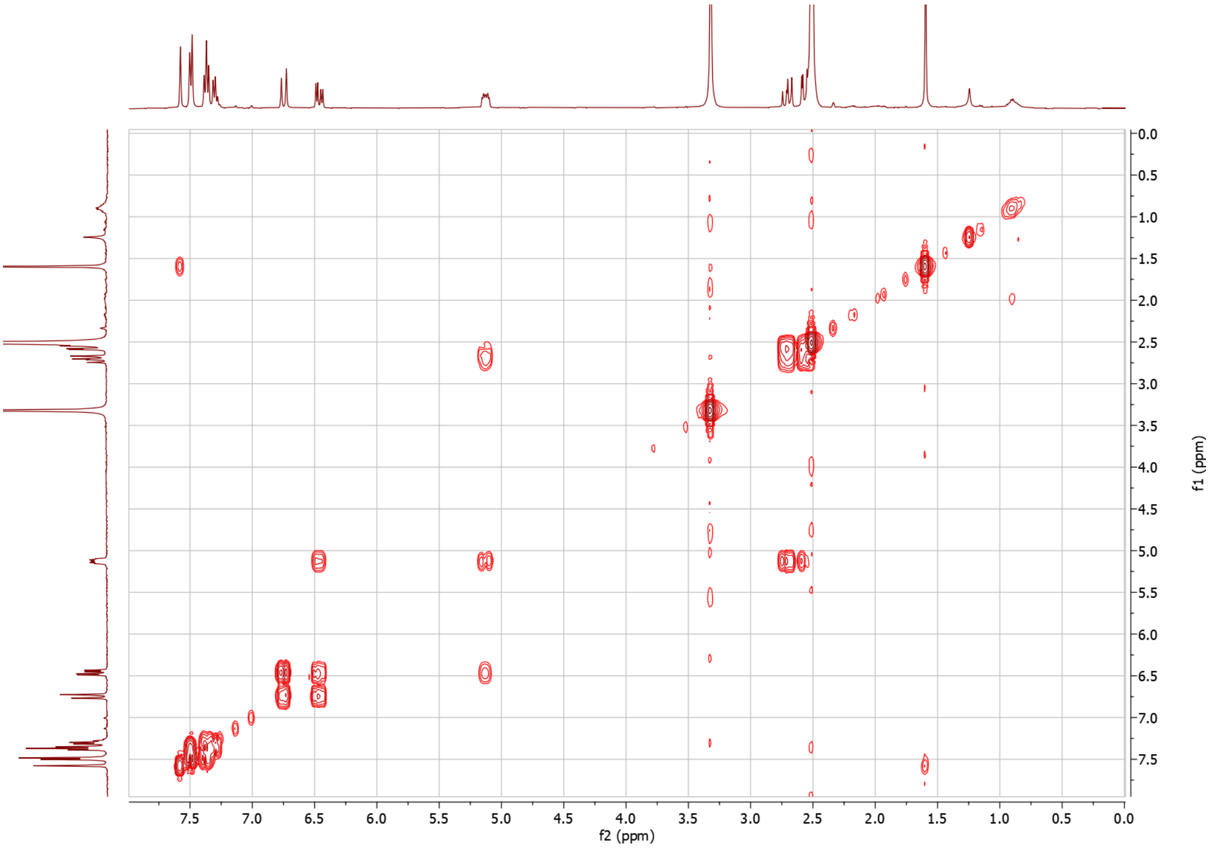

Figure S9. NOESY spectrum of compound **2** in DMSO-d_6.

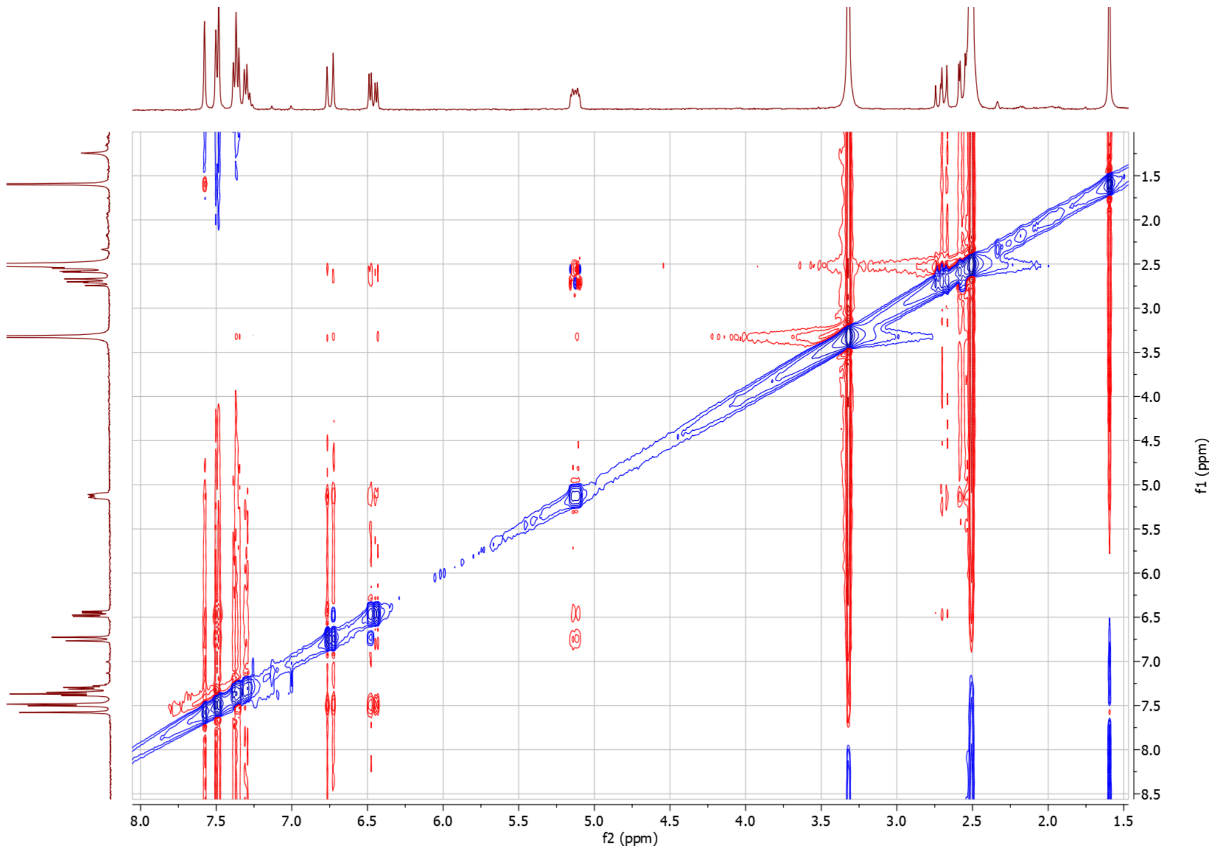

Figure S10. C[13] NMR spectrum of compound **2** in DMSO-d_6.

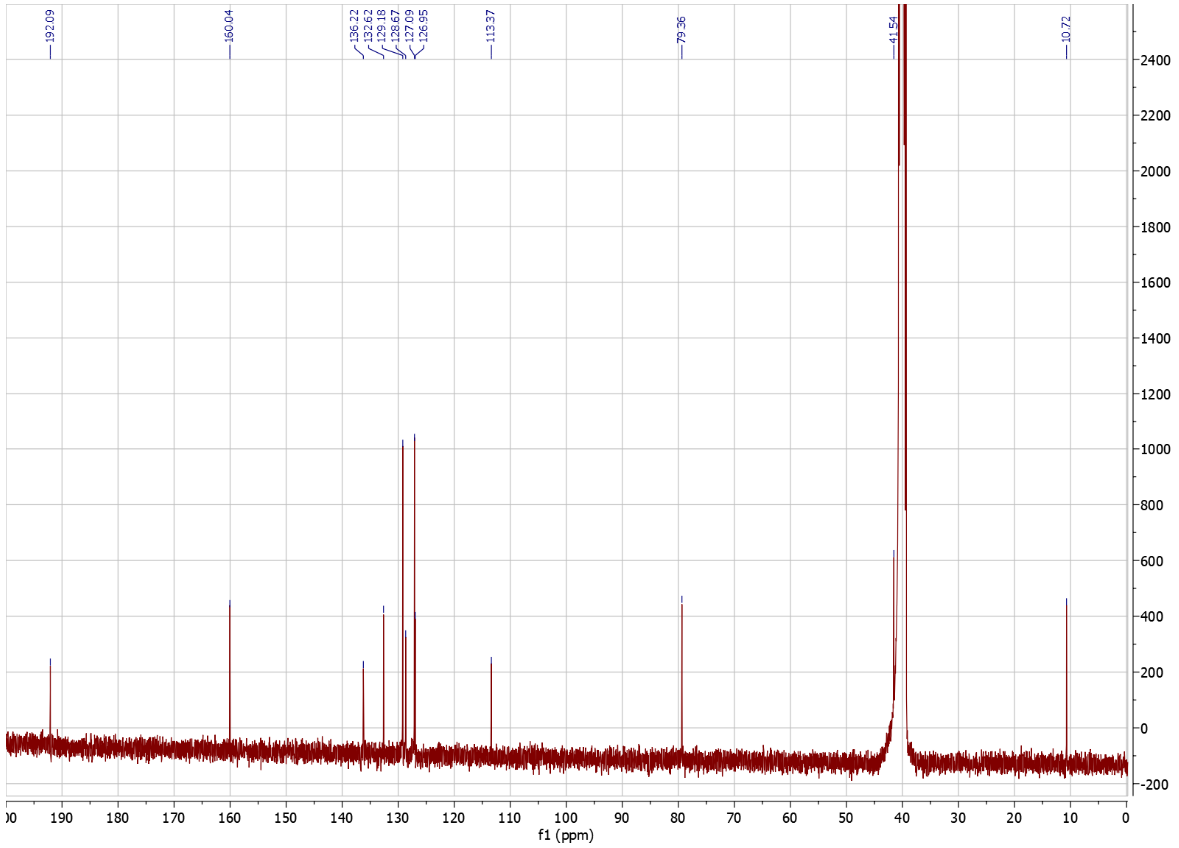

Figure S11. HSQC spectrum of compound **2** in DMSO-d_6.

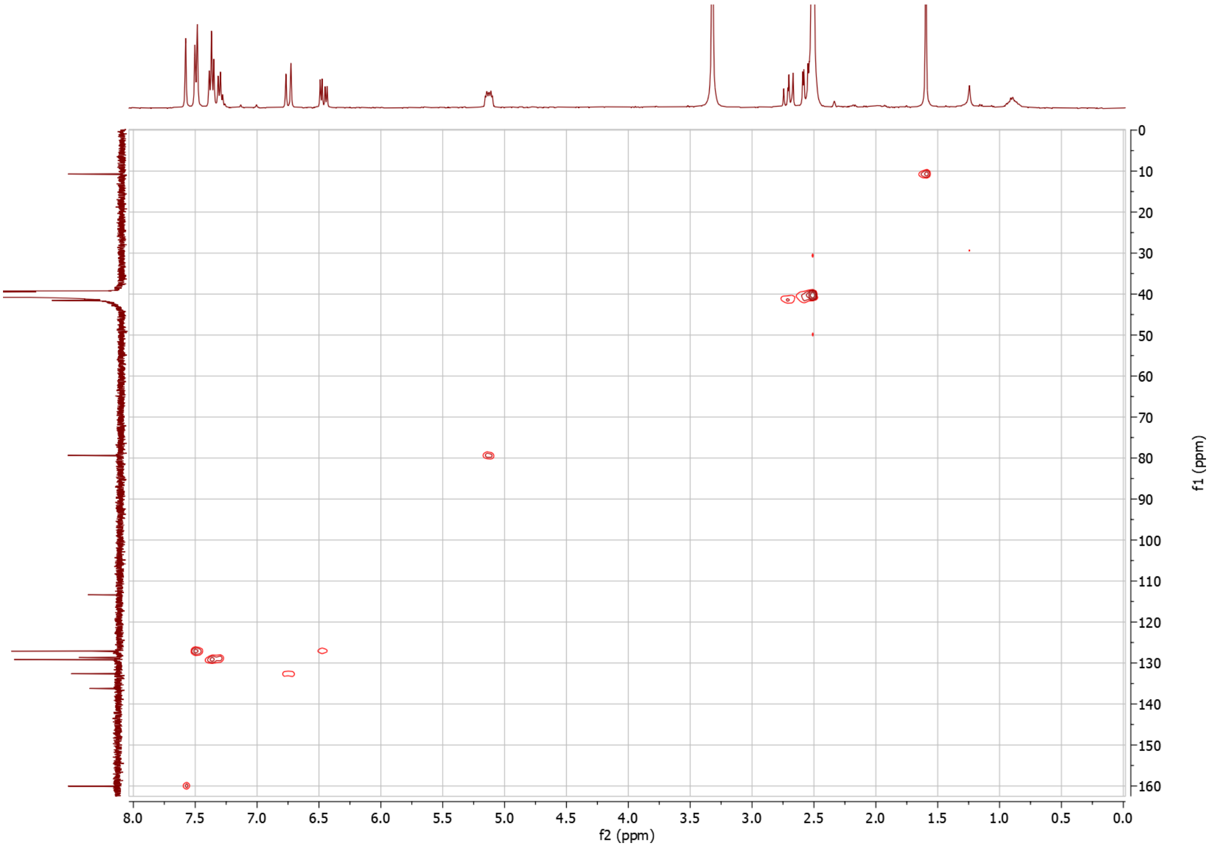

Figure S12. HMBC spectrum of compound **2** in DMSO-d_6.

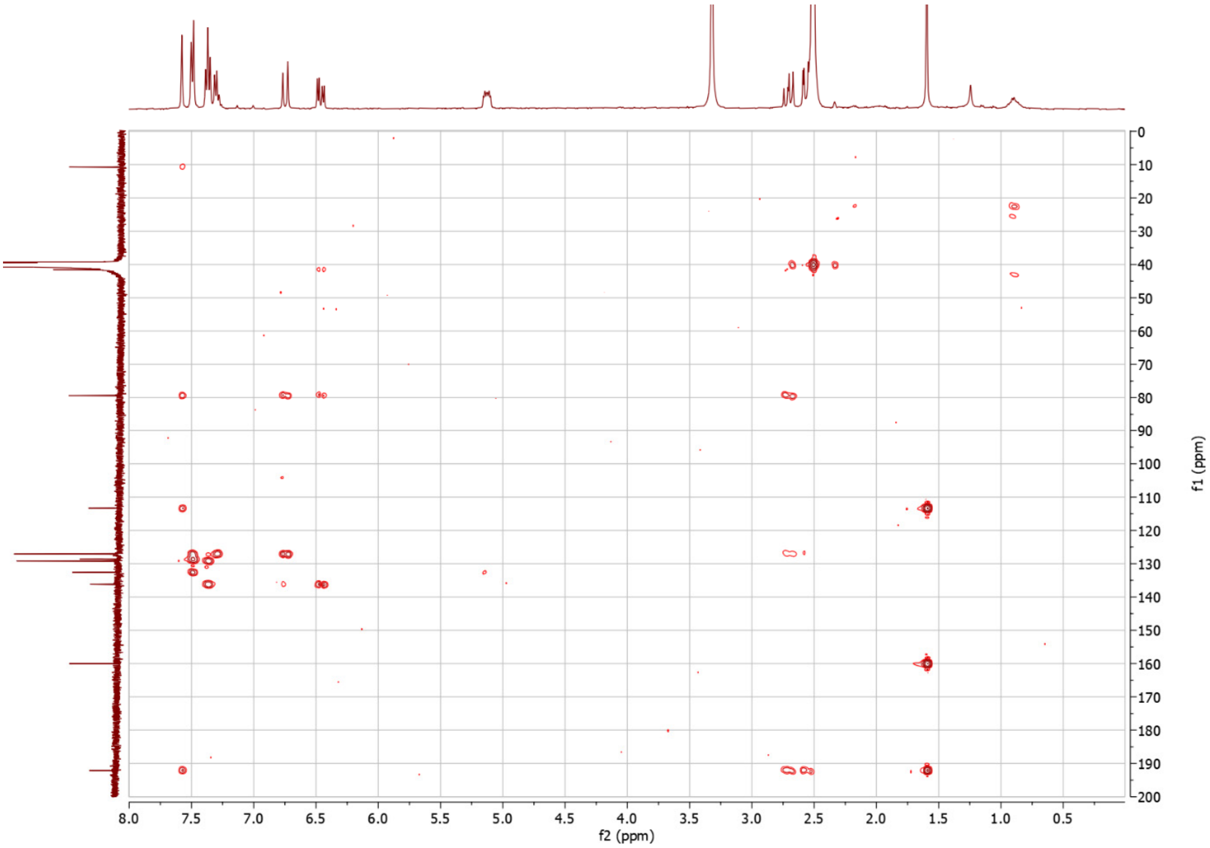

Figure S13. ^{1}H NMR spectrum of compound **3** in DMSO-d_6.

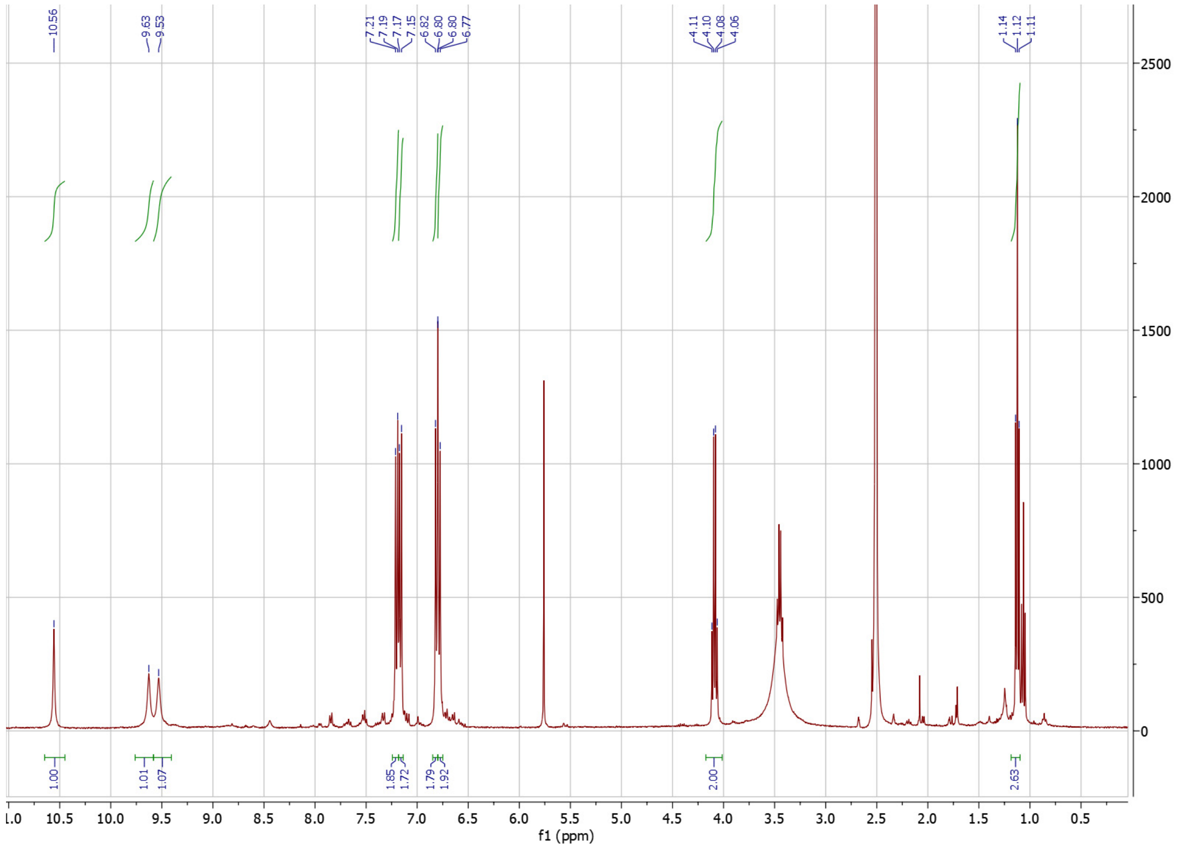

Figure S14. COSY spectrum of compound **3** in DMSO-d_6.

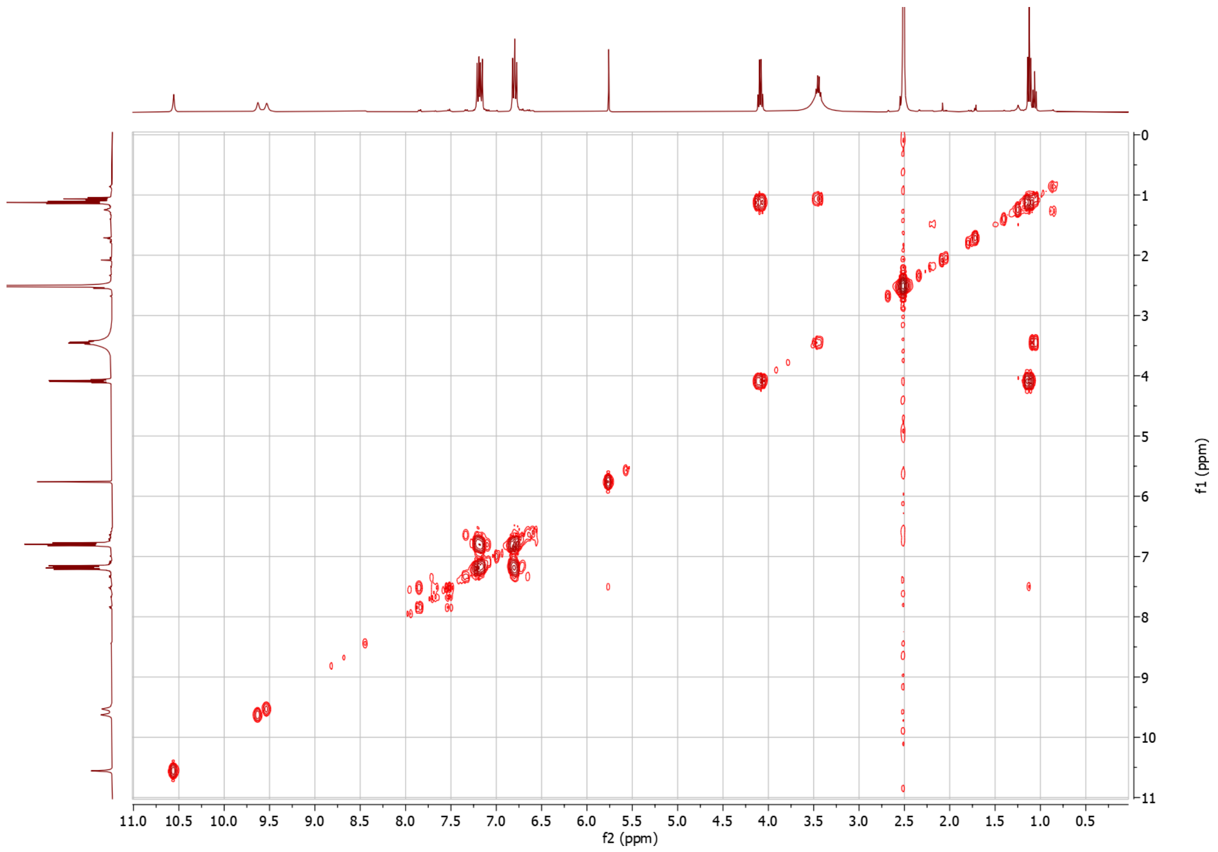

Figure S15. NOESY spectrum of compound **3** in DMSO-d_6.

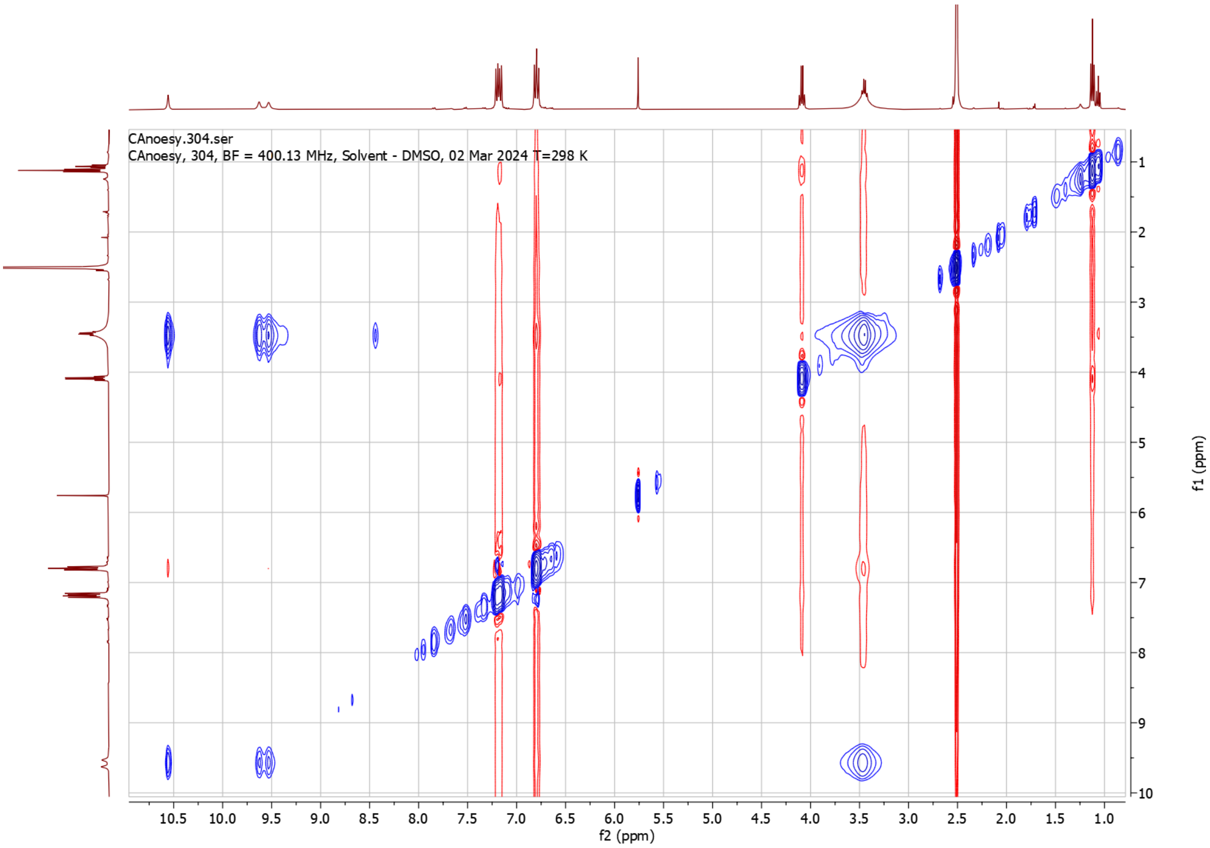

Figure S16. C[13] NMR spectrum of compound **3** in DMSO-d_6.

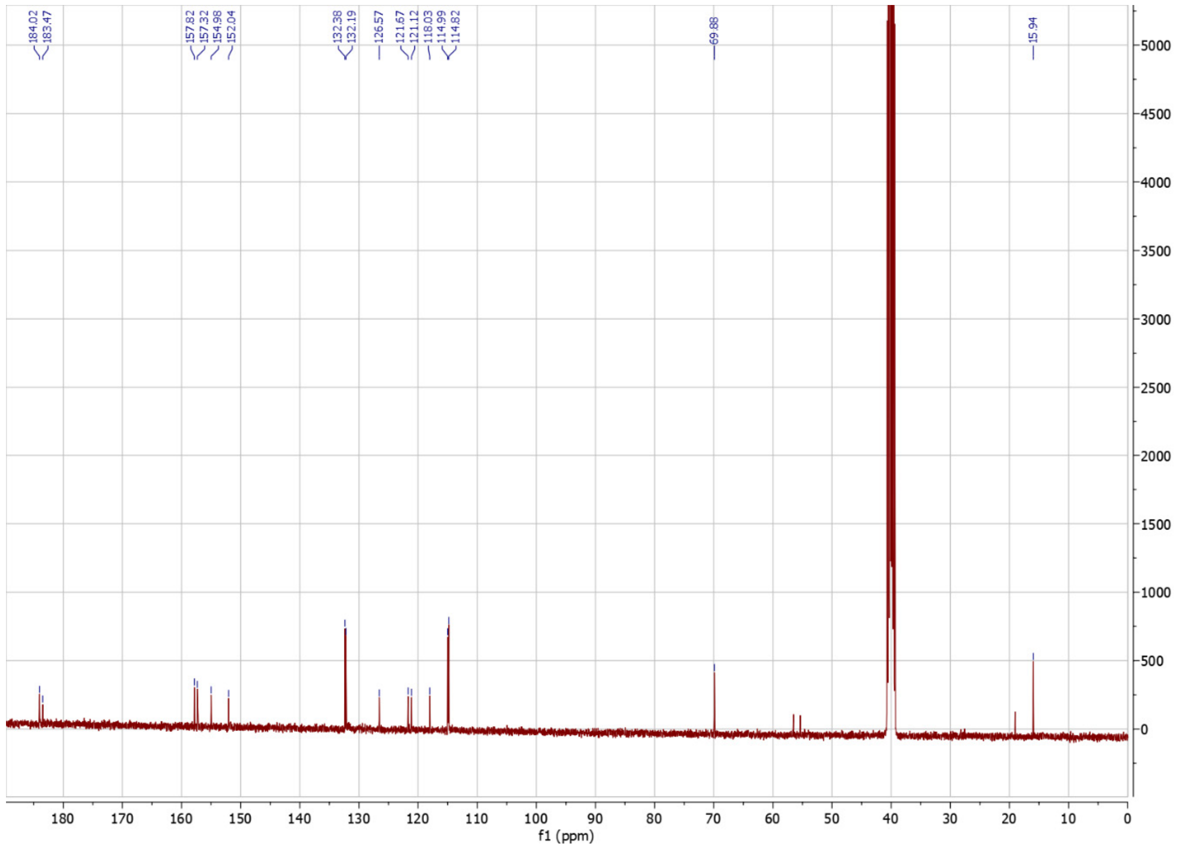

Figure S17. HMBC spectrum of compound **3** in DMSO-*d*$_6$.

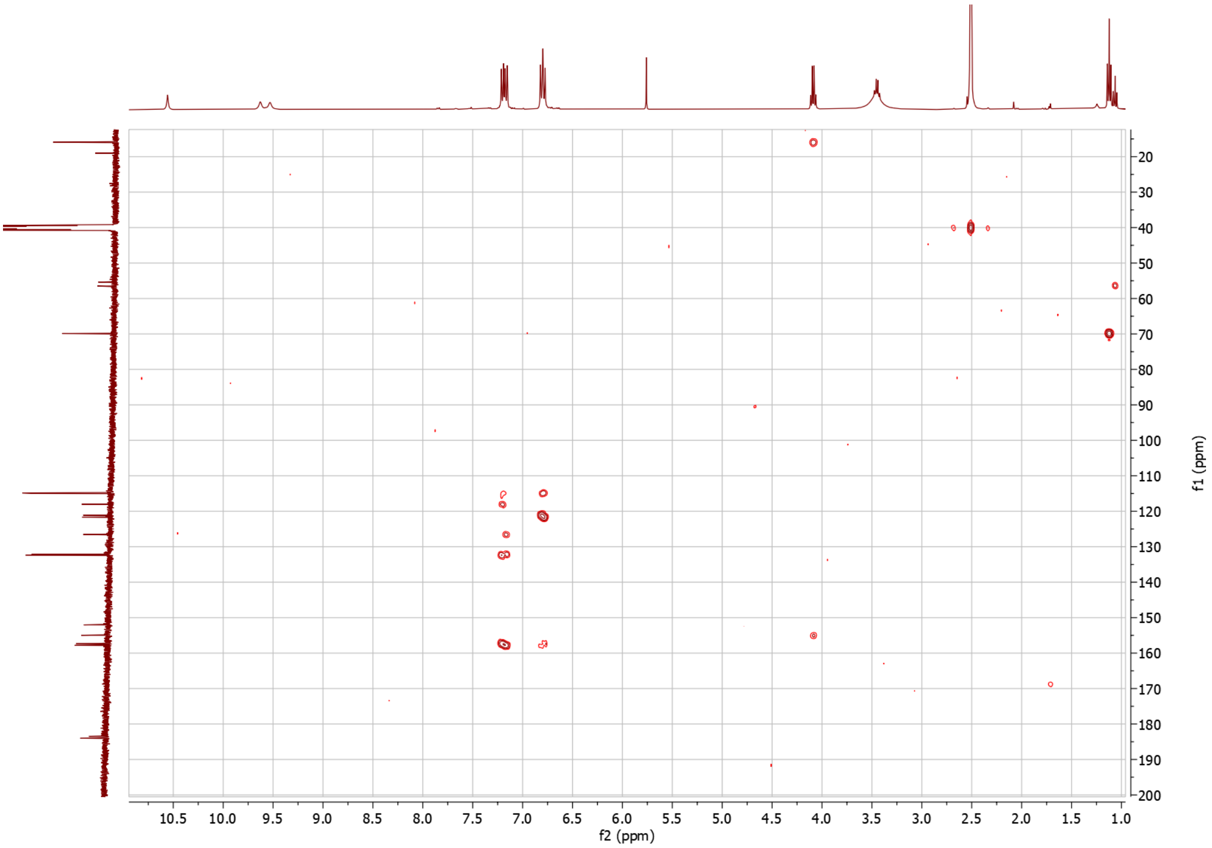

Figure S18. ^{1}H NMR spectrum of compound **4** in DMSO-d_6.

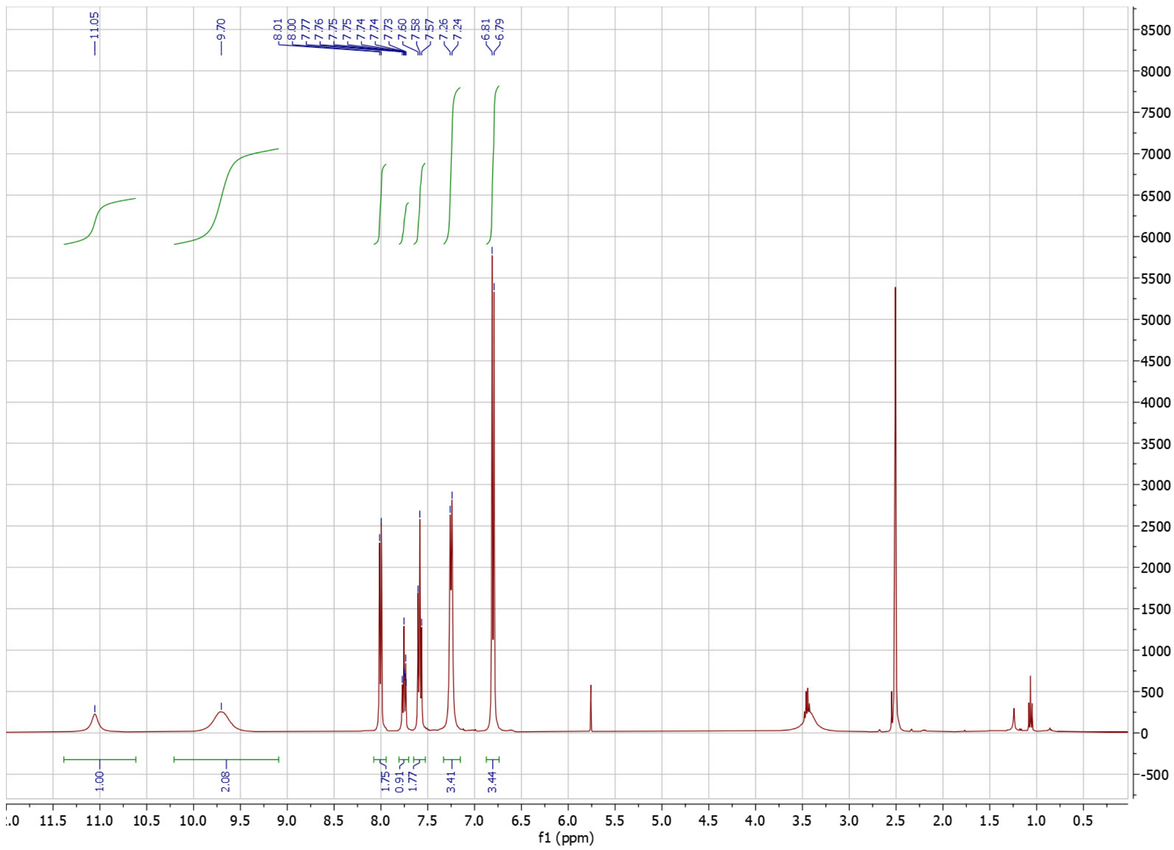

Figure S19. COSY spectrum of compound **4** in DMSO-d_6.

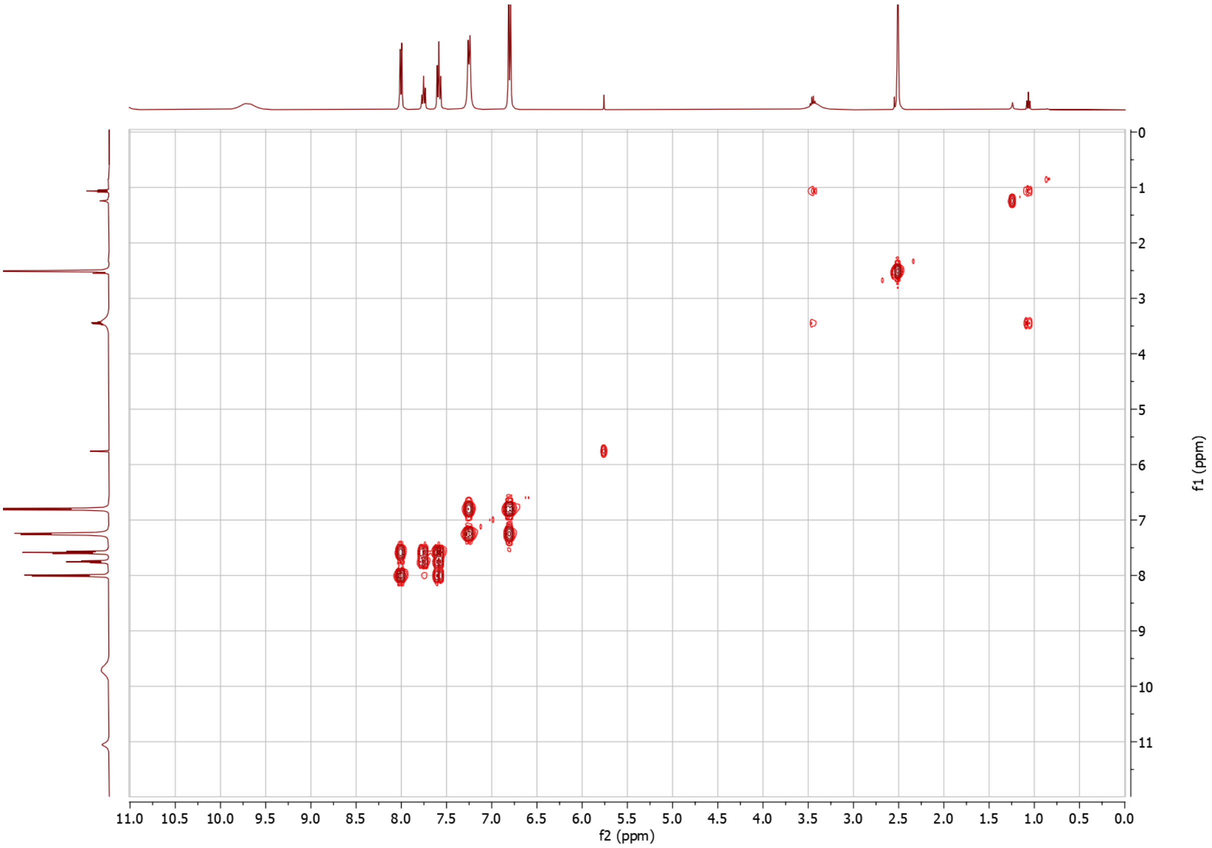

Figure S20. NOESY spectrum of compound **4** in DMSO-d_6.

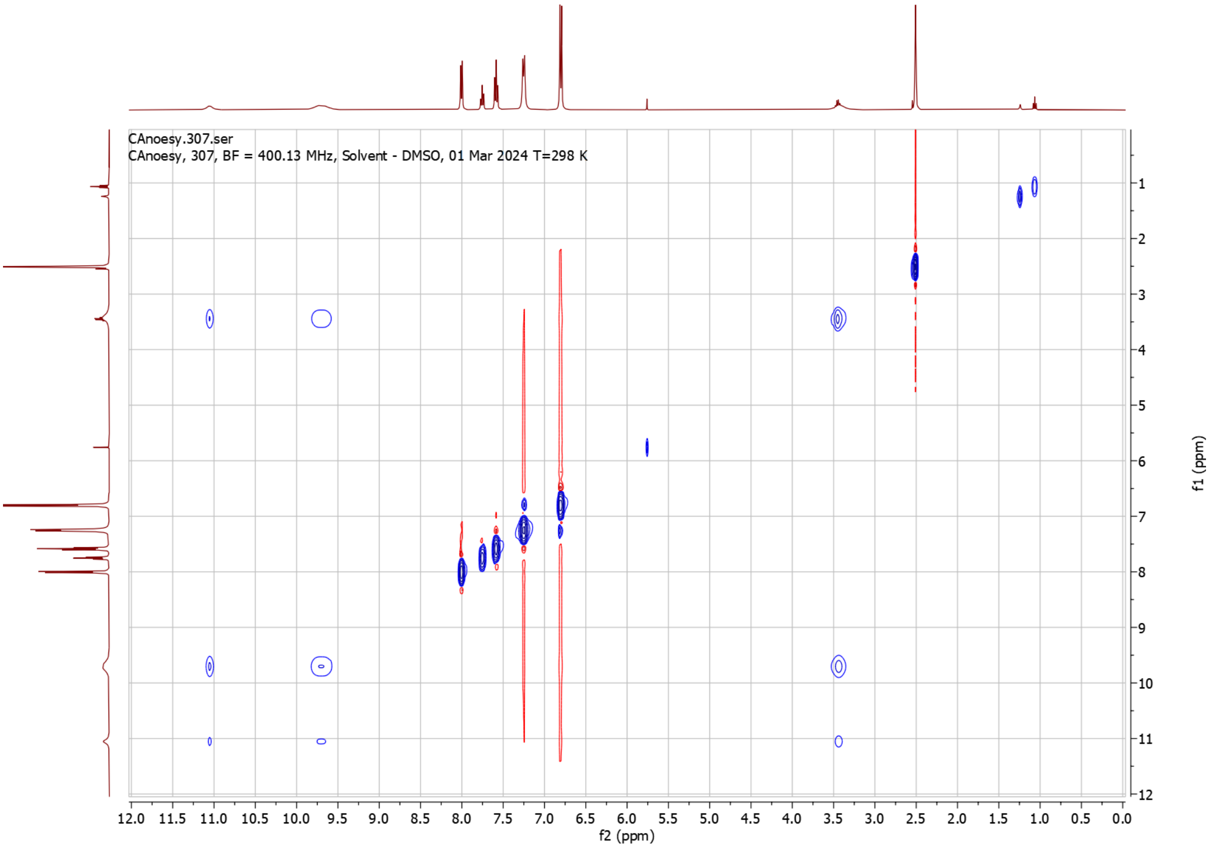

Figure S21. C^{13} NMR spectrum of compound **4** in DMSO-d_6.

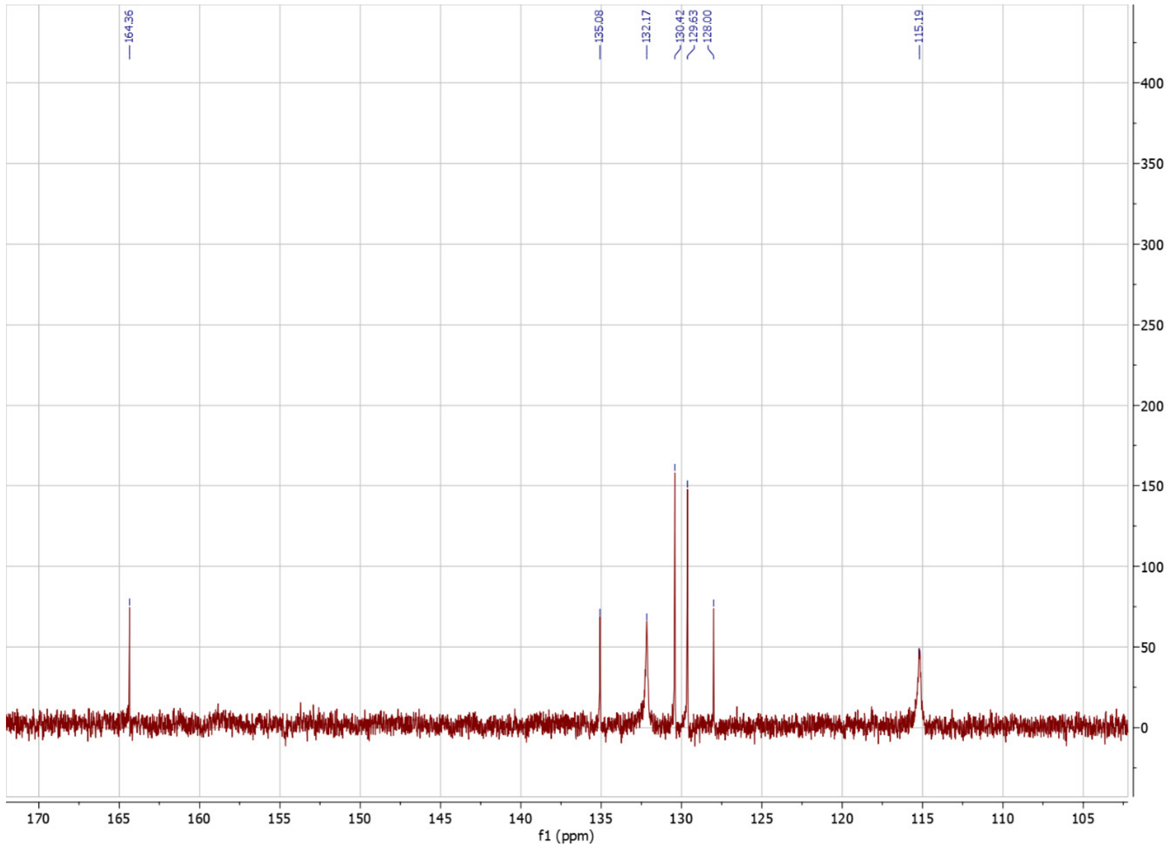

Figure S22. HSQC spectrum of compound **4** in DMSO-d_6.

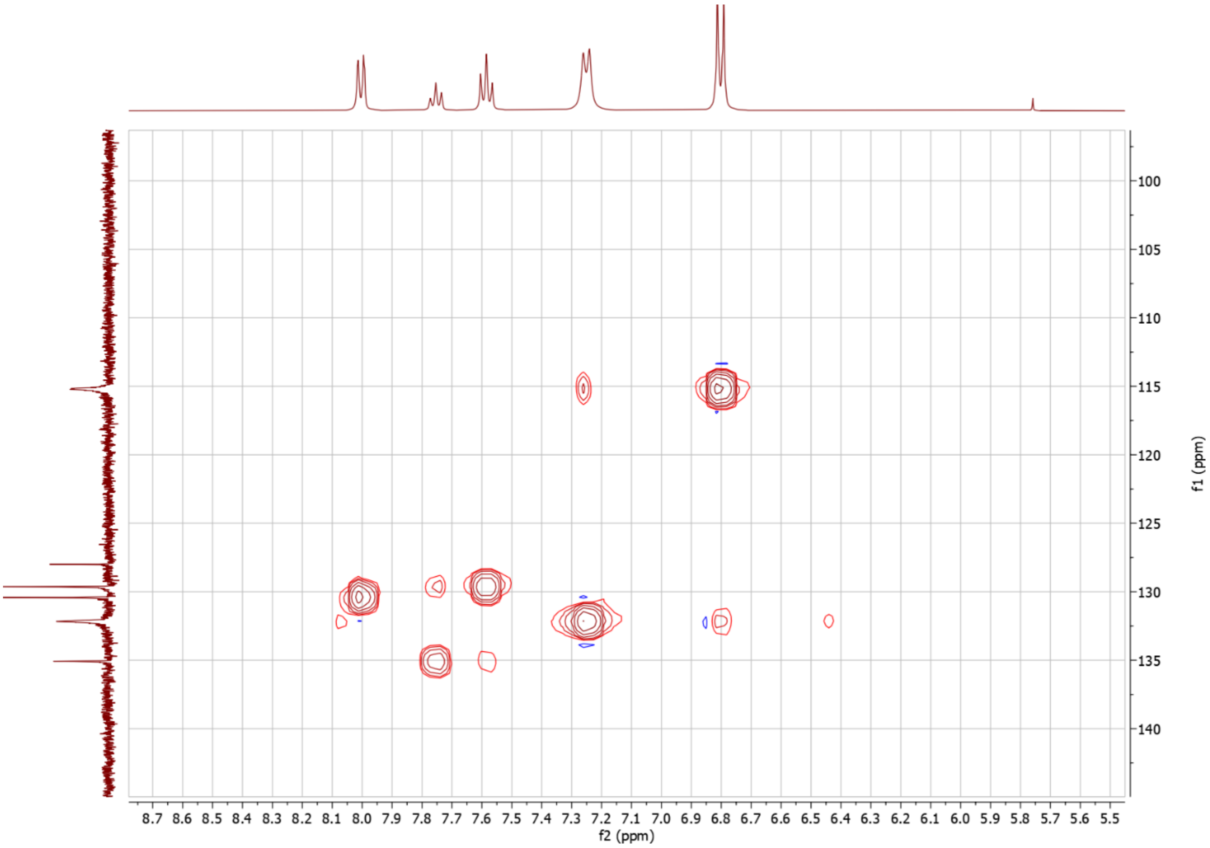

Figure S23. HMBC spectrum of compound **4** in DMSO-d_6.

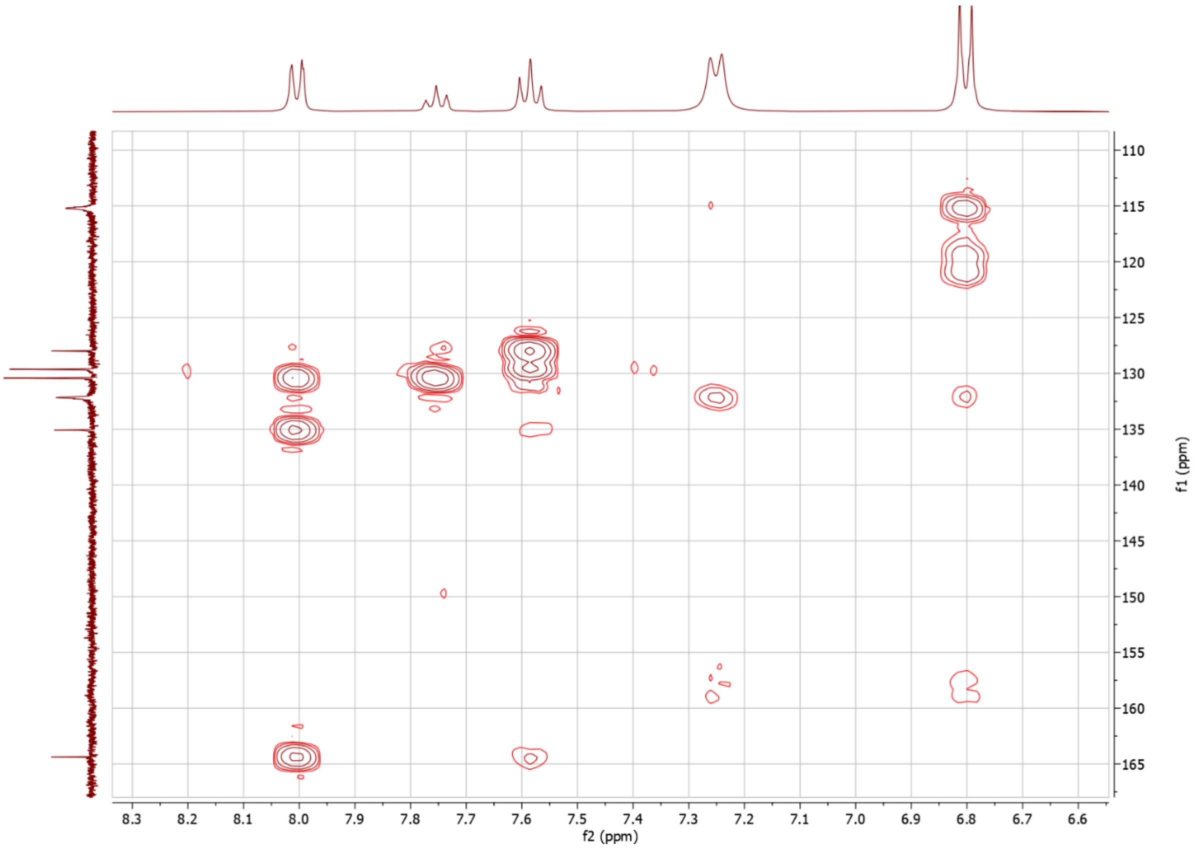

Figure S24. ^{1}H NMR spectrum of compound **5** in DMSO-d_6.

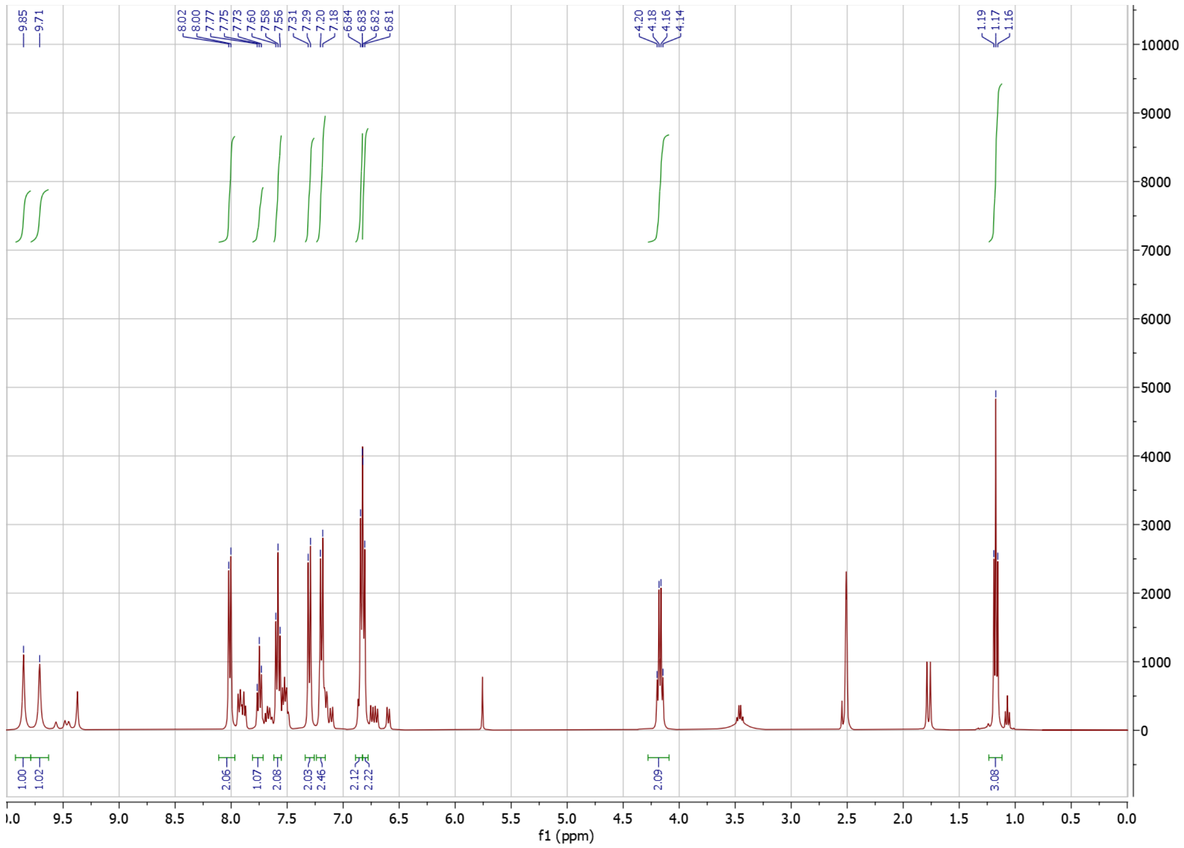

Figure S25. COSY spectrum of compound **5** in DMSO-d_6.

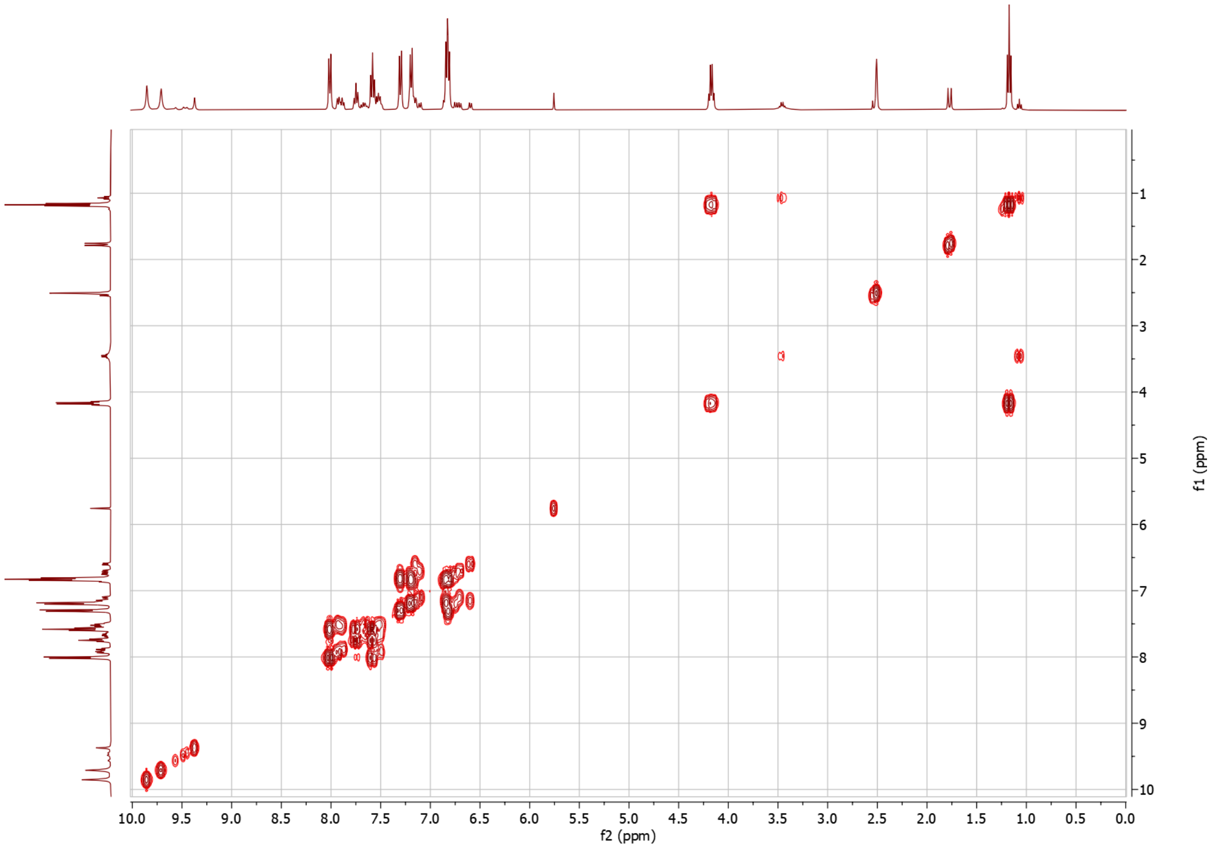

Figure S26. NOESY spectrum of compound **5** in DMSO-d_6.

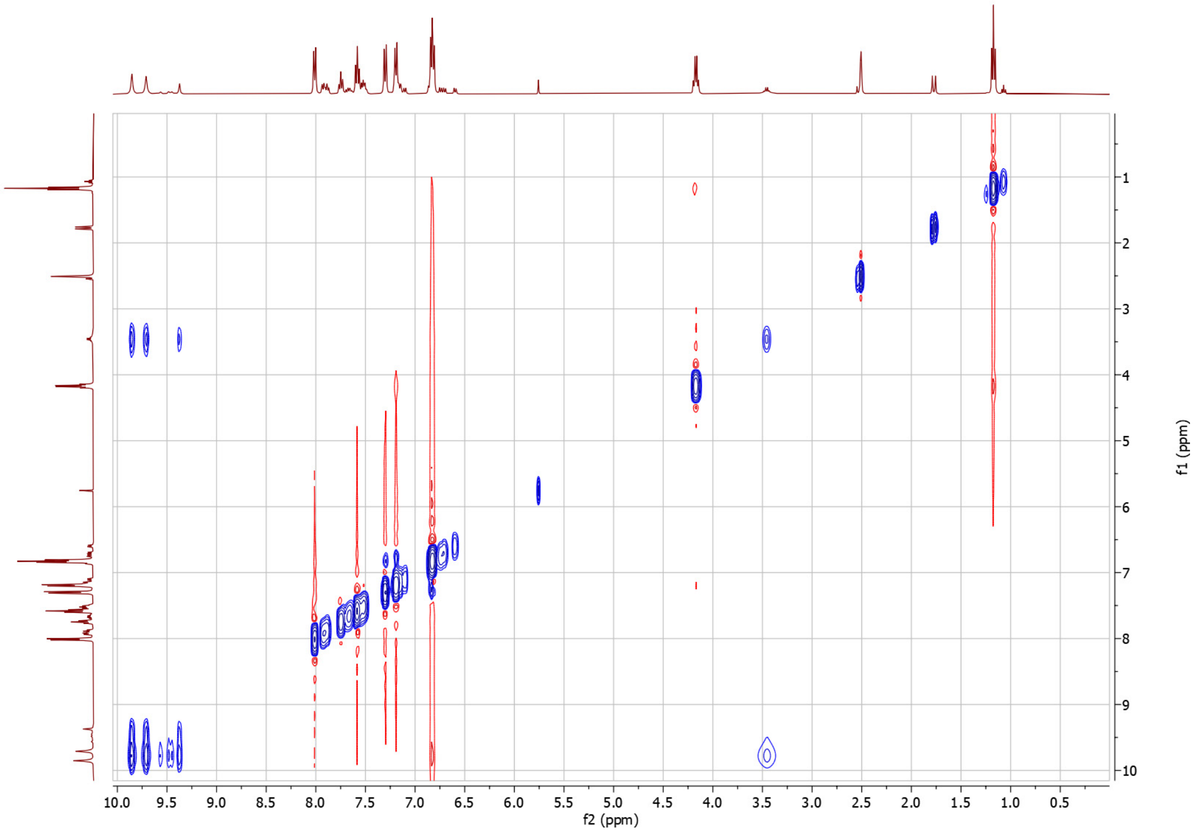

Figure S27. C^{13} NMR spectrum of compound **5** in DMSO-d_6.

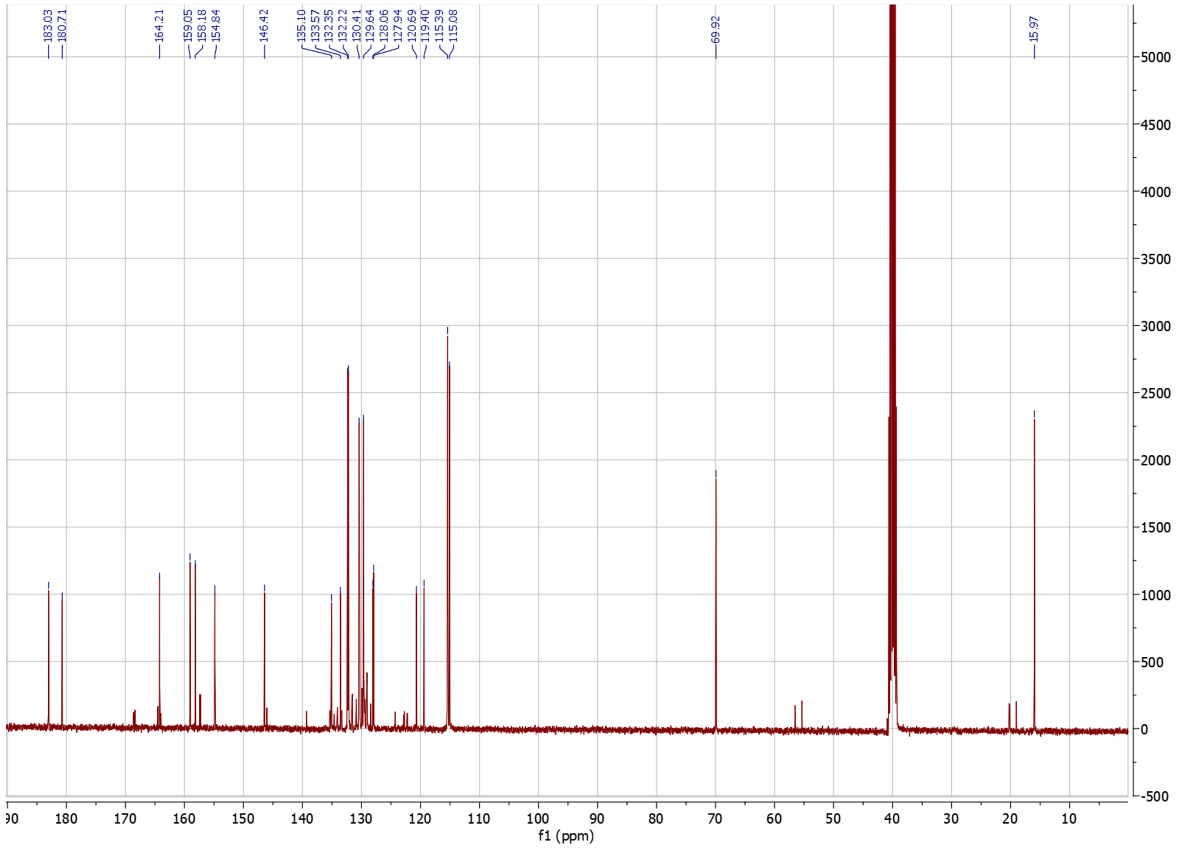

Figure S28. HSQC spectrum of compound **5** in DMSO-d_6.

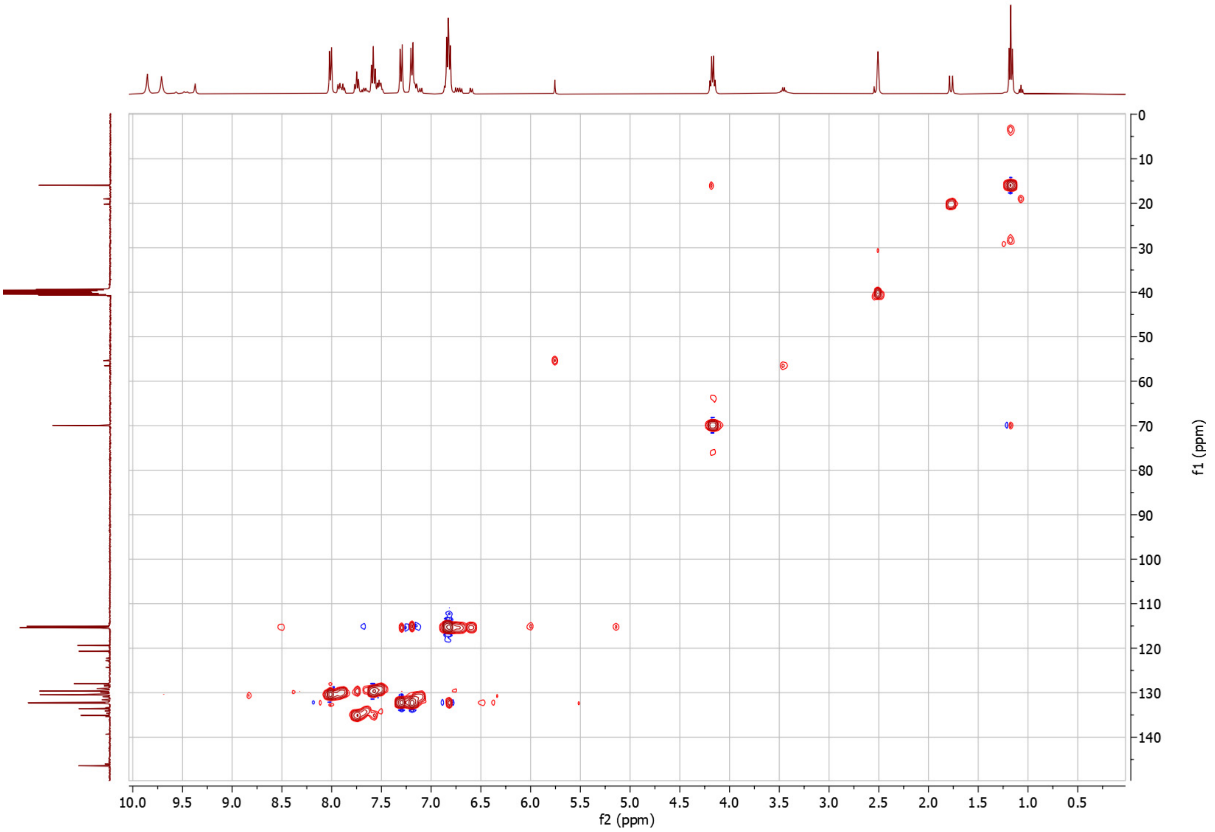

Figure S29. HMBC spectrum of compound **5** in DMSO-d_6.

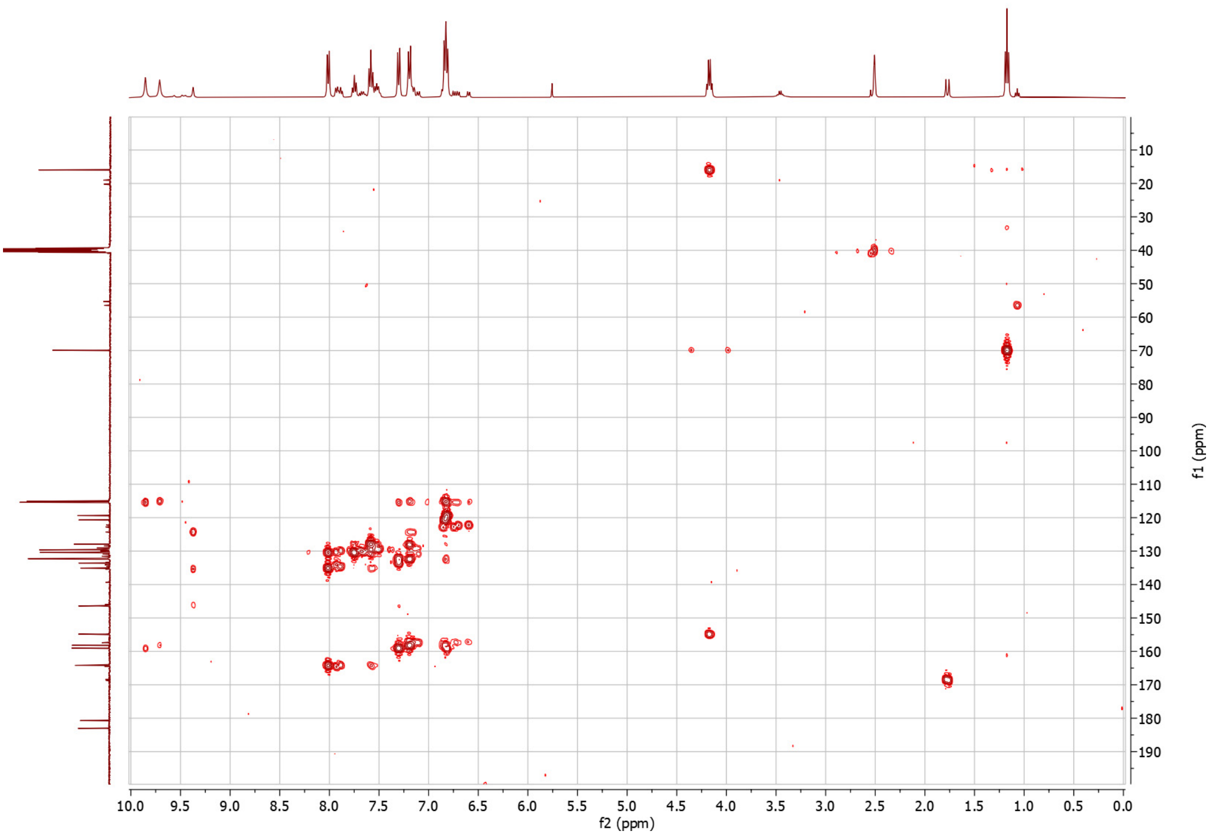

Figure S30. ^{1}H NMR spectrum of compound **6** in DMSO-d_6.

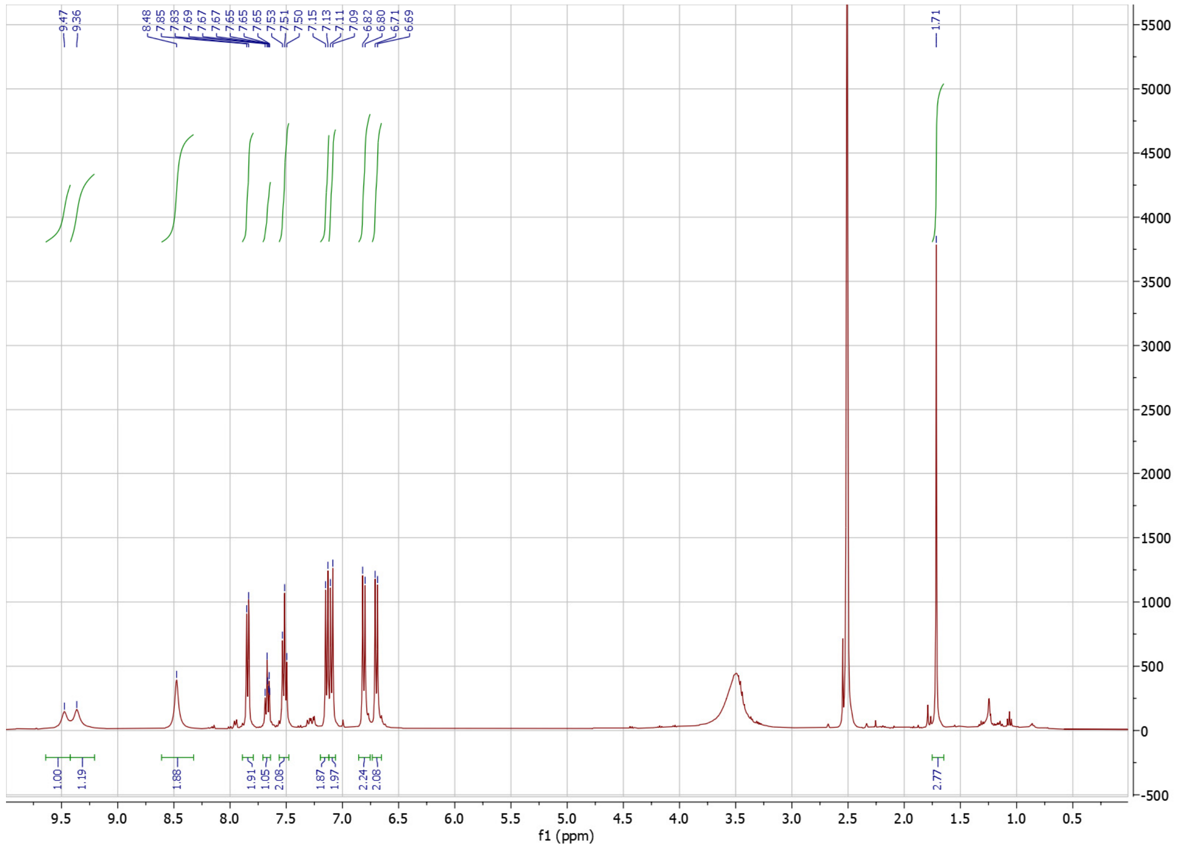

Figure S31. COSY spectrum of compound **6** in DMSO-d_6.

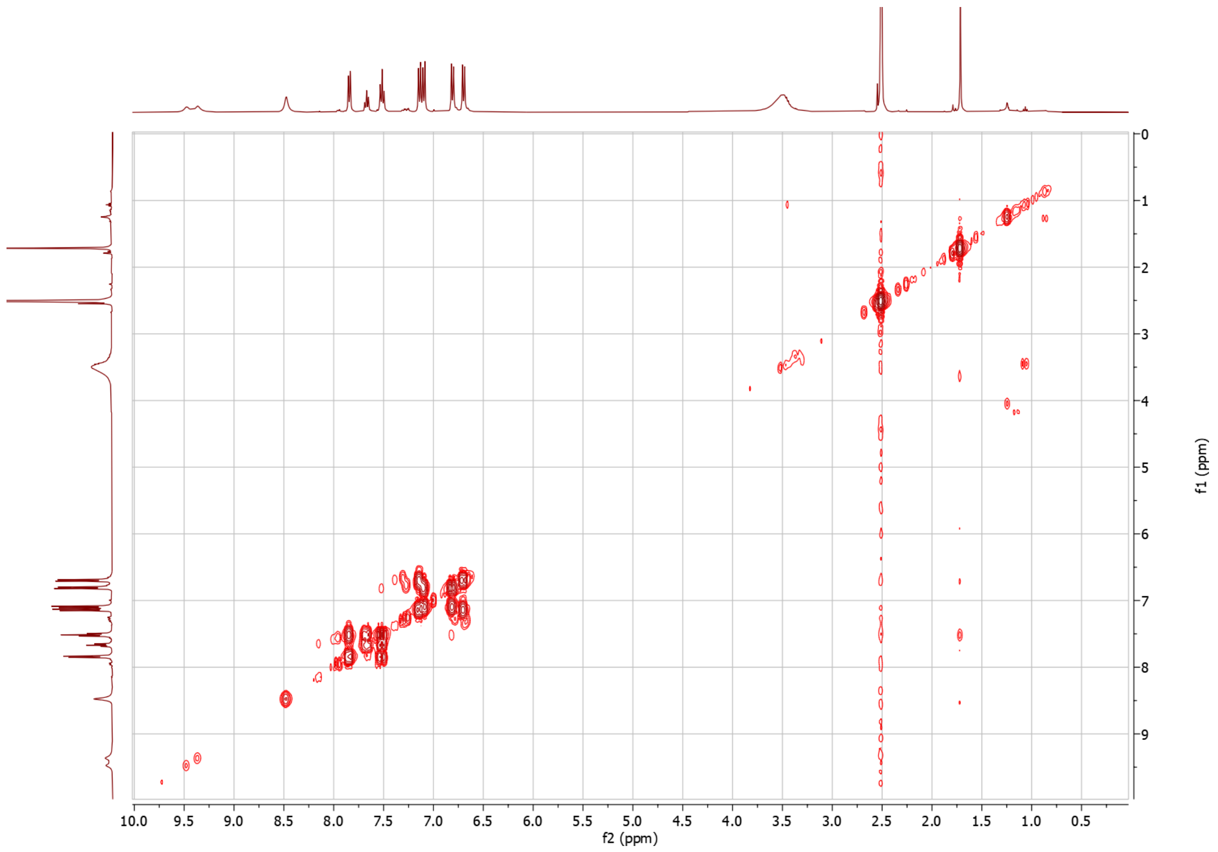

Figure S32. NOESY spectrum of compound **6** in DMSO-d_6.

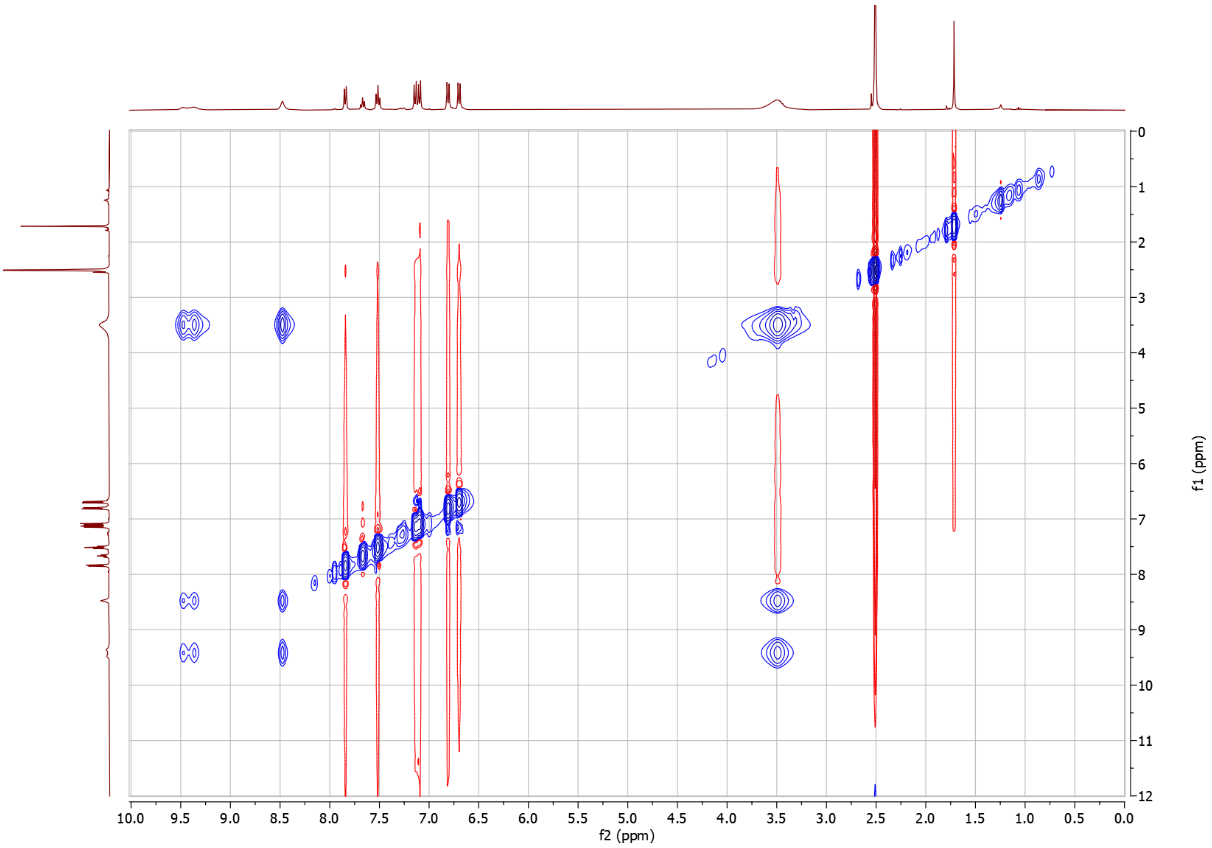

Figure S33. C[13] NMR spectrum of compound **6** in DMSO-d_6.

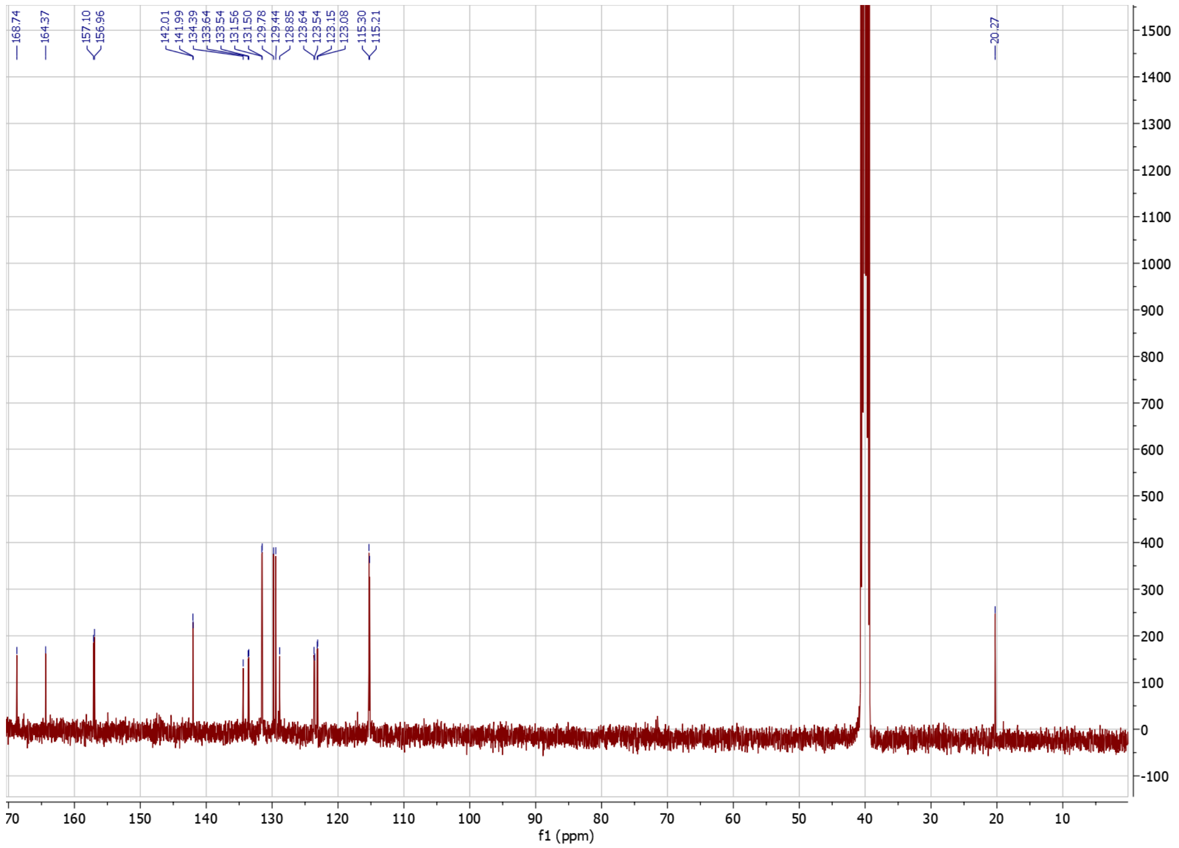

Figure S34. HSQC spectrum of compound **6** in DMSO-d_6.

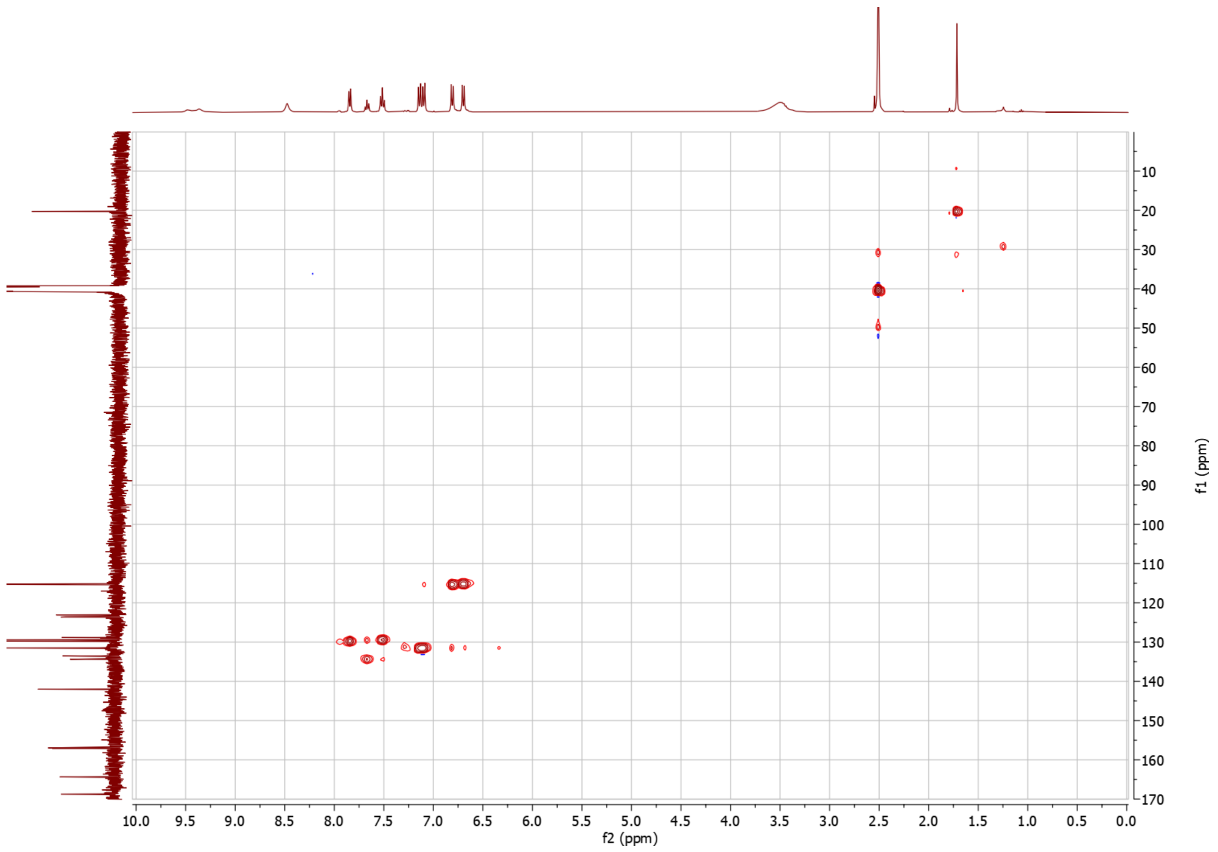

Figure S35. HMBC spectrum of compound **6** in DMSO-d_6.

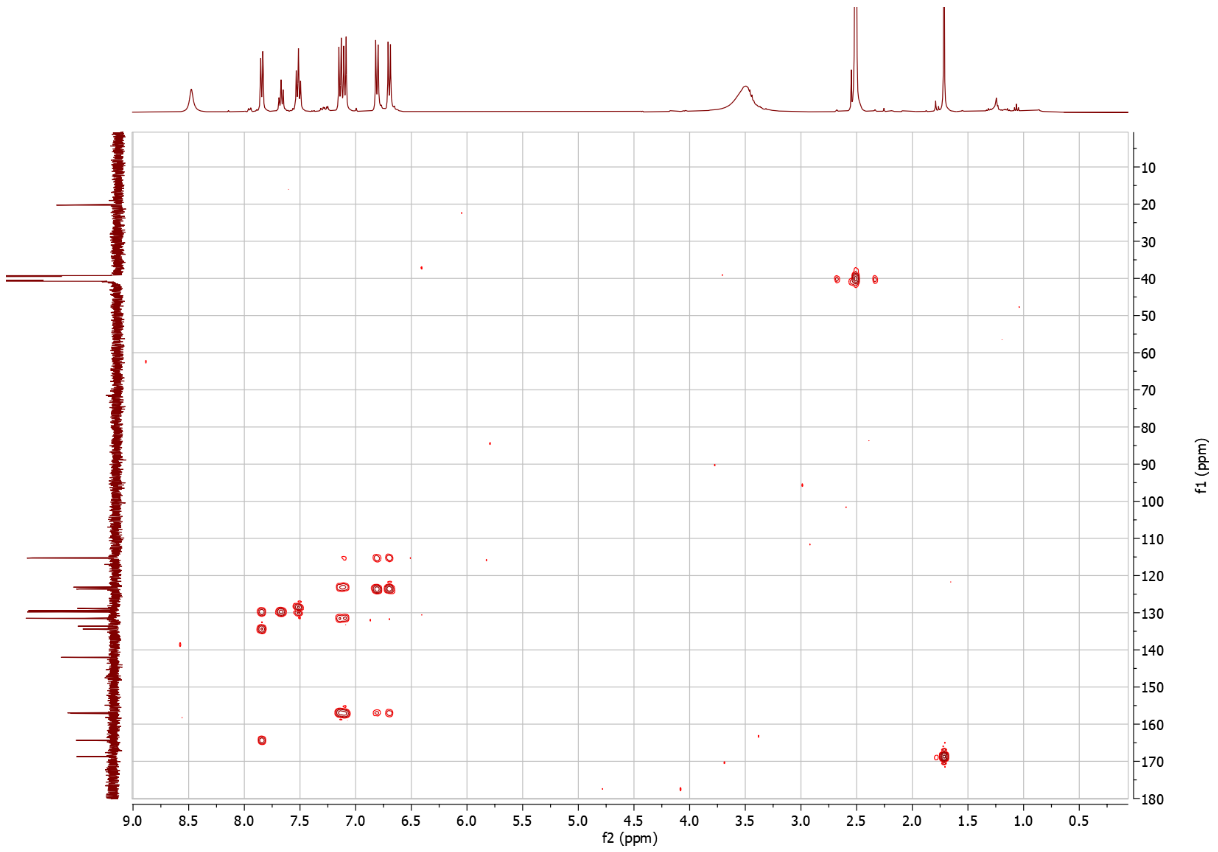

Figure S36. ^{1}H NMR spectrum of compound **7** in DMSO-d_6.

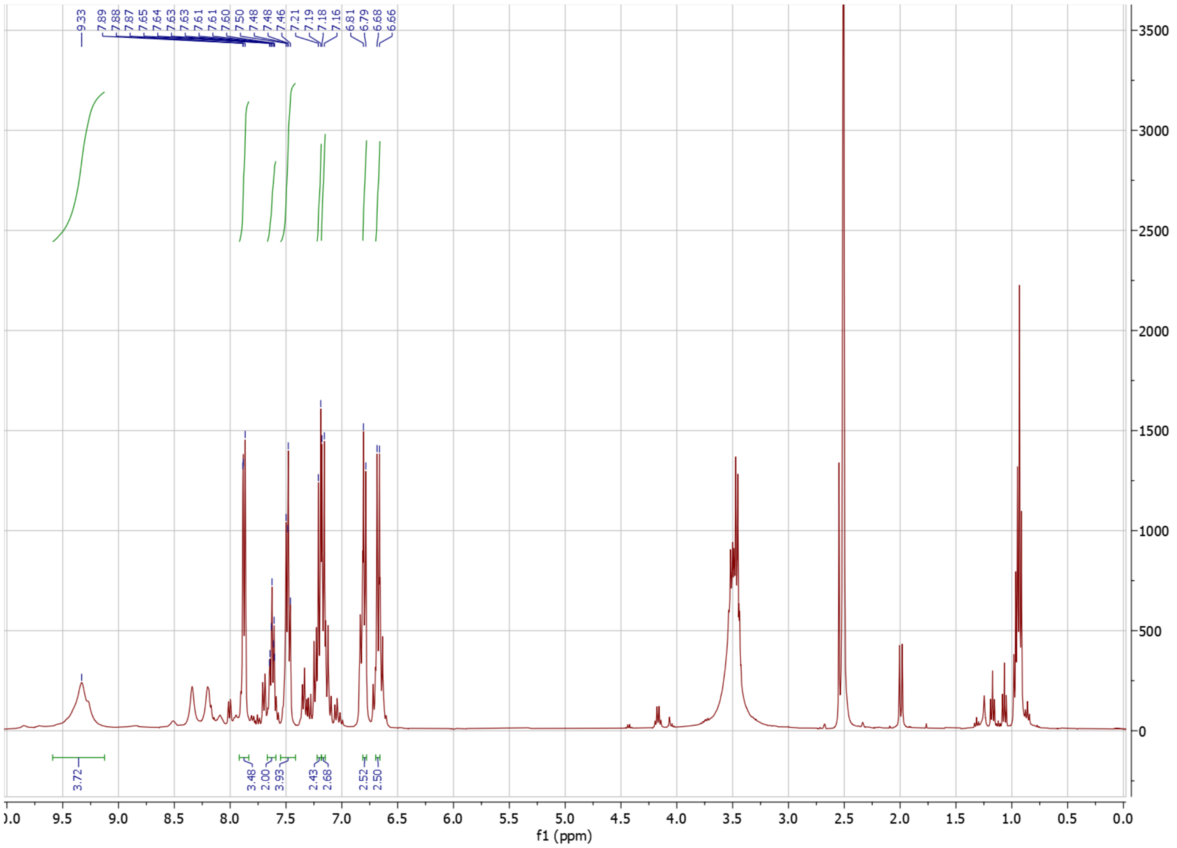

Figure S37. COSY spectrum of compound **7** in DMSO-*d*6.

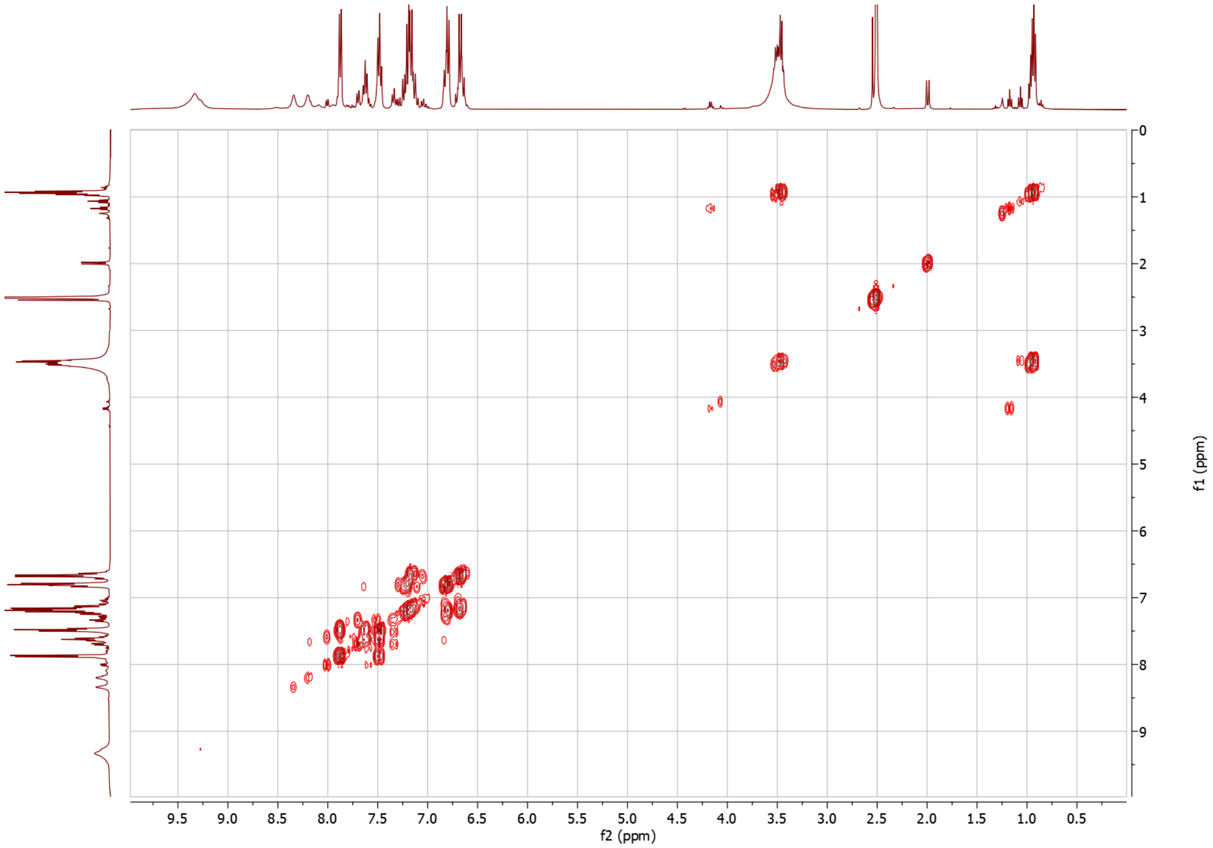

Figure S38. NOESY spectrum of compound **7** in DMSO-d_6.

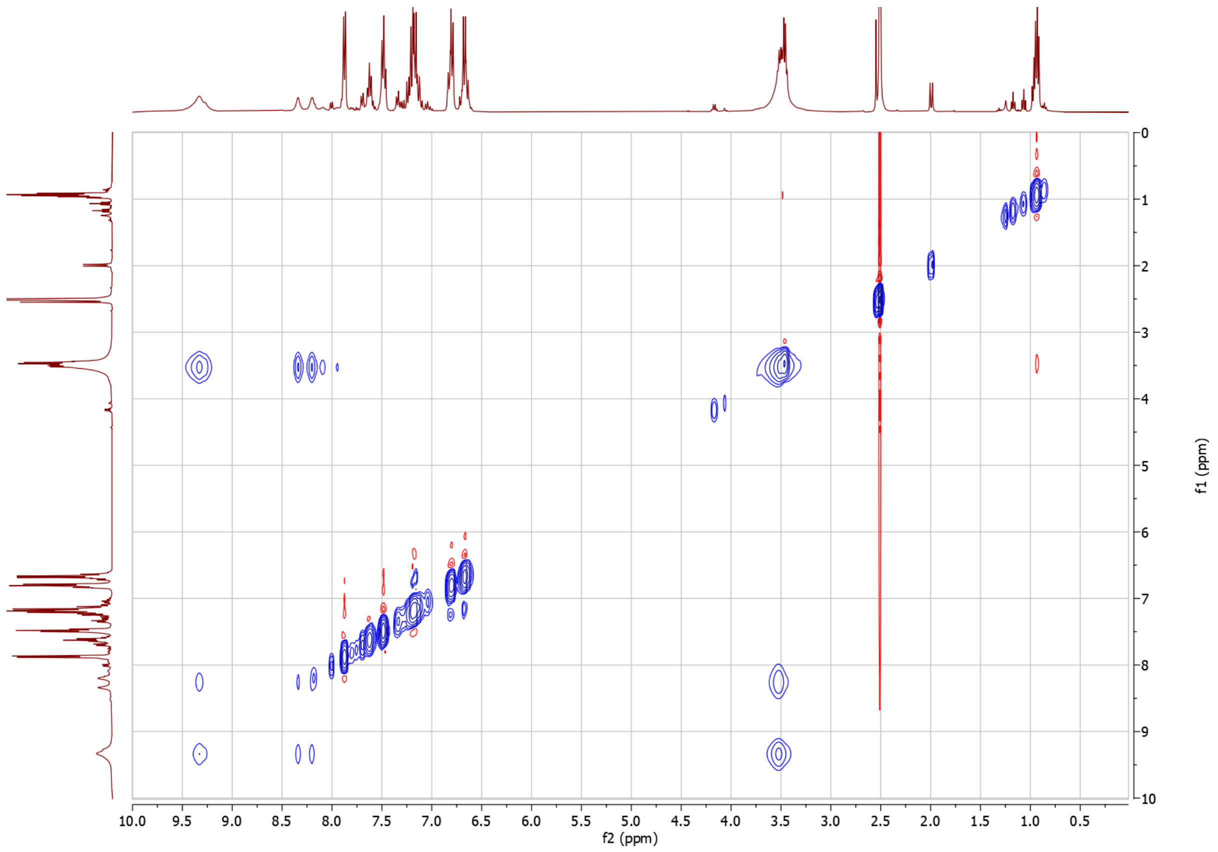

Figure S39. C[13] NMR spectrum of compound **7** in DMSO-d_6.

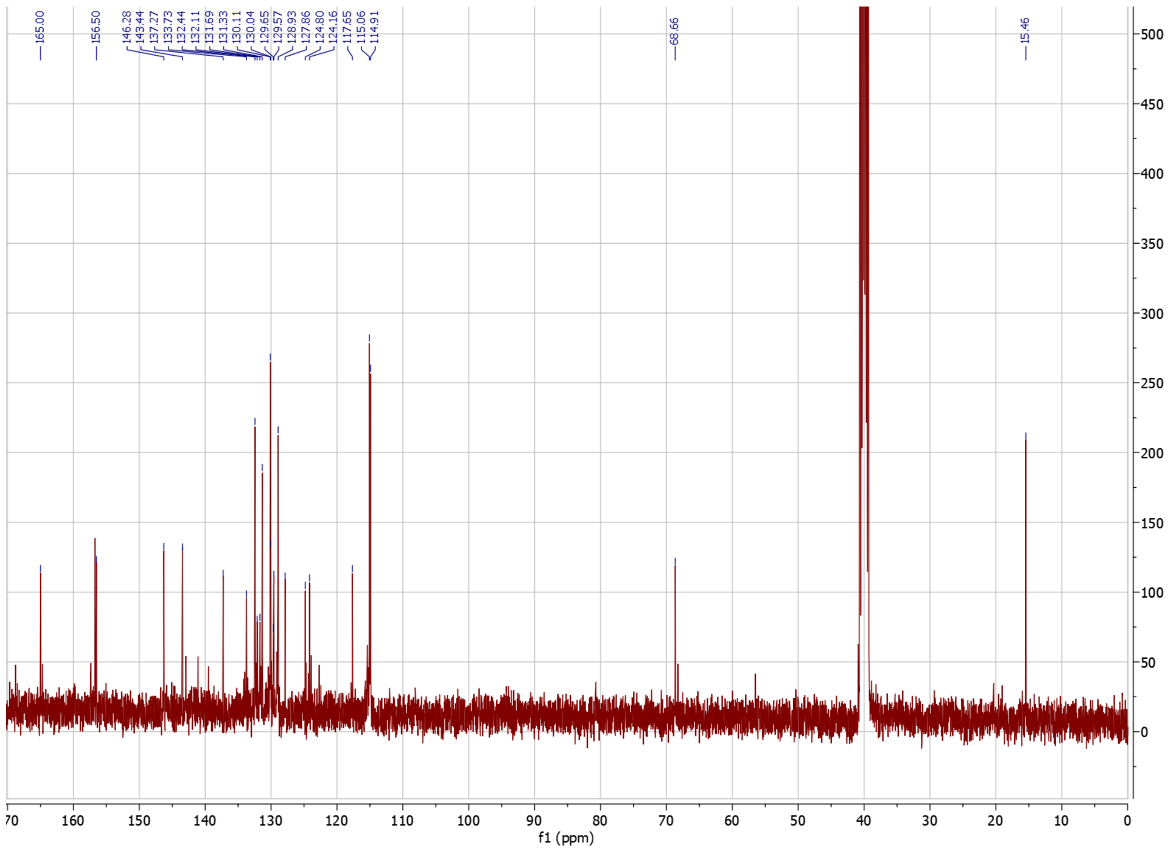

Figure S40. HSQC spectrum of compound **7** in DMSO-d_6.

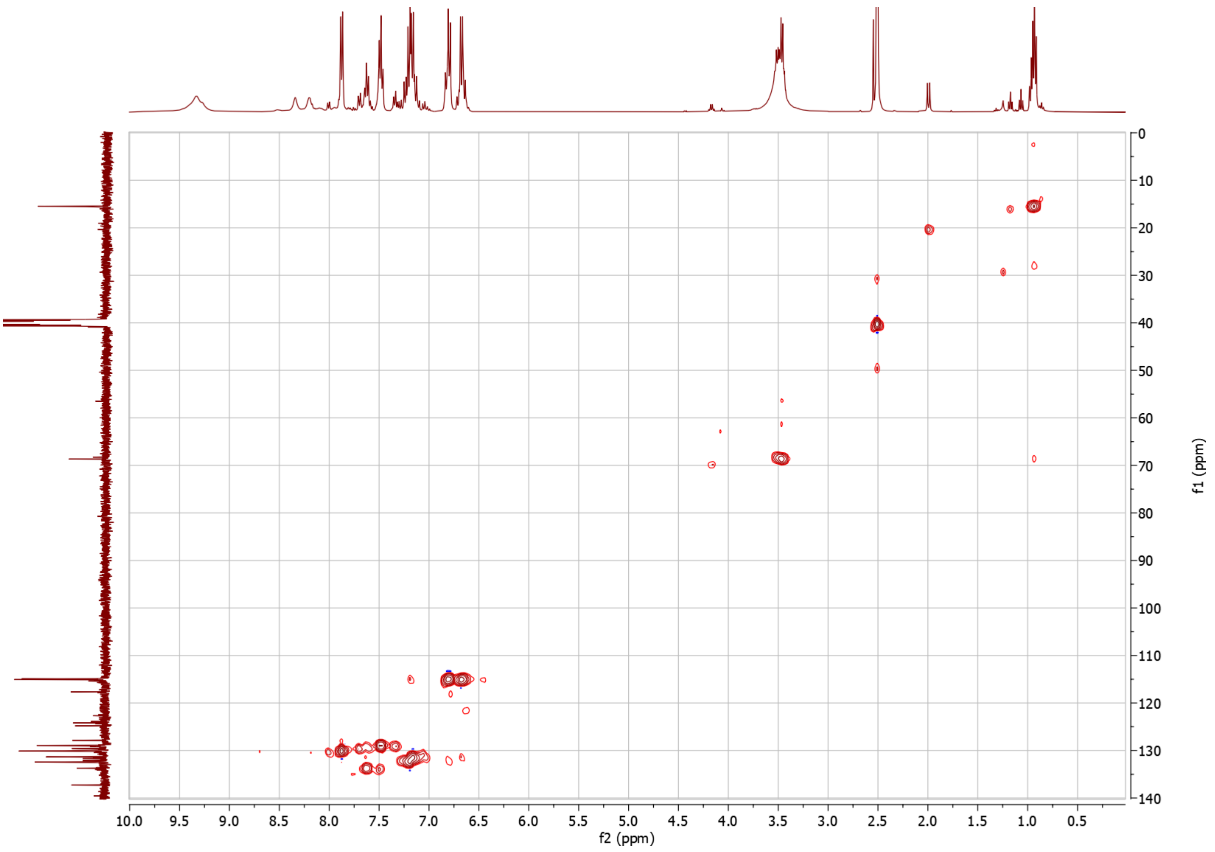

Figure S41. HMBC spectrum of compound **7** in DMSO-d_6.

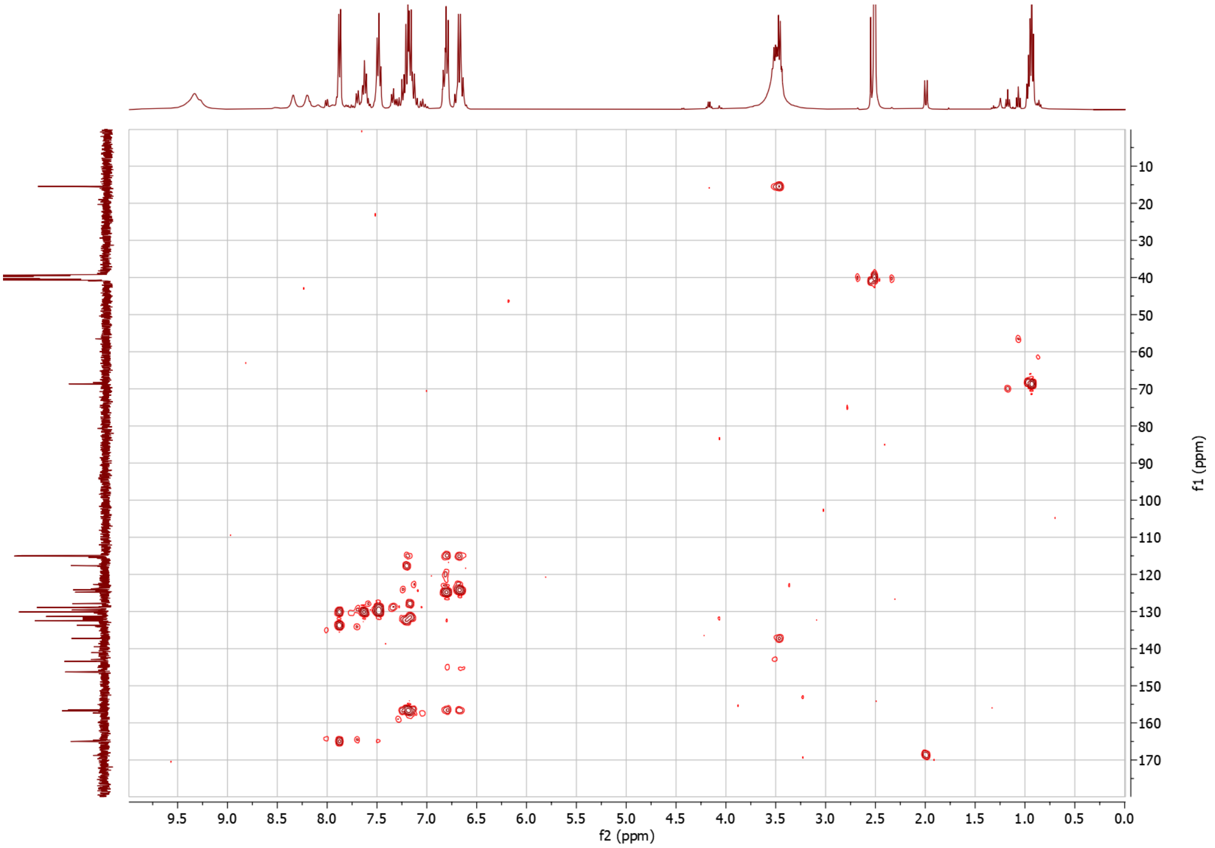

Figure S42. ^1H NMR spectrum of compound **8** in DMSO-d_6.

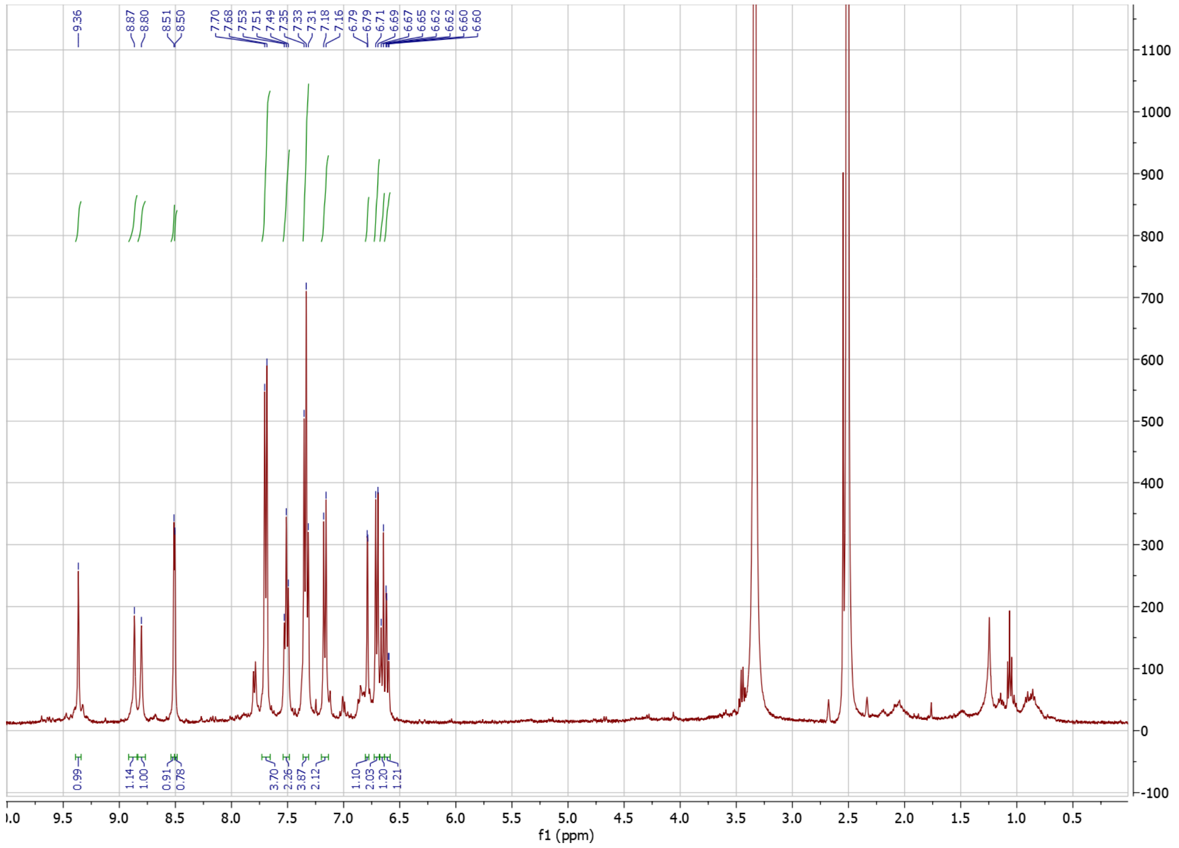

Figure S43. COSY spectrum of compound **8** in DMSO-d_6.

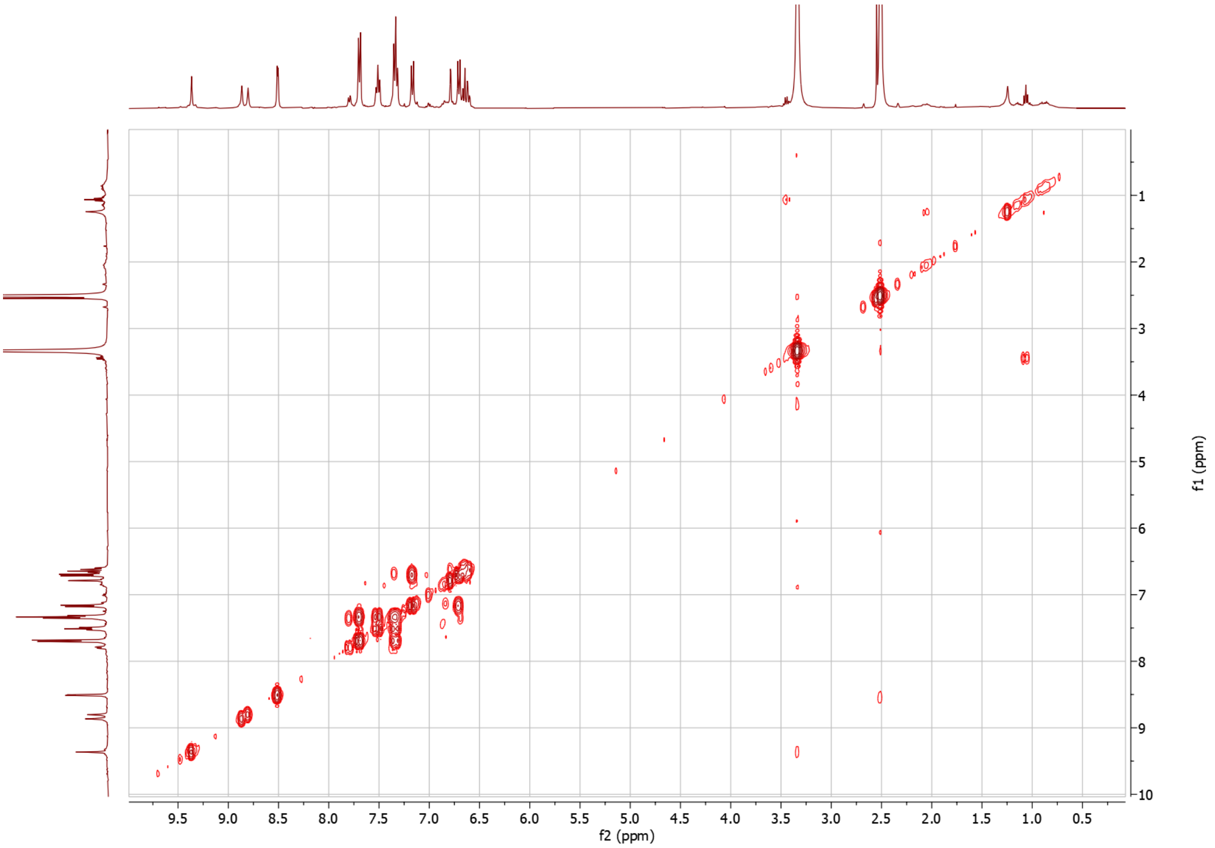

Figure S44. NOESY spectrum of compound **8** in DMSO-d_6.

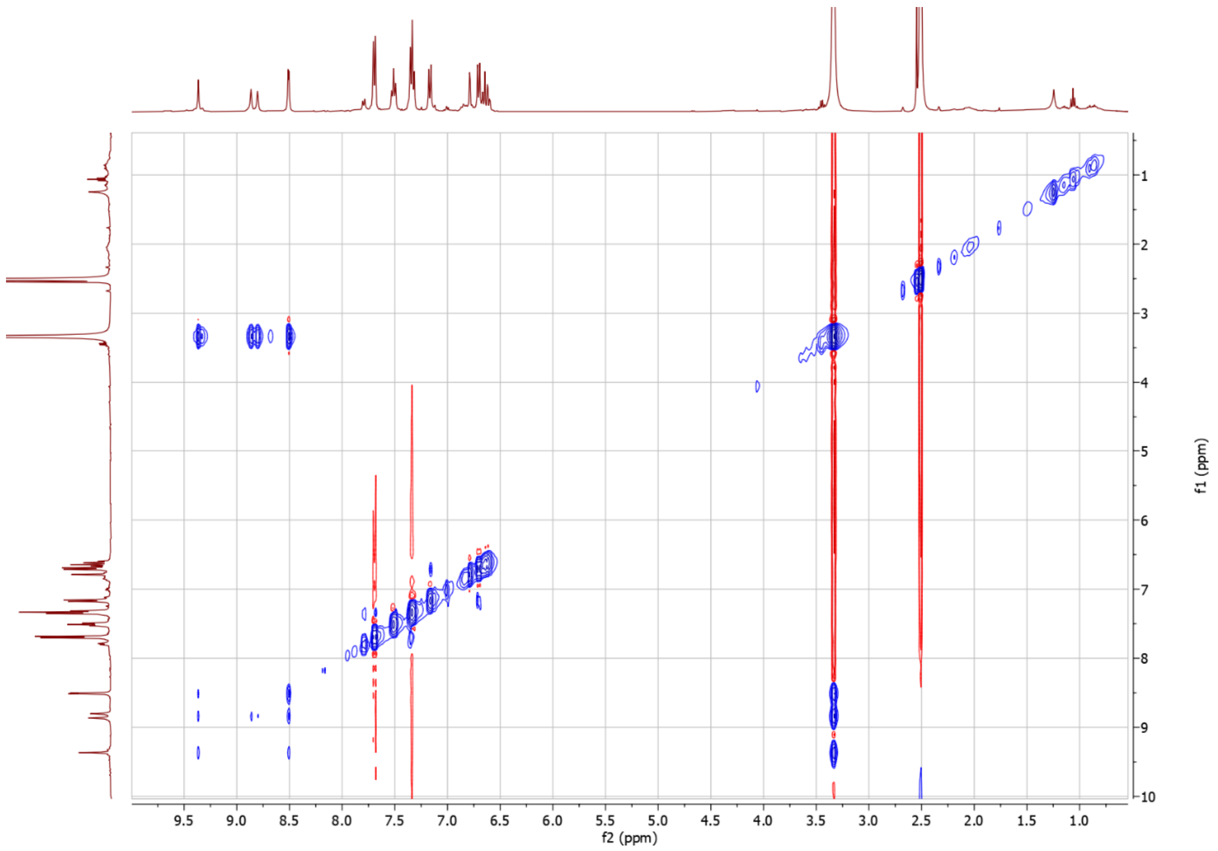

Figure S45. C^{13} NMR spectrum of compound **8** in DMSO-d_6.

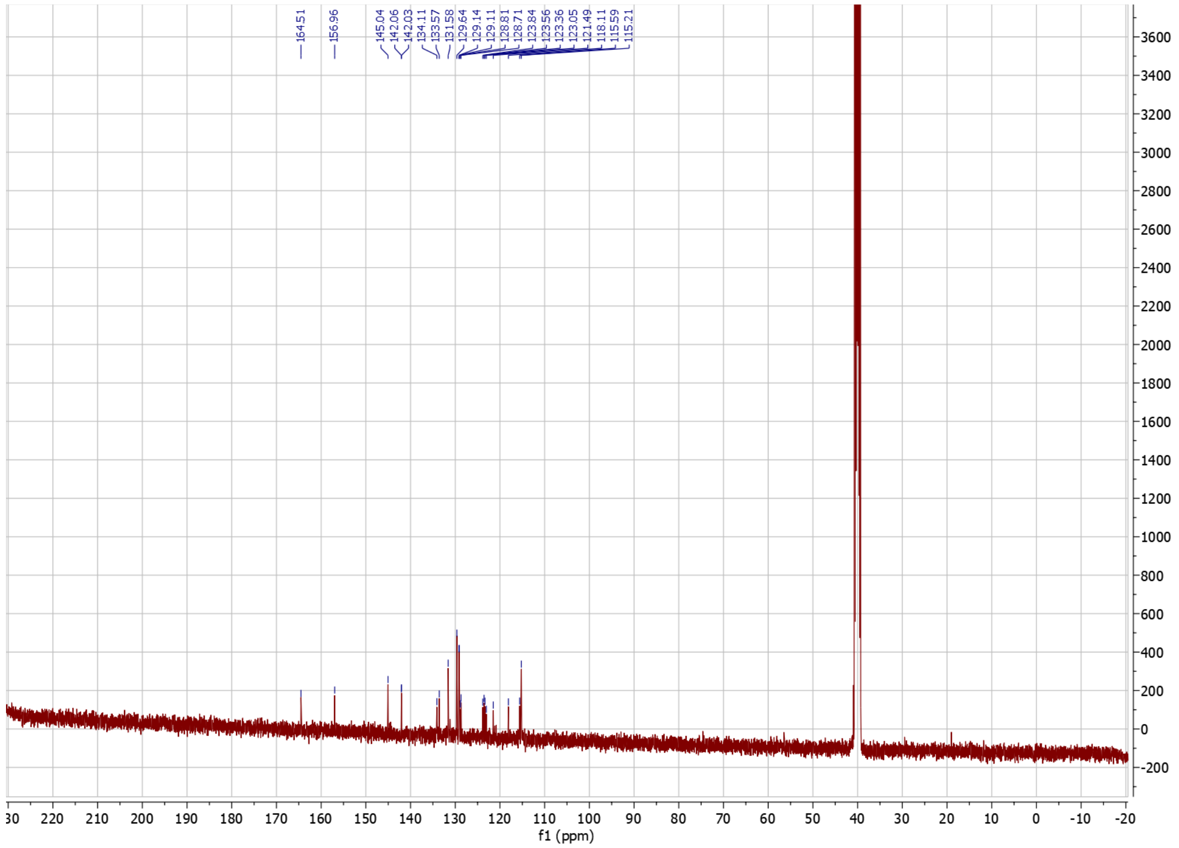

Figure S46. HSQC spectrum of compound **8** in DMSO-*d*$_6$.

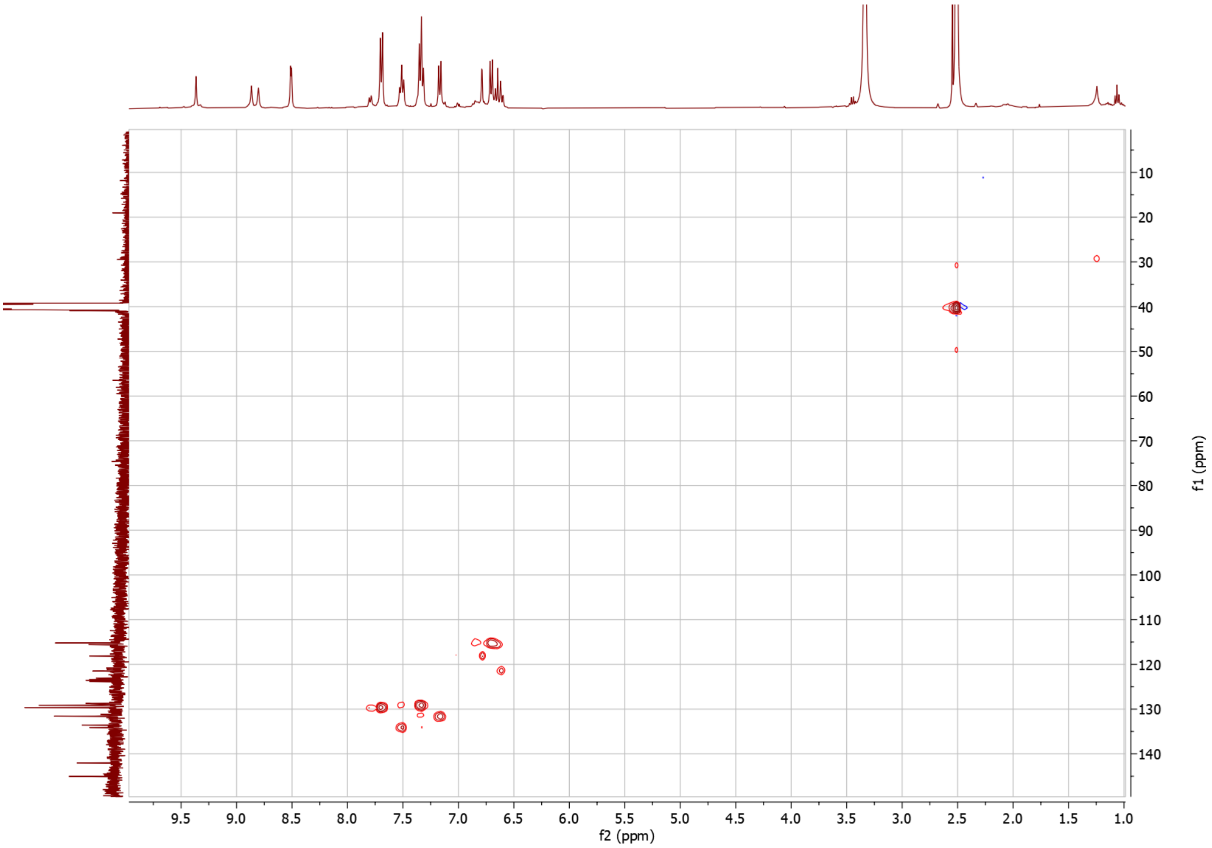

Figure S47. HMBC spectrum of compound **8** in DMSO-d_6.

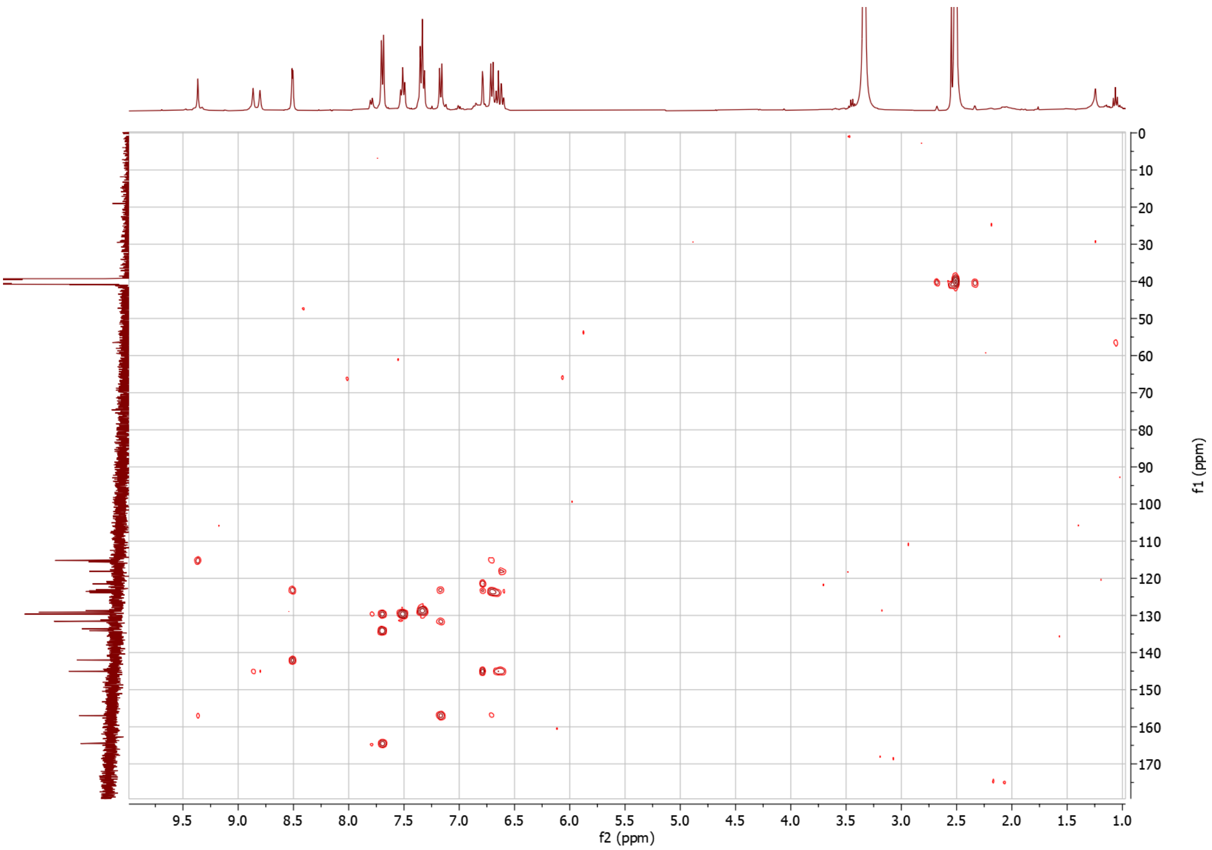

Figure S48. ^{1}H NMR spectrum of compound **9** in DMSO-d_6.

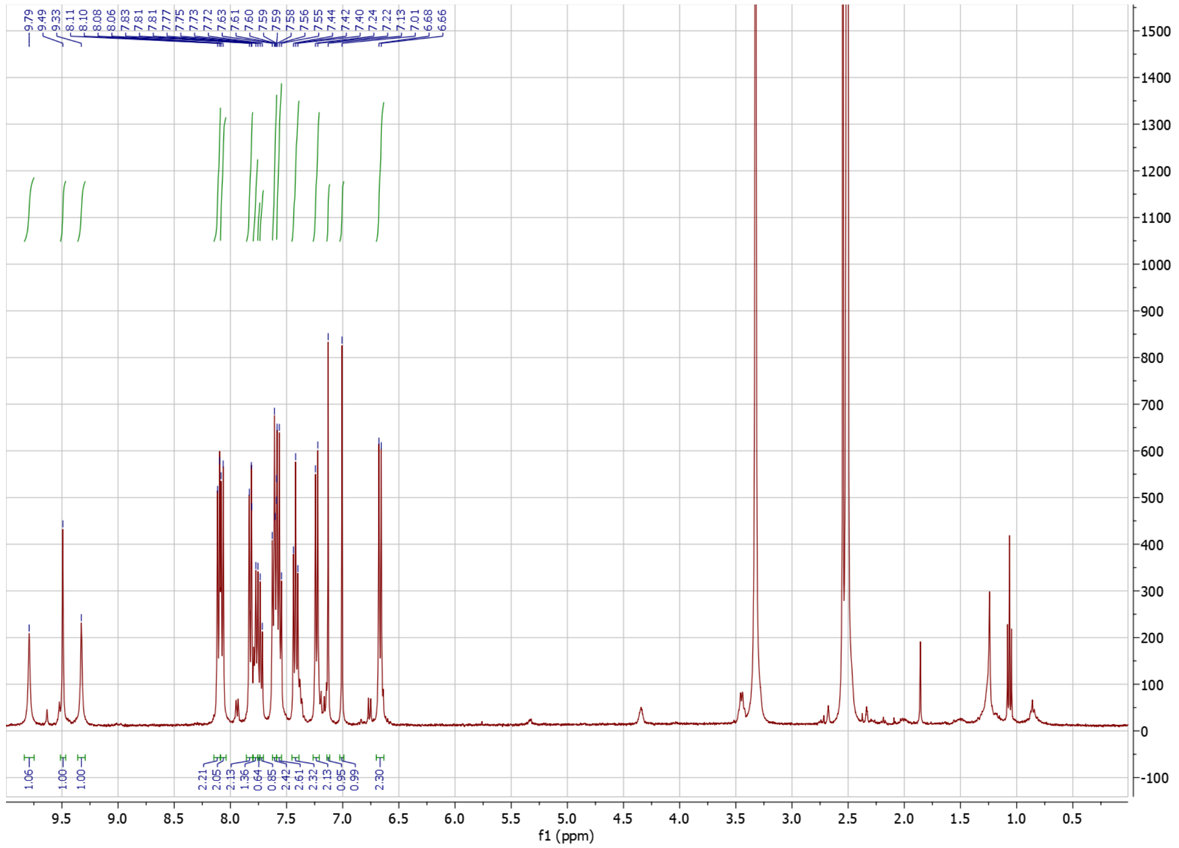

Figure S49. COSY spectrum of compound **9** in DMSO-*d*₆.

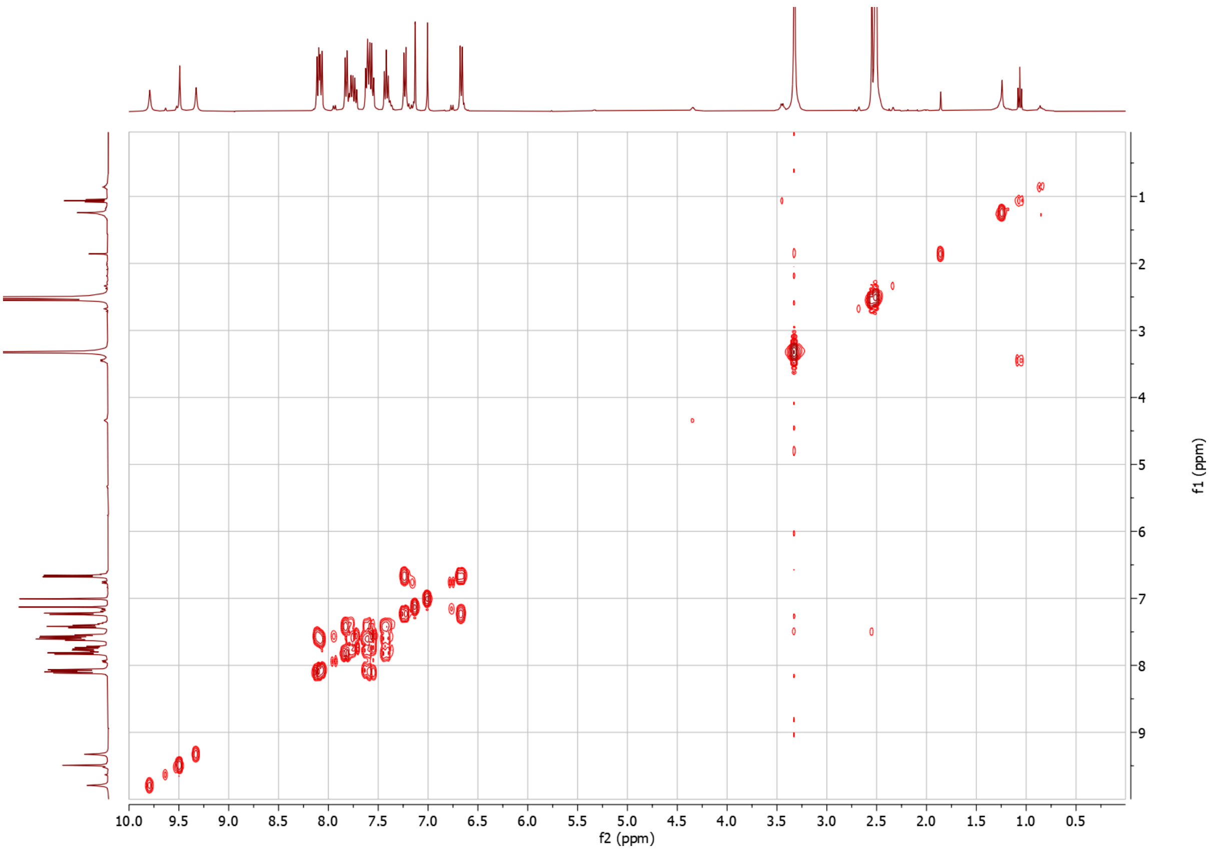

Figure S50. NOESY spectrum of compound **9** in DMSO-d_6.

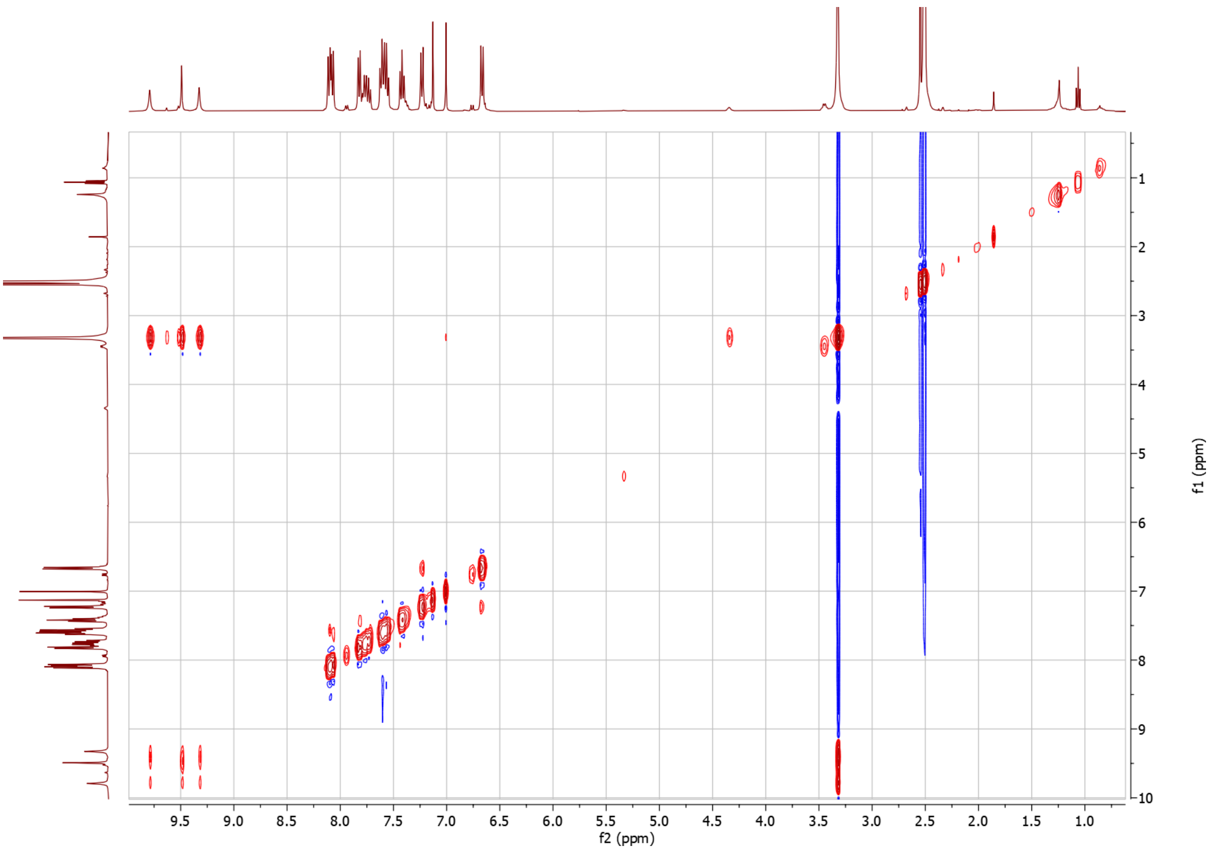

Figure S51. C^{13} NMR spectrum of compound **9** in DMSO-d_6.

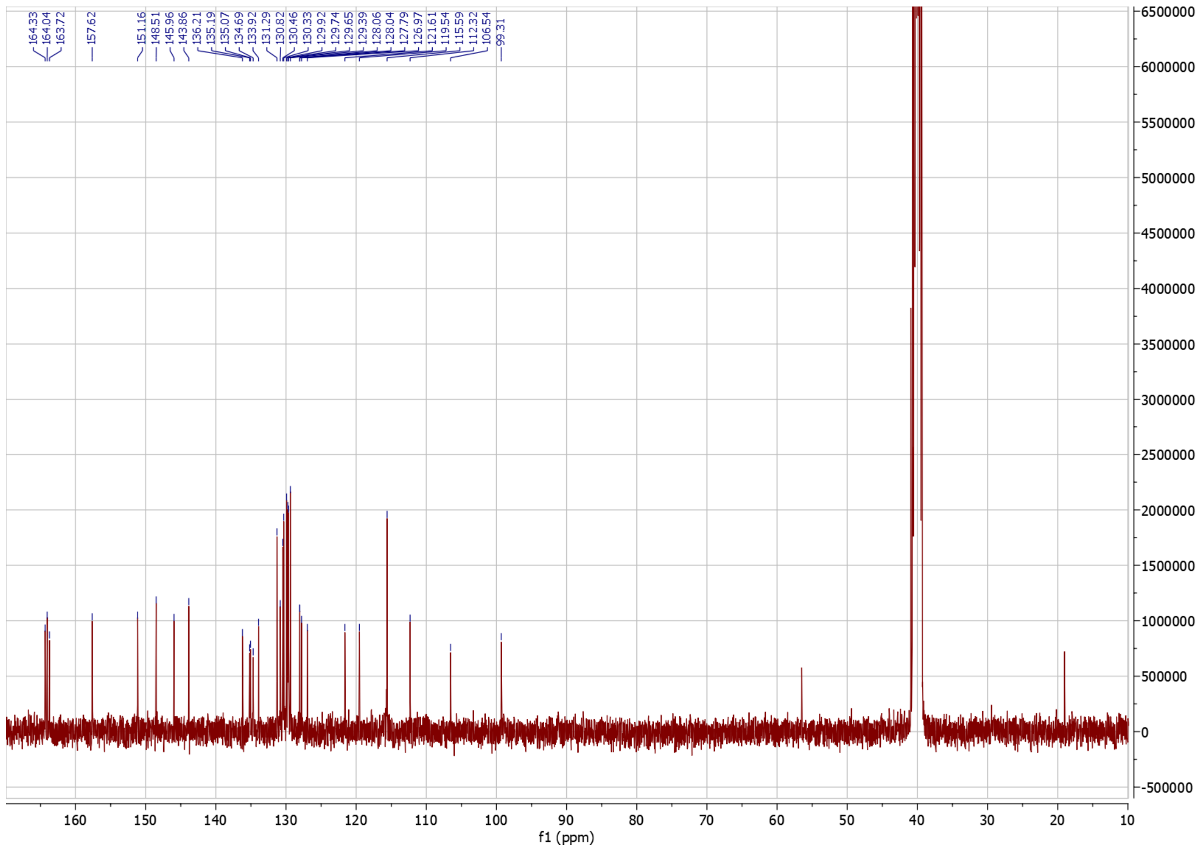

Figure S52. HSQC spectrum of compound **9** in DMSO-d_6.

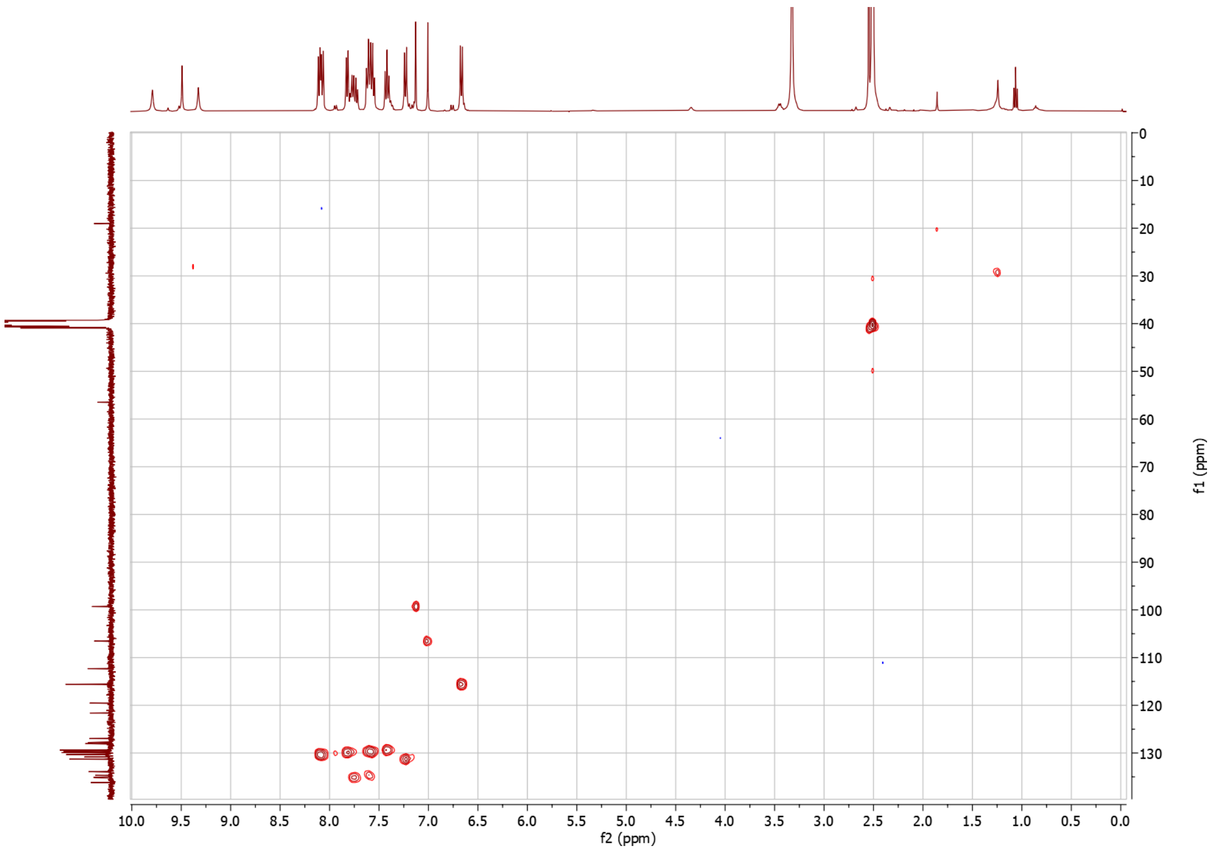

Figure S53. HMBC spectrum of compound **9** in DMSO-d_6.

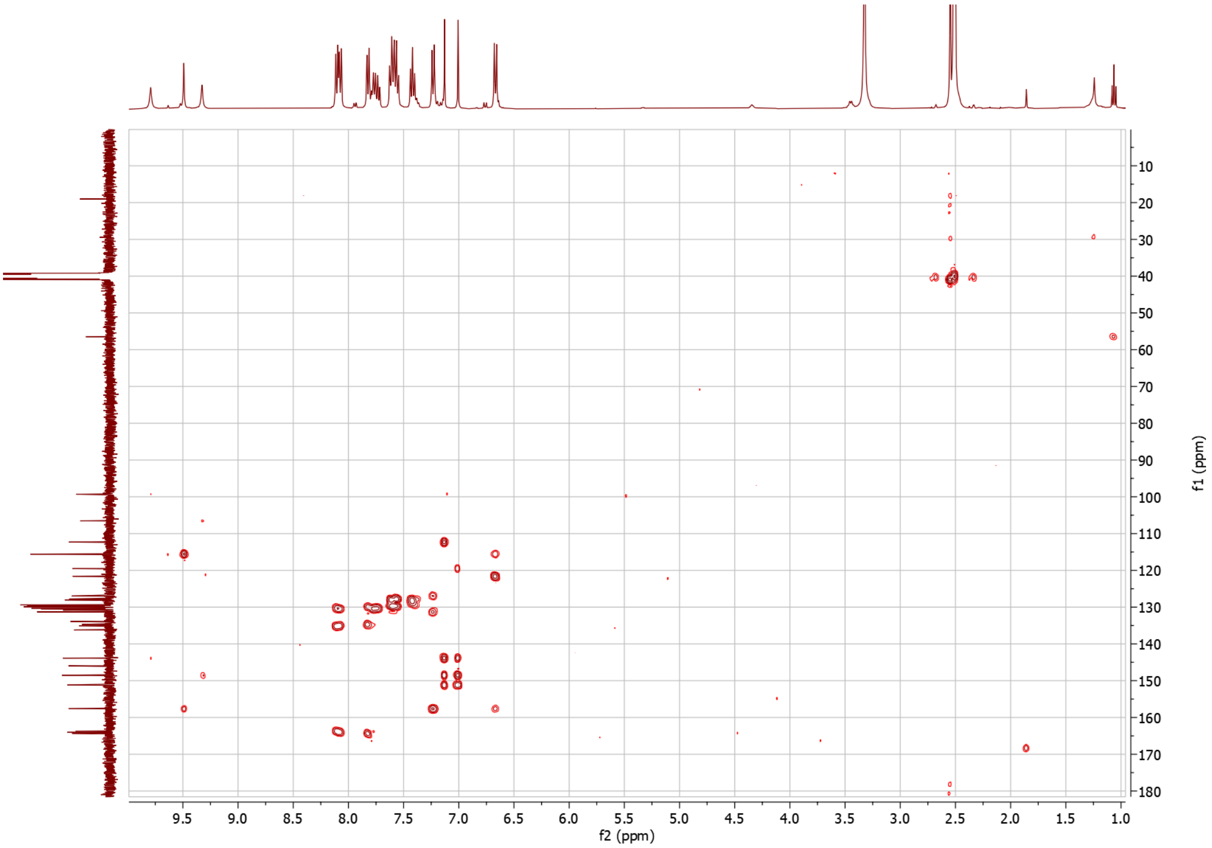

Figure S54. HR-ESI-MS spectrum of compound **1**.

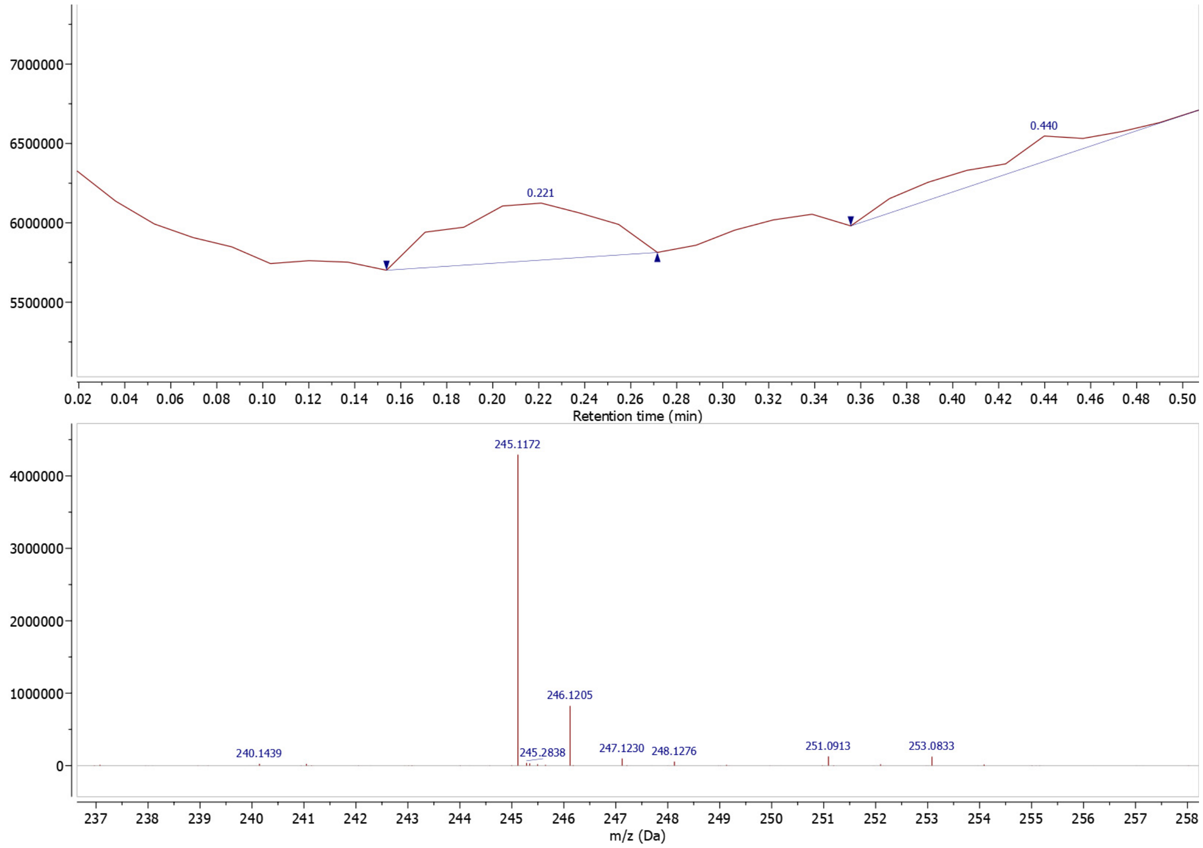

Figure S55. HR-ESI-MS spectrum of compound **2**.

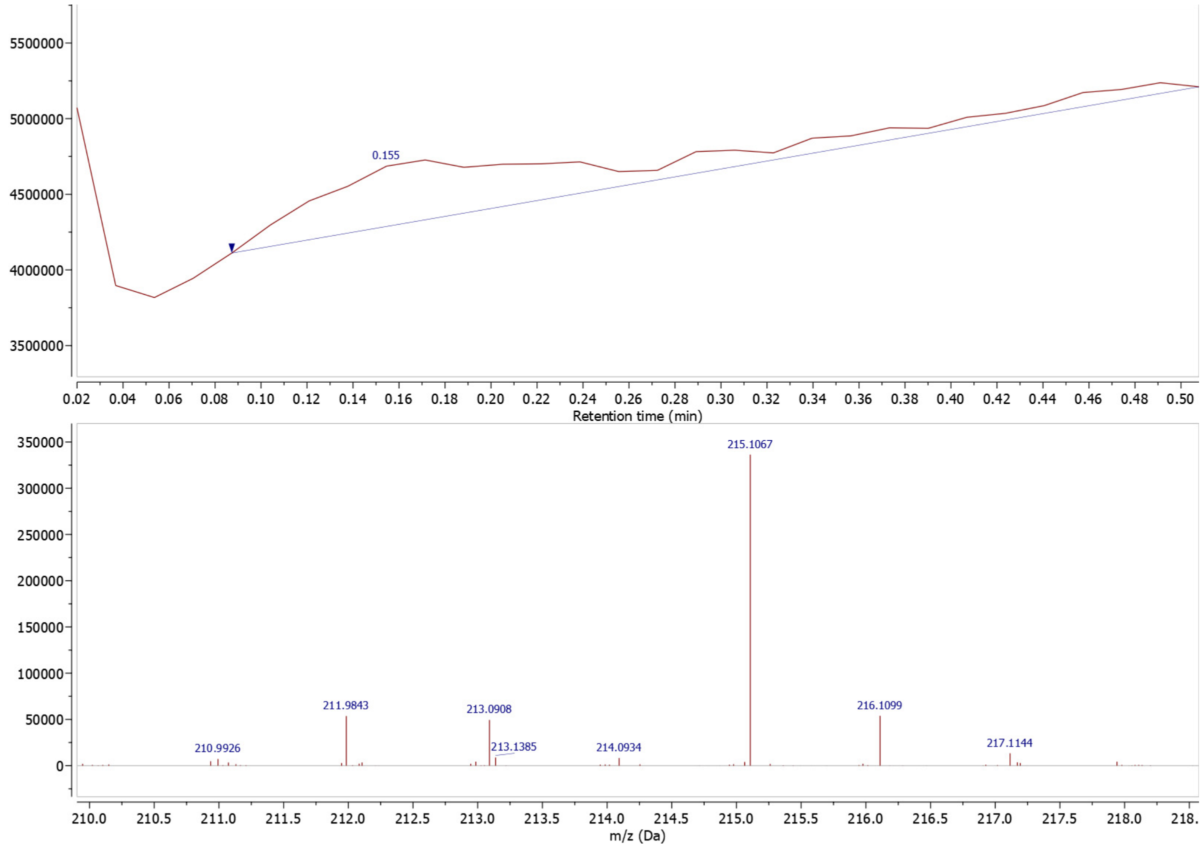

Figure S56. HR-ESI-MS spectrum of compound **3**.

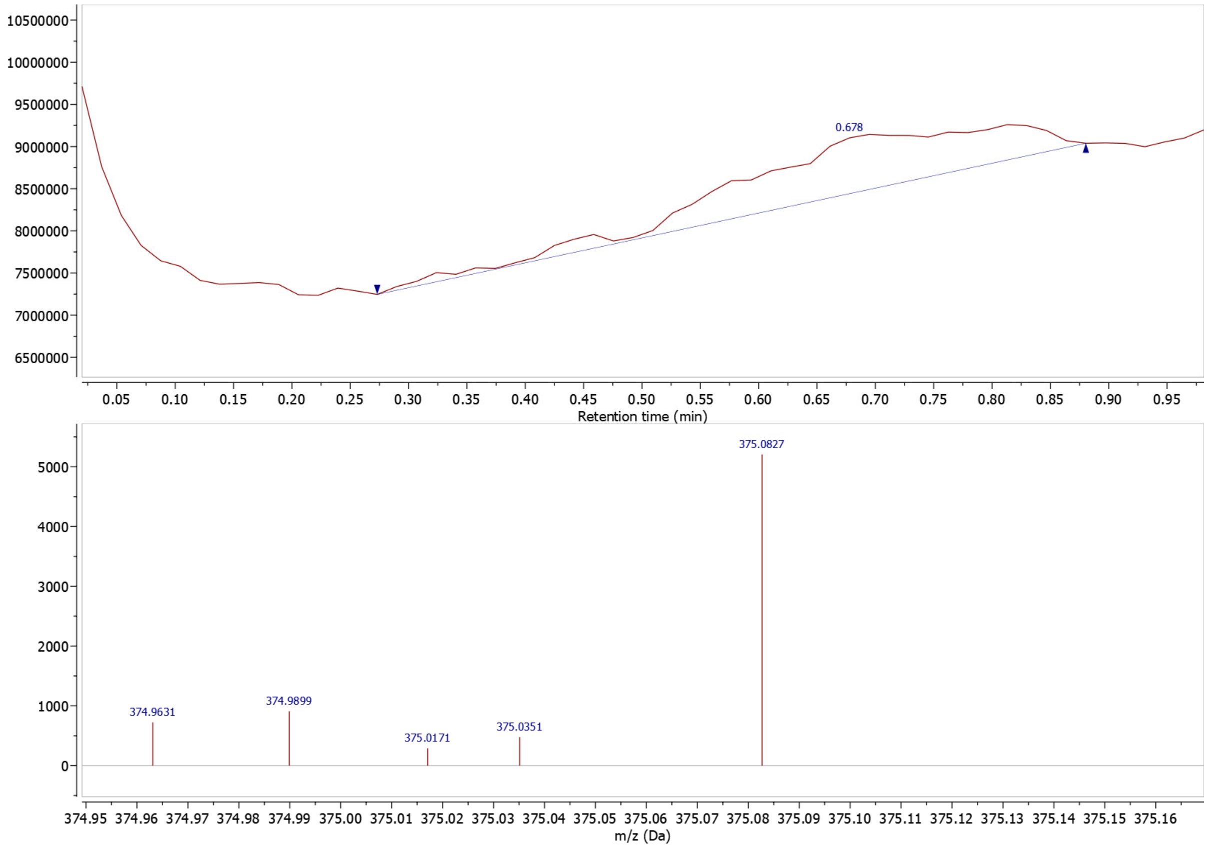

Figure S57. HR-ESI-MS spectrum of compound **5**.

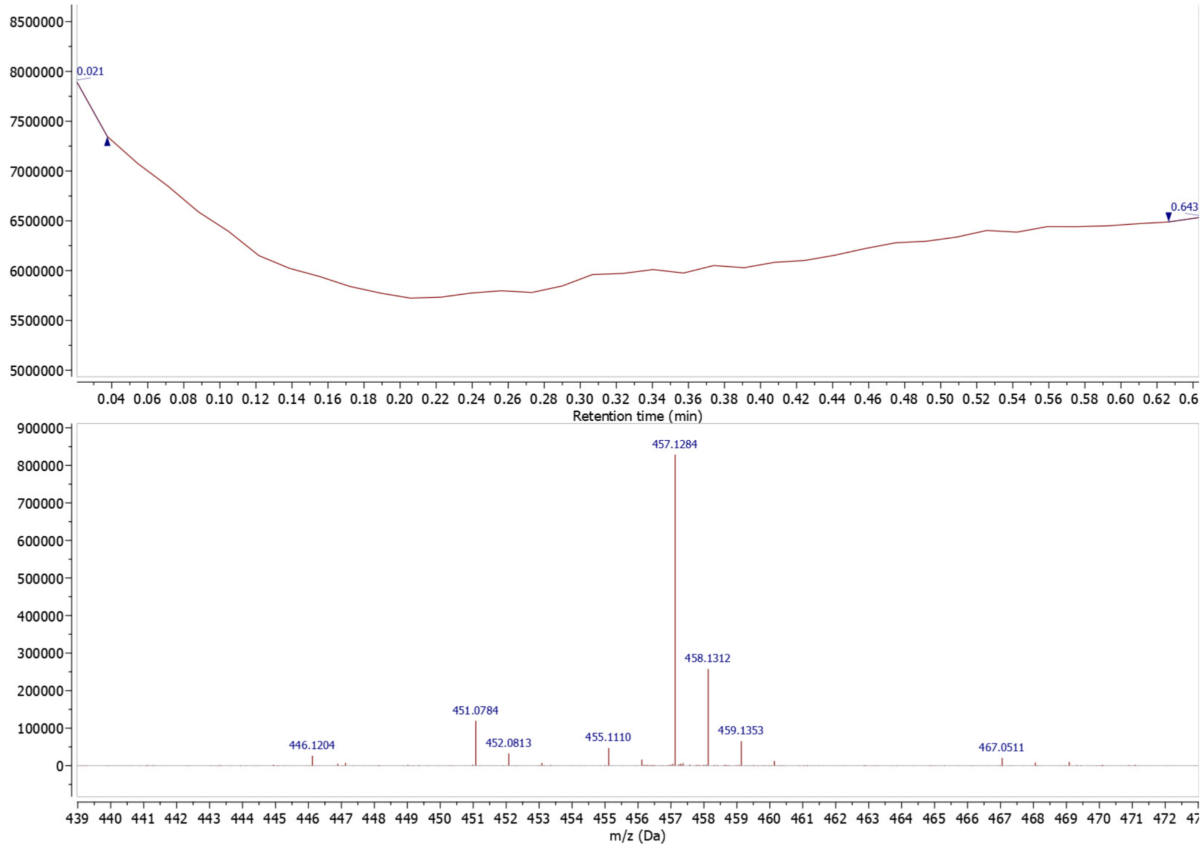

Figure S58. HR-ESI-MS spectrum of compound **6**.

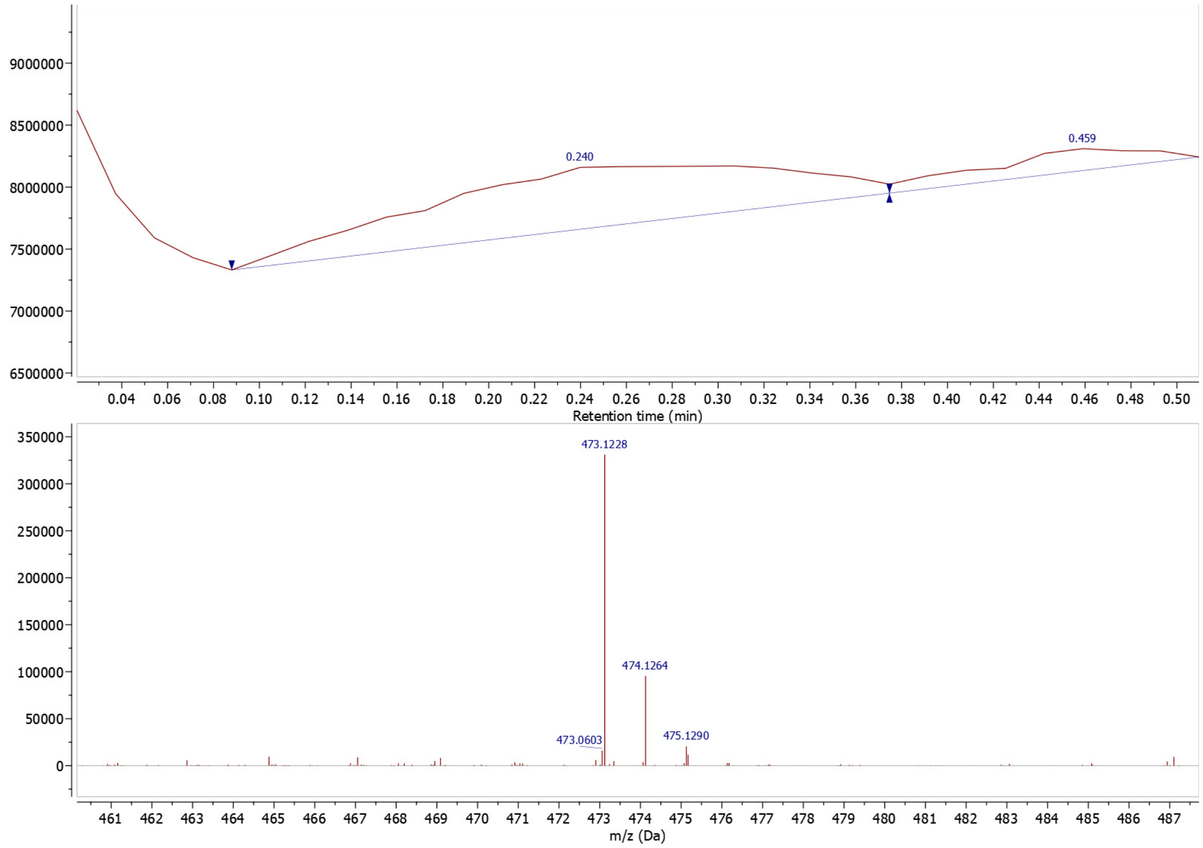

Figure S59. HR-ESI-MS spectrum of compound **7**.

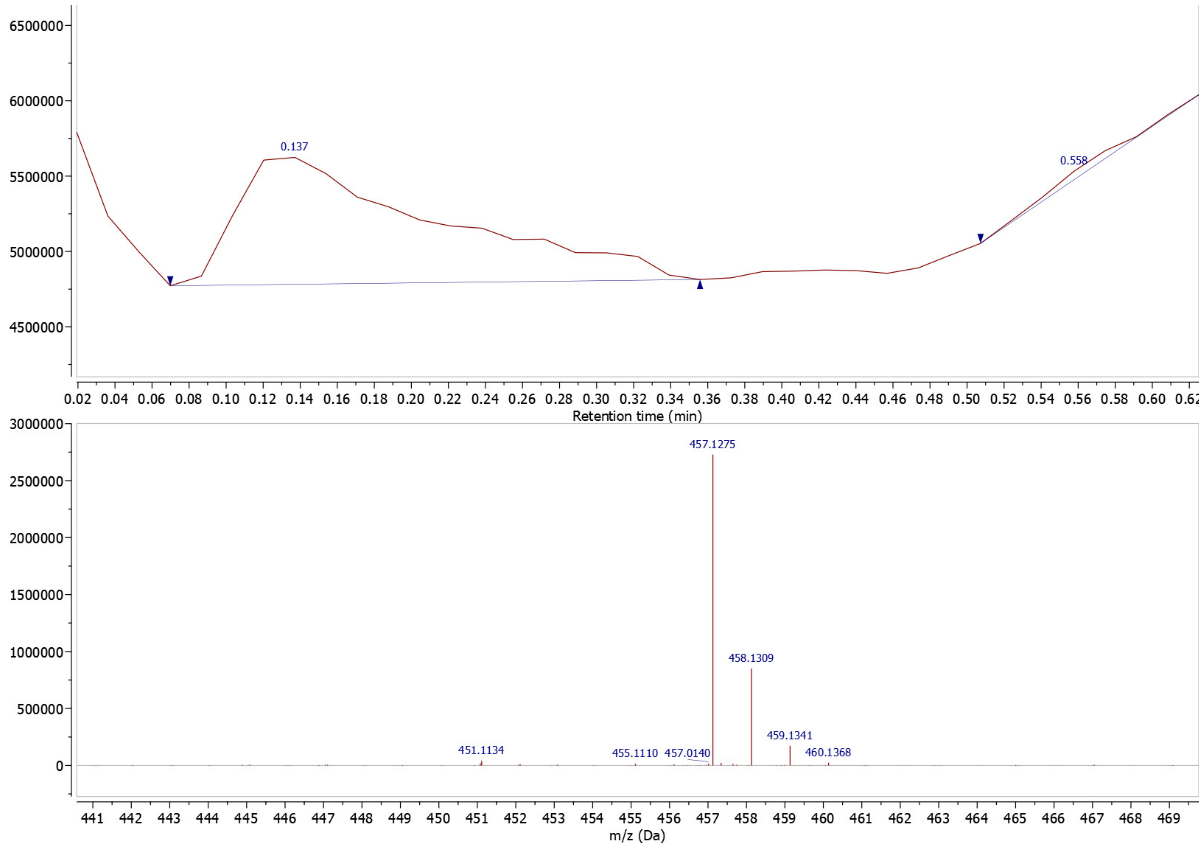

Figure S60. HR-ESI-MS spectrum of compound **8**.

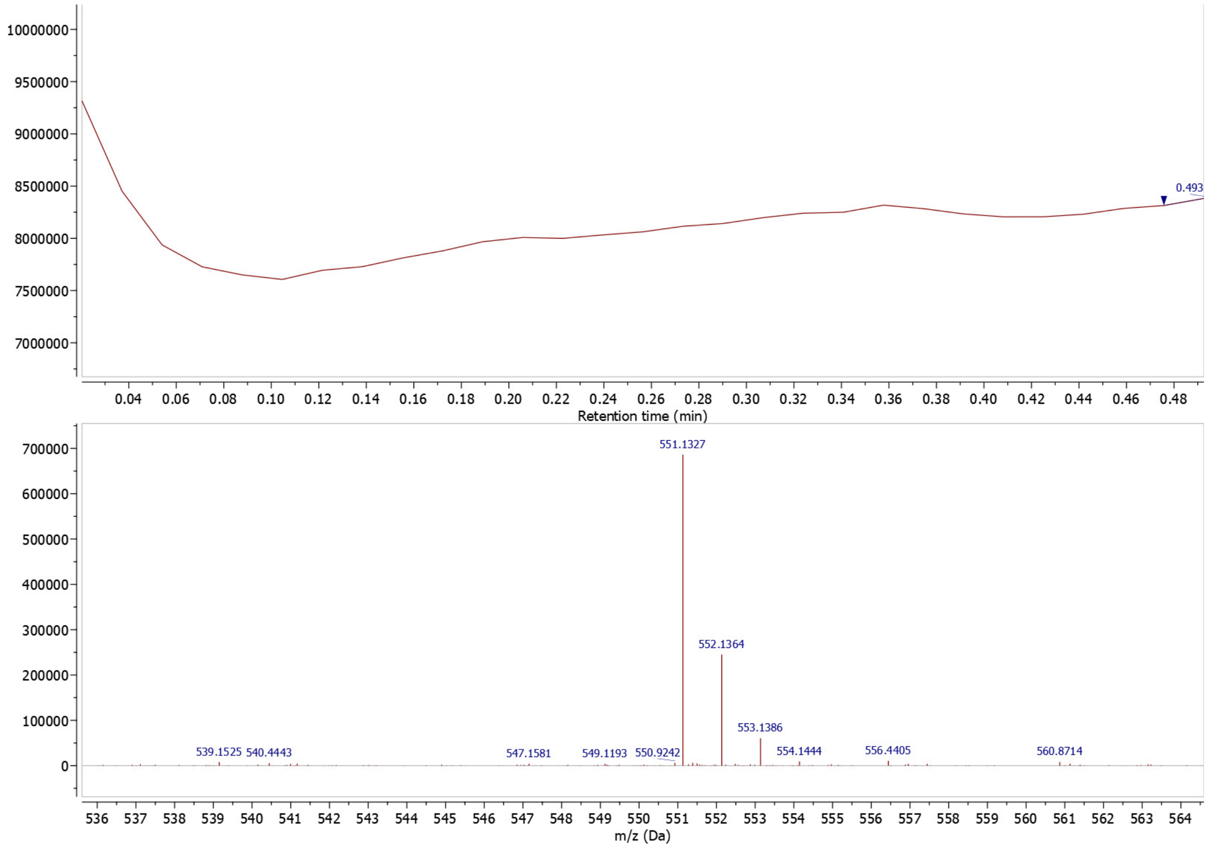

Figure S61. HR-ESI-MS spectrum of compound **9**.

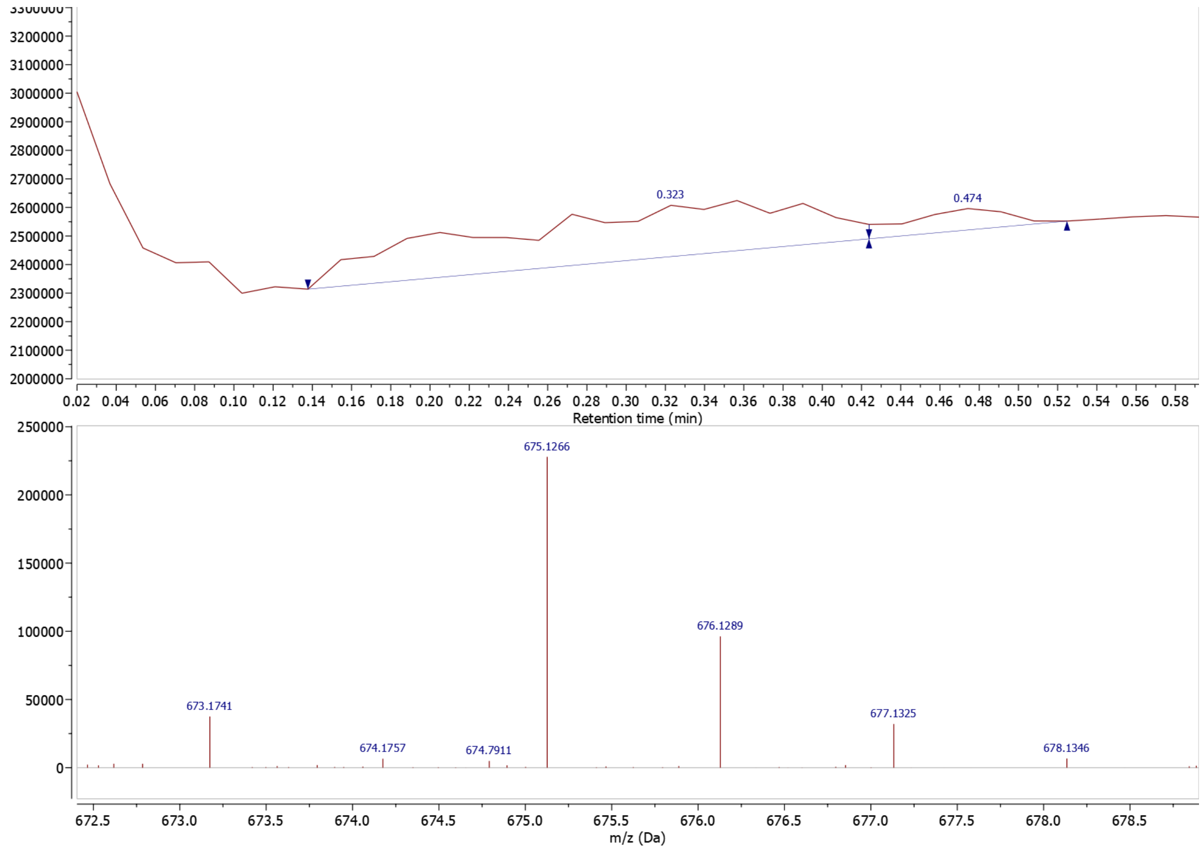

Figure S62. Isolation flowchart of compounds **1**-**22** from *H. aurantiacum*.

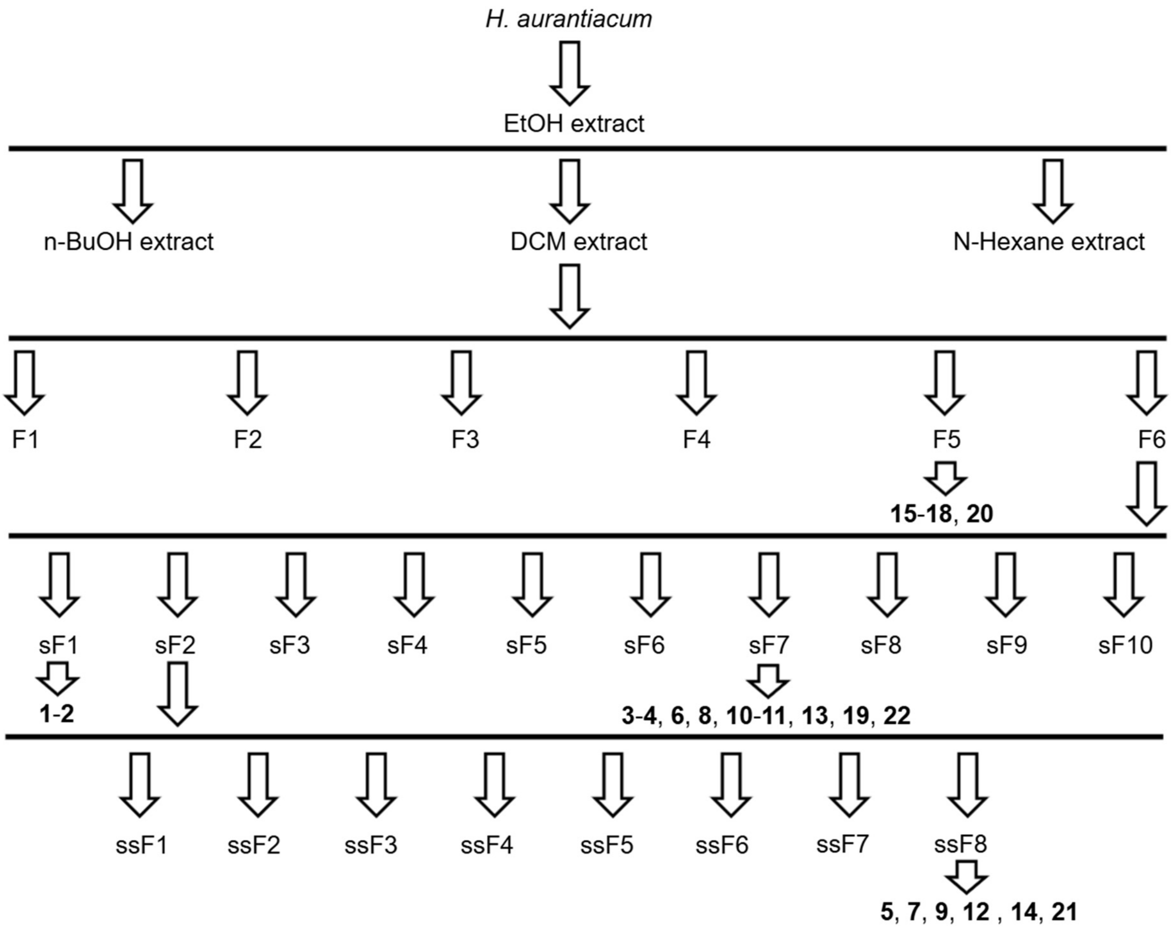

Supplement: Supplementary file 1 [file molecules-31-02175-s001.zip › molecules-4357432-supplementary.pdf]
